# Global burden of pneumoconiosis from 1990 to 2021: a comprehensive analysis of incidence, mortality, and socio-demographic inequalities in 204 countries and territories

**DOI:** 10.3389/fpubh.2025.1579851

**Published:** 2025-04-23

**Authors:** Shenyu Zhang, Jun Xiong, Xinyi Ruan, Chongyan Ji, Hanxin Lu

**Affiliations:** ^1^School of Public Administration, Hangzhou Normal University, Hangzhou, Zhejiang, China; ^2^School of Public Health and Nursing, Hangzhou Normal University, Hangzhou, Zhejiang, China

**Keywords:** pneumoconiosis, disease burden, GBD, decomposition analysis, inequality analysis, socio-demographic factor, Bayesian age-period-cohort

## Abstract

**Background:**

Pneumoconiosis, a group of occupational lung diseases caused by prolonged inhalation of mineral dust, remains a critical global health threat due to persistent workplace exposures in high-risk industries such as mining, construction, and artificial stone processing. These occupational hazards are exacerbated by inadequate dust control measures, insufficient use of personal protective equipment (PPE), and underreporting in low-and middle-income countries (LMICs). Emerging industries, including engineered stone fabrication, have introduced new risks, leading to accelerated silicosis among younger workers. Despite global efforts to improve occupational safety, socio-economic disparities, regulatory gaps, and public health crises such as the COVID-19 pandemic have further complicated disease management. This study analyzes trends in the global burden of pneumoconiosis from 1990 to 2021, providing evidence to inform post-pandemic strategies for occupational health equity and dust exposure mitigation.

**Methods:**

The data for this study were sourced from the Global Burden of Disease (GBD) 2021 database, utilizing age-standardized incidence rates (ASIR), prevalence rates (ASPR), mortality rates (ASDR), and disability-adjusted life years (DALYs) as the primary assessment indicators. Dynamic changes in the burden of pneumoconiosis were analyzed by estimating the annual percentage changes (EAPCs). The correlation between the Socio-Demographic Index (SDI) and the burden of pneumoconiosis was examined using Pearson correlation tests. Additionally, we conducted decomposition and inequality analyses and Bayesian Age-Period-Cohort (BAPC) to assess trends and distribution related to the pneumoconiosis burden.

**Results:**

The global incidence of pneumoconiosis increased from 42,187.99 cases in 1990 to 62,866.45 cases in 2021, accompanied by a rise in mortality rates. Notably, the burden of pneumoconiosis remains disproportionately higher among men than women across nearly all regions. The highest incidence and mortality rates were recorded in the age group of 80 years and older, with a pronounced gender disparity, particularly in East Asia and High-income North America. These rates were generally elevated in low-income and lower-middle-income regions, where males exhibited significantly higher ASIR and ASDR compared to females. No correlation was found between the SDI values and the pneumoconiosis burden. Additionally, absolute inequality among SDI countries decreased from 1990 to 2021, whereas relative inequality demonstrated an upward trend during the same period.

## Introduction

Pneumoconiosis, a spectrum of occupational interstitial lung diseases caused by prolonged inhalation of mineral dust, continues to pose a significant global public health challenge despite advancements in occupational safety protocols. This condition is characterized by chronic pulmonary inflammation and progressive fibrosis, ultimately leading to respiratory failure and premature mortality. It imposes substantial socioeconomic burdens on affected individuals and healthcare systems worldwide (1). The primary etiological agents include crystalline silica, coal dust, asbestos, and other inorganic particulates, with silicosis, coal workers’ pneumoconiosis (CWP), and asbestosis being the most prevalent forms (2, 3).

In 2021, an estimated 527,500 cases of pneumoconiosis were reported globally, with over 60,000 new diagnoses each year and a persistently high mortality rate of over 21,000 deaths annually since 2015 (4). While developed nations have experienced declining incidence rates due to stringent occupational regulations, low-and middle-income countries (LMICs) face increasing burdens driven by rapid industrialization, inadequate dust control measures, and underreporting (5, 6). For instance, China accounts for nearly half of the global pneumoconiosis cases, with over 90,000 cumulative occupational cases reported by 2021, predominantly in the mining and manufacturing sectors (7). Alarmingly, even countries with robust healthcare systems, such as the United States and Australia, have observed resurgences in silicosis and CWP linked to emerging industries like artificial stone processing and denim sandblasting (7).

In 2017, the Disability-Adjusted Life Years (DALYs) attributable to pneumoconiosis reached 507,425 globally, with low-and middle-income countries (LMICs) bearing 75% of this burden (8). Silicosis and coal workers’ pneumoconiosis (CWP) contribute disproportionately, particularly in regions with extensive mining activities. For instance, China’s DALYs for pneumoconiosis totaled 247,619 person-years in 2017, while South Africa and Chile exhibit elevated mortality rates among gold and copper miners, respectively (9, 10). Additionally, pneumoconiosis-associated cancers of the trachea, bronchus, and lung—recognized by the International Agency for Research on Cancer (IARC) as silica-related malignancies—further compound the disease burden, particularly among aging populations with prolonged dust exposure (11).

Socioeconomic determinants significantly influence the distribution of this disease. Workers in informal sectors, migrants, and individuals in LMICs often lack access to protective equipment, health surveillance, and compensation systems, exacerbating health inequities (12). Delayed diagnosis, frequently occurring decades after initial exposure, limits therapeutic efficacy, as advanced-stage patients face median survival times of 6–9 years post-diagnosis (6, 13). Despite innovations in diagnostic imaging (e.g., high-resolution CT) and emerging therapies (e.g., pirfenidone, stem cell transplantation), no curative treatments currently exist, highlighting the urgent need for primary prevention (14, 15).

The Global Burden of Disease (GBD), established in 1990, provides a scientific analytical framework and comprehensive data for assessing the global and regional burden of diseases, injuries, and risk factors (16). Utilizing extensive epidemiological datasets and systematic modeling approaches, the GBD initiative evaluates the incidence, prevalence, mortality, and disability-adjusted life years (DALYs) associated with a wide range of health conditions, including pneumoconiosis. A distinctive feature of the GBD framework is its hierarchical classification of regions, which divides the world into seven super-regions based on epidemiological patterns and geographic proximity. These super-regions are further subdivided into 21 specific regions, such as South Asia, Eastern Europe, and Sub-Saharan Africa, enabling granular analyses of disease distribution and determinants. This stratification not only highlights variations in health outcomes across diverse populations but also serves as a critical foundation for evidence-based policymaking. By integrating the Socio-Demographic Index (SDI) and risk factor attributions, the GBD facilitates the identification of disparities in disease burden linked to socioeconomic development, healthcare infrastructure, and public health interventions. Its methodologies and outputs have become indispensable tools for guiding national and global strategies aimed at mitigating health challenges and efficiently allocating resources.

This study, utilizing data from the GBD 2021 database, aims to evaluate the current status and changes in the global and regional burden of pneumoconiosis by analyzing its incidence, prevalence, and mortality rates over time. By examining the 21 regions defined by the GBD, the study also explores the influence of gender, age, and socioeconomic development on the burden of pneumoconiosis. The study aspires to serve as a valuable resource for global pneumoconiosis control efforts, with findings intended to inform more effective strategies for prevention, thereby contributing to the global goal of eliminating the pneumoconiosis epidemic by 2030 (6).

## Methods

The data sources and computational algorithms utilized for this analysis were derived from a systematic review of epidemiological literature and validated Global Burden of Disease (GBD) repositories (17, 18). Methodological refinements were applied to specifically address the parameters related to pneumoconiosis. Adjustments were made in accordance with the GBD 2021 analytical protocols, ensuring compliance with occupational health surveillance standards while maintaining contextual relevance and epidemiological validity.

### Data acquisition

The Global Burden of Disease 2021 (GBD 2021) systematically quantifies the health burden of 371 diseases and injuries across 204 countries and territories (19), along with subnational estimates for 21 regions (20). Its analytical framework, which includes disease classification hierarchies, analytical models, and uncertainty assessments, is rigorously documented in prior publications (21). Metrics of disease burden—such as incidence, prevalence, mortality, Disability-Adjusted Life Years (DALYs), and age-standardized rates (ASRs)—were sourced from the Global Health Data Exchange (GHDx). Additionally, sex-and age-stratified data were collected. The Socio-Demographic Index (SDI), a composite measure of fertility, income, and education, was utilized to evaluate the associations between the burden of pneumoconiosis and the level of social development, based on previous studies.

### Statistical analysis

Incidence, prevalence, mortality, and disability-adjusted life years (DALYs) serve as primary metrics for assessing the burden of pneumoconiosis. To adjust for variations in population demographics, age-standardized incidence rate (ASIR), prevalence rate (ASPR), mortality rate (ASDR), and DALY rate were calculated. Temporal trends in disease burden were evaluated using estimated annual percentage changes (EAPCs), derived from a regression model applied to age-standardized rates (ASRs) with the equation: y = *α* + βx. Here, x represents the year, and y represents log10 (ASRs). The EAPC value is derived from EAPC = 100*(10^β^ − 1), with EAPC values and 95% confidence intervals (CIs) above zero indicating an upward trend. Additionally, Pearson’s correlation coefficient was used to quantify associations between Socio-Demographic Index (SDI) and ASRs.

### Decomposition analysis

The Das Gupta decomposition method was employed to disaggregate the temporal changes in the burden of pneumoconiosis from 1990 to 2021 into independent contributions from aging, population growth, and epidemiological changes. This method effectively isolates the effects of demographic shifts, such as aging, and dynamics of disease risk, thereby providing detailed insights into the drivers of long-term trends (22). In contrast to linear regression, which primarily emphasizes variable associations, decomposition analysis quantifies the proportional impact of each factor on changes in the total burden, thereby clarifying their respective roles in shaping the global epidemiology of pneumoconiosis.

### Inequality analysis

The slope index of inequality (SII) was calculated by regressing the country-level age-standardized prevalence of pneumoconiosis against a sociodemographic development-related relative position scale, which is defined as the midpoint of cumulative population percentiles ranked by the Socio-Demographic Index (SDI). This approach quantifies absolute socioeconomic inequality in disease burden. The concentration index (CI) was derived by fitting a Lorenz concentration curve to the cumulative distribution of disease prevalence across populations ordered by SDI. Subsequently, numerical integration of the area under the curve was performed to measure relative inequality (23). Both indices assess disparities in the burden of pneumoconiosis attributable to sociodemographic gradients.

### Age-period-cohort analysis

To disentangle the independent and interactive effects of age, period, and birth cohort on the temporal trends in age-standardized incidence (ASIR) and death rates (ASDR) of pneumoconiosis from 1990 to 2021, this study employed Bayesian Age-Period-Cohort (BAPC) models. This advanced statistical framework integrates Bayesian inference with traditional age-period-cohort (APC) analysis, enabling robust estimation of posterior distributions for age, period, and cohort effects by combining prior information about unknown parameters with observed data (24). The BAPC approach complements existing decomposition and inequality analyses by isolating how demographic aging, generational exposures, and time-specific factors collectively drive the burden of pneumoconiosis.

### Data visualization

Data visualization was performed using the R software package (version 4.2.3) and JD_GBDR (V2.36; Jingding Medical Technology Co., Ltd.) to visualize the global burden of pneumoconiosis through world maps. Specific R packages, including map, ggplot2, and dplyr, for example, were utilized in this analysis.

## Results

### Pneumoconiosis incidence, prevalence, and annual percentage change

The global number of incident cases of pneumoconiosis increased from 42,187.99 in 1990 (95% CI: 35,785.81 to 48,912.75) to 62,866.45 in 2021 (95% CI: 54,616.86 to 71,103.37) (Table 1). When examining gender differences, the total number of incident cases for males in 2021 was 53,579.74 (95% CI: 46,529.69 to 60,653.93), while for females it was 9,286.71 (95% CI: 7,640.48 to 11,171.99). Despite variations in population size and age structure, the incidence rates of pneumoconiosis remained consistently higher in males than in females. In 2021, the age-standardized incidence rate (ASIR) for males was 1.36 per 100,000 population (95% CI: 1.19 to 1.53), whereas the ASIR for females was 0.21 per 100,000 population (95% CI: 0.17 to 0.25). Notably, the changes in incidence rates between males and females demonstrated a significant difference. The estimated annual percentage change (EAPC) for males was −0.07 (95% CI: −0.17 to 0.03), while for females it was 0.41 (95% CI: 0.33 to 0.50).

**Table 1:** The number of incident cases and incidence rates of pneumoconiosis in 1990/2021 and temporal trends.

Characteristics	1990	1990	2021	2021	1990–2021
	Incident cases no. (95%CI)	ASIR/100,000 no. (95%CI)	Incident cases no. (95%CI)	ASIR/100,000 no. (95%CI)	EAPC no. (95%CI)
Overall	42187.99 (35785.81, 48912.75)	1.03 (0.88, 1.19)	62866.45 (54616.86, 71103.37)	0.74 (0.64, 0.83)	−0.02 (−0.09, 0.06)
Sex					
Male	36767.89 (31292.79, 42478.57)	1.99 (1.70, 2.30)	53579.74 (46529.69, 60653.93)	1.36 (1.19, 1.53)	−0.07 (−0.17, 0.03)
Female	5420.10 (4335.94, 6689.62)	0.24 (0.19, 0.30)	9286.71 (7640.48, 11171.99)	0.21 (0.17, 0.25)	0.41 (0.33, 0.50)
Socio-demographic factor					
High SDI	10324.22 (8804.25, 11891.67)	0.94 (0.81, 1.08)	14286.99 (12638.72, 16090.65)	0.70 (0.62, 0.79)	0.33 (0.26, 0.40)
High-middle SDI	12194.24 (10331.41, 14138.47)	1.20 (1.02, 1.39)	16082.58 (13837.03, 18333.65)	0.84 (0.72, 0.96)	0.13 (0.08, 0.18)
Middle SDI	14440.41 (12125.40, 16944.37)	1.28 (1.08, 1.50)	22871.26 (19520.90, 26182.10)	0.85 (0.73, 0.97)	0.34 (0.21, 0.46)
Low-middle SDI	3995.32 (3343.58, 4698.59)	0.60 (0.51, 0.69)	7285.36 (6235.60, 8482.45)	0.48 (0.41, 0.55)	0.33 (0.25, 0.40)
Low SDI	1201.28 (993.65, 1408.14)	0.50 (0.43, 0.58)	2311.14 (1976.52, 2681.52)	0.41 (0.35, 0.46)	−0.50 (−0.58, −0.43)
Region					
Andean Latin America	128.37 (111.36, 145.82)	0.62 (0.54, 0.70)	251.76 (222.35, 283.65)	0.43 (0.38, 0.48)	0.69 (0.55, 0.82)
Australasia	85.27 (75.82, 95.40)	0.36 (0.32, 0.40)	294.88 (271.23, 325.37)	0.52 (0.47, 0.57)	2.99 (2.77, 3.22)
Caribbean	62.35 (49.44, 76.89)	0.23 (0.18, 0.28)	94.27 (73.15, 119.15)	0.18 (0.14, 0.23)	0.41 (0.32, 0.50)
Central Asia	208.30 (172.73, 250.52)	0.42 (0.36, 0.50)	324.34 (273.49, 383.89)	0.39 (0.33, 0.45)	0.43 (0.37, 0.48)
Central Europe	1391.78 (1200.64, 1599.34)	0.93 (0.80, 1.06)	964.67 (833.31, 1120.73)	0.51 (0.43, 0.59)	−0.78 (−0.87, −0.69)
Central Latin America	901.14 (746.96, 1077.20)	0.94 (0.78, 1.10)	1698.12 (1414.02, 2003.66)	0.66 (0.55, 0.78)	0.45 (0.39, 0.52)
Central Sub-Saharan Africa	113.79 (93.87, 131.99)	0.54 (0.46, 0.62)	233.31 (197.91, 270.96)	0.43 (0.38, 0.49)	−0.77 (−0.82, −0.73)
East Asia	20006.16 (16643.04, 23629.50)	2.18 (1.82, 2.56)	30658.22 (26241.78, 35283.38)	1.41 (1.22, 1.62)	0.73 (0.61, 0.86)
Eastern Europe	1215.57 (973.45, 1462.00)	0.44 (0.36, 0.53)	920.14 (752.94, 1088.22)	0.29 (0.24, 0.34)	−0.94 (−1.26, −0.61)
Eastern Sub-Saharan Africa	413.98 (341.87, 482.03)	0.55 (0.46, 0.63)	727.12 (622.77, 834.68)	0.41 (0.36, 0.46)	−1.00 (−1.12, −0.88)
High-income Asia Pacific	1809.32 (1537.08, 2088.49)	0.90 (0.77, 1.03)	2872.71 (2540.06, 3254.76)	0.62 (0.55, 0.70)	0.99 (0.85, 1.14)
High-income North America	2699.01 (2187.26, 3207.47)	0.77 (0.62, 0.91)	4505.40 (3866.34, 5189.15)	0.69 (0.59, 0.79)	1.07 (0.73, 1.41)
North Africa and Middle East	797.12 (639.24, 999.38)	0.36 (0.29, 0.44)	1665.54 (1337.44, 2052.14)	0.32 (0.26, 0.38)	0.64 (0.36, 0.93)
Oceania	15.61 (12.35, 19.56)	0.53 (0.43, 0.63)	43.46 (36.35, 51.40)	0.64 (0.54, 0.75)	1.06 (0.92, 1.20)
South Asia	4070.88 (3383.75, 4774.53)	0.66 (0.56, 0.77)	7782.96 (6645.36, 9083.43)	0.50 (0.43, 0.58)	0.43 (0.32, 0.54)
Southeast Asia	1041.53 (815.31, 1324.91)	0.32 (0.25, 0.40)	2172.60 (1721.19, 2699.60)	0.30 (0.24, 0.37)	1.23 (1.09, 1.38)
Southern Latin America	303.72 (271.19, 338.73)	0.65 (0.58, 0.73)	418.34 (377.29, 466.16)	0.48 (0.43, 0.54)	−0.10 (−0.26, 0.06)
Southern Sub-Saharan Africa	225.85 (188.97, 264.63)	0.81 (0.68, 0.95)	383.84 (331.69, 437.58)	0.69 (0.60, 0.78)	−0.08 (−0.37, 0.21)
Tropical Latin America	656.31 (547.48, 784.69)	0.62 (0.52, 0.74)	1263.00 (1102.20, 1434.86)	0.49 (0.43, 0.56)	0.52 (0.33, 0.72)
Western Europe	5887.13 (5124.81, 6672.31)	1.02 (0.89, 1.15)	5261.23 (4762.34, 5839.77)	0.56 (0.51, 0.63)	−0.92 (−0.98, −0.87)
Western Sub-Saharan Africa	154.80 (116.39, 199.20)	0.15 (0.11, 0.19)	330.53 (246.27, 432.85)	0.12 (0.10, 0.16)	−0.59 (−0.66, −0.53)

**Figure 1:**
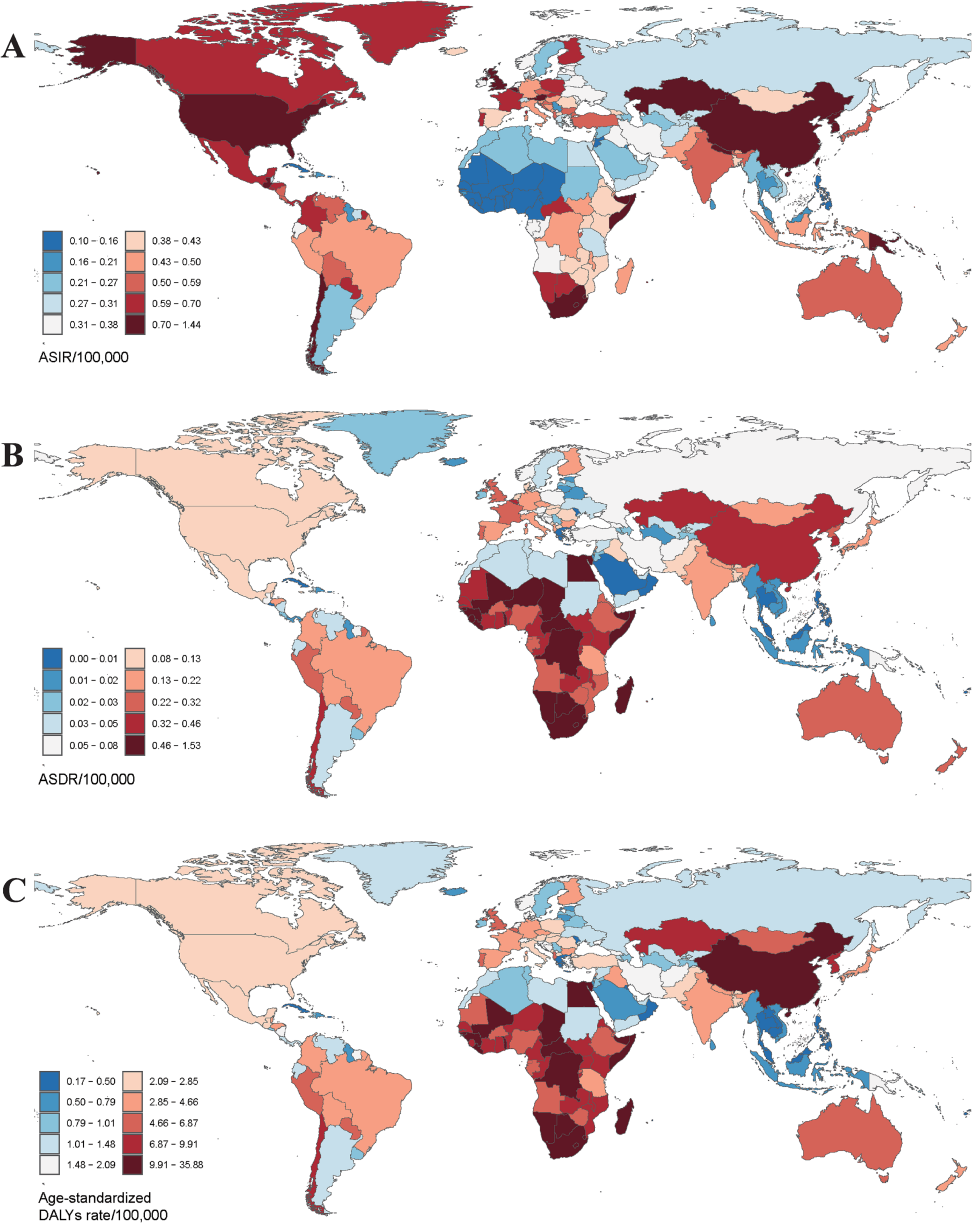
The global burden of pneumoconiosis in 204 countries or territories in 2021. **(A)** The ASIR of 204 countries; **(B)** the ASDR of 204 countries; **(C)** the age-standardized DALYs rate of 204 countries. ASIR, age-standardized incidence rate; ASDR, age-standardized death rate. Accurate ASRs was contained in Supplementary Table S1.

**Figure 2:**
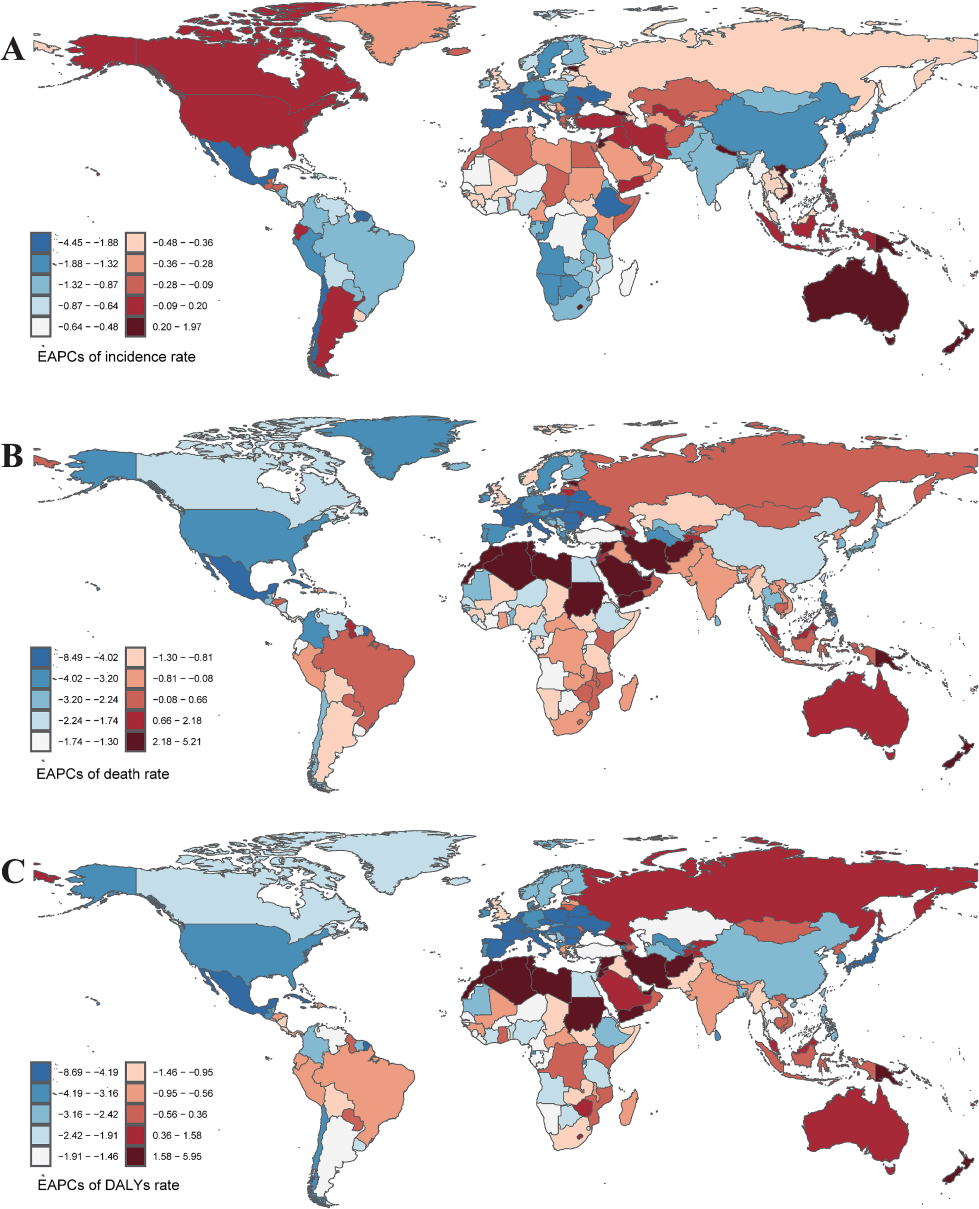
The EAPCs of incidence rate **(A)**, mortality rate **(B)**, and DALYs rate **(C)** caused by pneumoconiosis in 204 countries or territories from 1990 to 2021. EAPCs, Estimate the annual percentage change. Accurate ASRs was contained in Supplementary Table S2.

In terms of geographical distribution, East Asia reported the highest number of incident cases across both Asia and the 21 GBD regions, with 20,006.16 cases in 1990 and 30,658.22 cases in 2021. In contrast, Oceania recorded the lowest number of incident cases, totaling 43.46 in 2021. East Asia also exhibited the highest ASIR, with rates of 1.41 per 100,000 population in 2021 and 2.18 per 100,000 population in 1990. Conversely, Western Sub-Saharan Africa had the lowest ASIR among all GBD regions, at 0.12 per 100,000 population in 2021 (Table 1). Globally, the ASIR has declined over the past 30 years, with an EAPC of −0.02 (95% CI: −0.09 to 0.06); however, varying trends were observed across different regions. Eastern Sub-Saharan Africa experienced the most significant decline over the past three decades, with an EAPC of −1.00 (95% CI: −1.12 to −0.88), while Southern Sub-Saharan Africa exhibited the smallest reduction in ASIR among the GBD regions, with an EAPC of −0.08 (95% CI: −0.37 to 0.21). Furthermore, Australasia recorded the highest EAPC across all GBD regions, at 2.99 (95% CI: 2.77 to 3.22), accompanied by a substantial increase in ASIR, rising from 85.27 per 100,000 population in 1990 to 294.88 per 100,000 population in 2021 (Figure 1A). From a socio-demographic development perspective, most countries with a high Socio-Demographic Index (SDI) demonstrated an increasing trend in ASIR, while low SDI countries reported significantly fewer incidence cases and a lower ASIR, with 2,311.14 cases and 0.41 per 100,000 population in 2021, and an EAPC of −0.50 (95% CI: −0.58 to −0.43) (Figure 2A). The most rapid increase in ASIR was observed in middle SDI countries, which had an EAPC of 0.34 (95% CI: 0.21 to 0.46).

### Pneumoconiosis death and annual percentage change

The global mortality rate from pneumoconiosis has risen from 17,471.32 deaths in 1990 to 18,322.53 deaths in 2021. Gender-specific analysis in 2021 revealed that males experienced higher mortality rates and age-standardized death rates (ASDR) compared to females (deaths: male 16,621.31; female 1,701.23; ASDR: male 0.46 per 100,000 population; female 0.04 per 100,000 population), highlighting significant sex-based disparities (Table 2). Notably, females exhibited a marked increase in ASDR, while males showed a decline (male EAPC: −1.31, 95% CI: −1.34 to −1.27; female EAPC: 0.11, 95% CI: −0.01 to 0.23). An analysis of the Socio-Demographic Index (SDI) indicated that most countries with varying SDI levels demonstrated a declining trend in ASDR, with the exception of low-middle SDI countries, which had an EAPC of 0.24 (95% CI: 0.20 to 0.28). In 2021, pneumoconiosis deaths were predominantly concentrated in middle SDI countries (deaths: 5,701.04, 95% CI: 4,553.38 to 7,067.95), whereas in 1990, the majority of deaths were reported in high SDI countries (deaths: 5,653.97, 95% CI: 5,347.54 to 5,940.95). Despite middle SDI countries recording a higher absolute number of fatalities in 2021, the ASDR remained the highest in low SDI countries at 0.28 per 100,000 population (Table 2).

**Table 2:** The number of deaths and death rates of pneumoconiosis in 1990/2021 and temporal trends.

Characteristics	1990	1990	2021	2021	1990–2021
	Death cases no. (95% CI)	ASDR/100,000 no. (95% CI)	Death cases no. (95% CI)	ASDR	EAPC no. (95% CI)
Overall	17471.32 (15588.94, 19435.82)	0.46 (0.42, 0.51)	18322.53 (16041.35, 20916.37)	0.22 (0.19, 0.25)	−1.21 (−1.25, -1.17)
Sex					
Male	16390.19 (14572.39, 18301.12)	1.02 (0.92, 1.13)	16621.31 (14434.00, 19019.62)	0.46 (0.40, 0.52)	−1.31 (−1.34, -1.27)
Female	1081.13 (658.08, 1586.04)	0.05 (0.03, 0.08)	1701.23 (997.00, 2503.33)	0.04 (0.02, 0.05)	0.11 (−0.01, 0.23)
Socio-demographic factor					
High SDI	5653.97 (5347.54, 5940.95)	0.49 (0.47, 0.52)	4642.54 (4156.90, 4977.26)	0.19 (0.18, 0.21)	−1.56 (−1.62, -1.51)
High-middle SDI	5134.56 (4545.11, 5780.89)	0.54 (0.48, 0.61)	4537.47 (3699.87, 5539.63)	0.23 (0.19, 0.28)	−1.06 (−1.19, −0.94)
Middle SDI	4646.85 (3838.25, 5605.54)	0.46 (0.39, 0.55)	5701.04 (4553.38, 7067.95)	0.22 (0.18, 0.28)	−0.47 (−0.52, −0.43)
Low-middle SDI	1244.64 (793.98, 1705.79)	0.23 (0.15, 0.32)	2249.99 (1666.05, 2861.90)	0.17 (0.13, 0.22)	0.24 (0.20, 0.28)
Low SDI	777.44 (450.31, 1254.93)	0.41 (0.24, 0.66)	1185.16 (636.36, 1950.56)	0.28 (0.15, 0.46)	−1.24 (−1.33, -1.15)
Region					
Andean Latin America	51.58 (38.42, 66.61)	0.26 (0.20, 0.34)	116.79 (85.10, 162.03)	0.20 (0.15, 0.28)	1.13 (0.89, 1.37)
Australasia	44.56 (38.87, 51.21)	0.18 (0.16, 0.21)	189.50 (162.67, 218.74)	0.31 (0.27, 0.36)	3.49 (3.13, 3.85)
Caribbean	11.31 (9.85, 12.98)	0.05 (0.04, 0.05)	10.62 (8.57, 13.11)	0.02 (0.02, 0.02)	−1.28 (−1.82, −0.74)
Central Asia	89.73 (67.64, 118.60)	0.20 (0.15, 0.26)	94.20 (68.90, 128.51)	0.13 (0.09, 0.17)	−0.94 (−1.25, −0.62)
Central Europe	615.51 (558.35, 681.05)	0.41 (0.37, 0.45)	207.31 (185.50, 233.68)	0.09 (0.08, 0.10)	−3.51 (−3.77, -3.25)
Central Latin America	227.13 (215.45, 239.32)	0.30 (0.28, 0.32)	217.94 (190.27, 243.96)	0.09 (0.08, 0.10)	−1.94 (−2.12, -1.76)
Central Sub-Saharan Africa	95.36 (45.57, 163.17)	0.52 (0.24, 0.93)	198.68 (83.00, 397.67)	0.45 (0.17, 1.01)	−0.76 (−0.84, −0.68)
East Asia	7232.88 (5897.47, 8846.35)	0.88 (0.72, 1.06)	8496.44 (6605.69, 10956.77)	0.41 (0.32, 0.52)	−0.07 (−0.12, −0.01)
Eastern Europe	458.80 (376.67, 540.50)	0.17 (0.14, 0.19)	177.79 (159.86, 195.94)	0.05 (0.04, 0.06)	−2.34 (−3.08, -1.59)
Eastern Sub-Saharan Africa	297.21 (156.65, 509.68)	0.47 (0.24, 0.79)	440.81 (207.45, 747.98)	0.31 (0.14, 0.52)	−1.36 (−1.48, -1.24)
High-income Asia Pacific	1071.56 (997.99, 1147.79)	0.54 (0.50, 0.57)	1270.09 (1105.34, 1413.40)	0.22 (0.19, 0.25)	0.65 (0.38, 0.92)
High-income North America	1296.15 (1193.59, 1385.24)	0.34 (0.32, 0.37)	830.13 (738.81, 899.46)	0.12 (0.10, 0.13)	−2.59 (−2.67, -2.52)
North Africa and Middle East	284.81 (216.63, 403.75)	0.18 (0.13, 0.26)	452.43 (350.25, 593.81)	0.11 (0.08, 0.14)	−0.66 (−0.81, −0.51)
Oceania	0.50 (0.19, 1.10)	0.03 (0.01, 0.06)	2.50 (0.78, 6.12)	0.05 (0.02, 0.12)	3.56 (3.31, 3.80)
South Asia	946.92 (516.82, 1445.72)	0.20 (0.11, 0.30)	1919.17 (1301.18, 2659.80)	0.15 (0.10, 0.20)	0.49 (0.41, 0.57)
Southeast Asia	38.30 (25.96, 52.76)	0.02 (0.01, 0.02)	65.25 (42.93, 93.00)	0.01 (0.01, 0.02)	0.49 (0.32, 0.66)
Southern Latin America	128.88 (114.00, 148.89)	0.29 (0.25, 0.33)	146.46 (125.56, 168.11)	0.16 (0.14, 0.18)	−0.43 (−0.61, −0.24)
Southern Sub-Saharan Africa	141.83 (107.05, 179.18)	0.58 (0.44, 0.75)	250.85 (215.50, 287.35)	0.48 (0.42, 0.56)	0.44 (0.19, 0.69)
Tropical Latin America	154.29 (143.06, 166.20)	0.17 (0.15, 0.18)	414.05 (379.91, 446.14)	0.16 (0.15, 0.18)	2.21 (1.85, 2.57)
Western Europe	3807.52 (3563.25, 4043.60)	0.62 (0.58, 0.66)	2177.36 (1937.52, 2385.59)	0.19 (0.17, 0.20)	−2.38 (−2.44, -2.32)
Western Sub-Saharan Africa	476.50 (294.39, 756.66)	0.63 (0.39, 1.00)	644.16 (361.99, 1014.24)	0.40 (0.23, 0.62)	−1.88 (−1.97, -1.80)

Geographically, East Asia recorded the highest number of pneumoconiosis-related deaths, with rates of 7,232.88 in 1990 and 8,496.44 in 2021. In 2021, Southern Sub-Saharan Africa exhibited the highest ASDR at 0.48 per 100,000 population (95% CI: 0.42 to 0.56), while East Asia had the highest ASDR in 1990, recorded at 0.88 per 100,000 population (95% CI: 0.72 to 1.06) (Figure 1B). Notably, 13 out of 21 GBD regions showed a declining trend in ASDR. Central Europe experienced a sharp decline (EAPC: −3.51, 95% CI: −3.77 to −3.25), whereas Oceania exhibited a significant increase (EAPC: 3.56, 95% CI: 3.31 to 3.80) (Figure 2B). At the national and regional levels, the Kingdom of Eswatini and São Tomé and Príncipe reported relatively high ASDRs for pneumoconiosis, with rates of 1.52 and 1.09 per 100,000 population, respectively. Furthermore, Bermuda (EAPC: −8.41) and Saint Lucia (EAPC: −7.28) experienced significant decreases in ASDR due to pneumoconiosis, while Georgia (EAPC: 5.16) and New Zealand (EAPC: 5.00) saw increases in their ASDRs.

### Age distribution of pneumoconiosis

Age distribution is a critical factor in the epidemiology of pneumoconiosis. Global data from 2021 revealed significant variations in disease incidence, prevalence, and mortality across different age groups and between genders. It is important to note that the Global Burden of Disease (GBD) database does not provide pneumoconiosis burden data for individuals under the age of 15.

**Figure 3:**
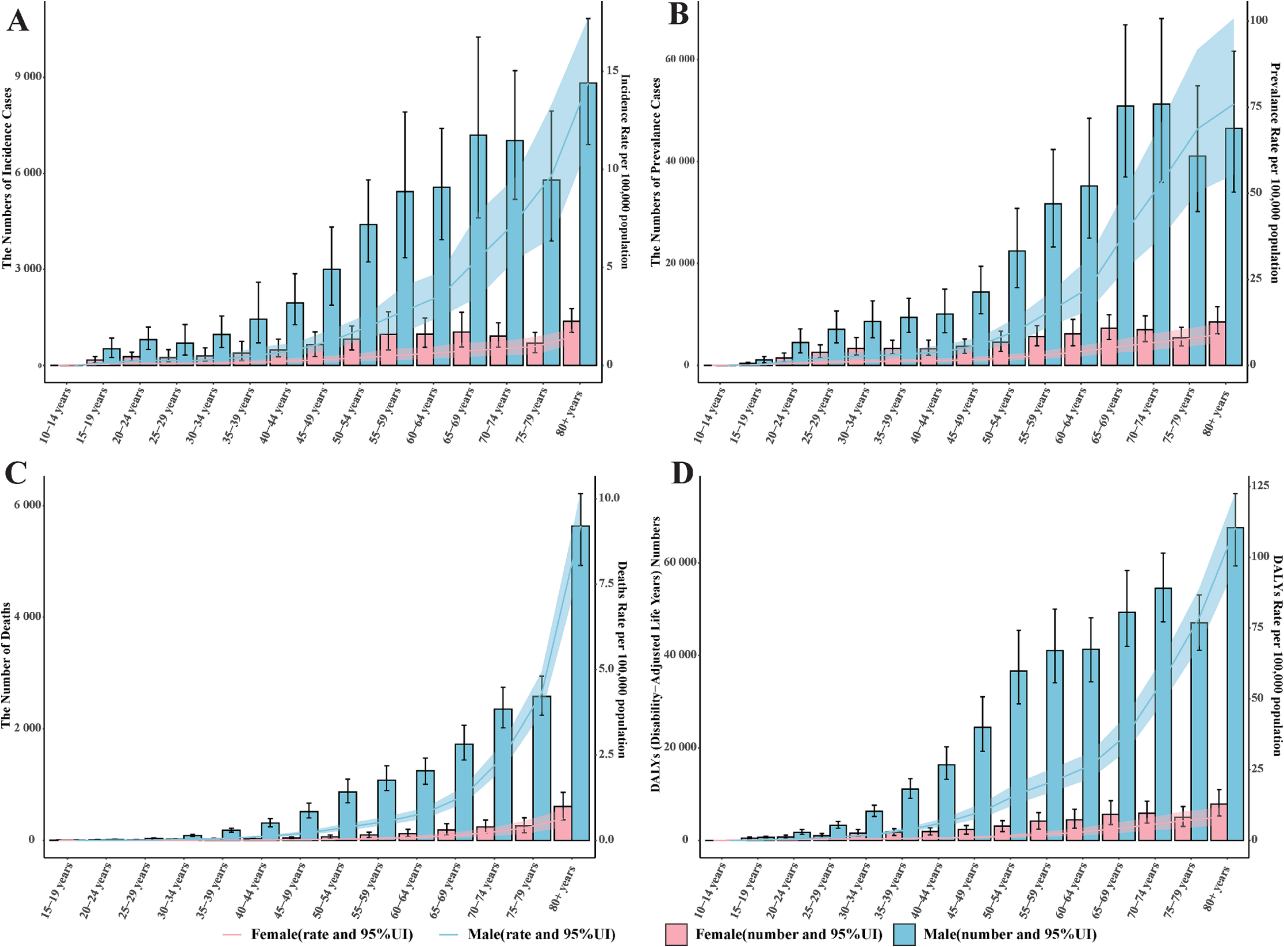
The global burden of pneumoconiosis by age group and gender in 2021. **(A)** The number of incident cases and incidence rate among different age groups. **(B)** the number of prevalent cases and prevalence rate among different age groups; **(C)** the number of deaths and death rate among different age groups; **(D)** the number and rate of DALYs caused by pneumoconiosis among different age groups. Accurate figures were contained in Supplementary Table S3.

The incidence rate of pneumoconiosis is lowest in the 15–19 age group, recorded at 0.05 per 100,000 population for males and 0.16 for females. Conversely, the incidence rate is highest in the 80 and older age group, with rates of 14.40 per 100,000 population in males and 1.45 in females (Figure 3A). The number of incident cases is also highest in the 80 and older group, with 8,821.86 cases for males and 1,381.80 cases for females. Across all age groups, both the number of patients diagnosed with pneumoconiosis and the incidence rates are higher in males compared to females. In 2021, the global prevalence of pneumoconiosis was most common among individuals aged 65–69 and 70–74. Specifically, there were 50,869.85 male cases and 7,289.76 female cases in the 65–69 age group, and 51,260.88 male cases and 6,977.34 female cases in the 70–74 age group, with corresponding incidence rates of 38.59, 5.06, 53.18, and 6.38 per 100,000 population, respectively.

Significant differences in age distribution were observed in both DALYs and mortality related to pneumoconiosis. The number of deaths and DALYs were markedly higher in males than in females across all age groups, with this disparity progressively widening with advancing age (Figure 3D). In contrast to DALYs, both the number of deaths and death rates demonstrated a rapid age-dependent increase, particularly among males, whose death rates escalated exponentially (Figure 3C). The peak values for both the number of deaths and death rates were recorded in the 80 and older group. With regard to pneumoconiosis-related DALYs, an upward trend was evident in the 15–54 age range, followed by a plateau during the 55–64 age interval. Subsequently, DALYs continued to rise, reaching their highest value in the 80 and older group.

### Global pneumoconiosis burden in different genders and regions

Using data from the Global Burden of Disease (GBD) 2021, population pyramids were constructed to analyze the age-gender stratification of the pneumoconiosis burden across 21 geographical regions. These pyramids effectively visualize sex-specific disparities in the age-standardized incidence rate (ASIR), prevalence rate (ASPR), mortality rate (ASDR), and DALY rate, (AS-DALYs).

**Figure 4:**
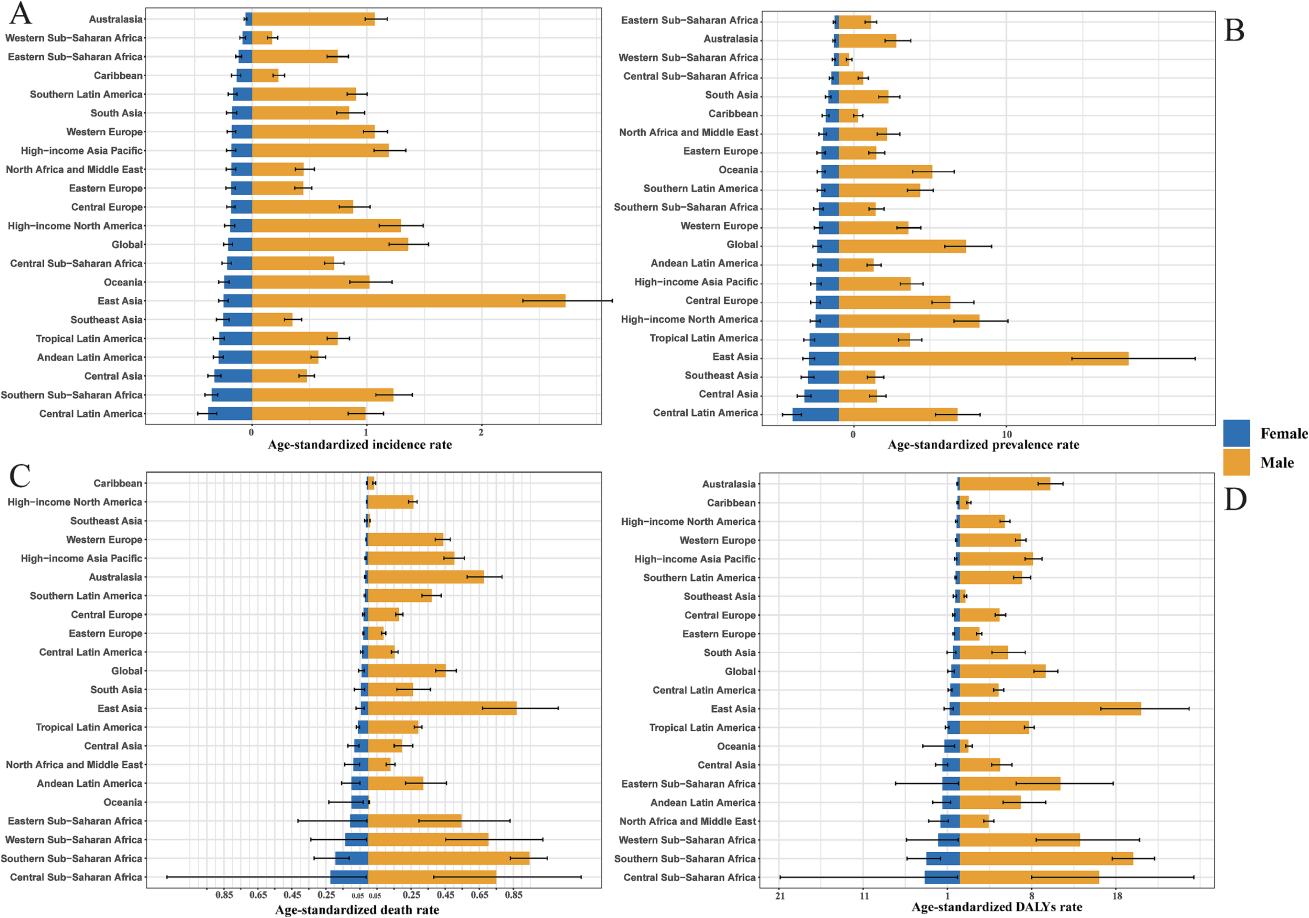
Gender-specific ASIR **(A)**, ASPR **(B)**, ASDR **(C)**, DALYs rate **(D)** of 21 GBD regions in 2021. Note accurate rate was contained in Supplementary Table S4.

Regional disparities in ASIR of pneumoconiosis were identified across the 21 GBD regions, with Central Latin America demonstrating the highest ASIR for females and East Asia exhibiting the highest ASIR for males (Figure 4A). Notably, significant gender disparities in pneumoconiosis burden were observed in East Asia and High-income North America, with ratios of males to females of 2.72 to 0.25 in East Asia and 9.24 to 1.50 in High-income North America.

The prevalence of pneumoconiosis is a critical epidemiological metric, serving as a quantitative proxy for the current disease burden. Central Latin America reported the highest ASPR for females at 3.02 per 100,000 population, while East Asia had the highest ASPR for males at 19.00 per 100,000 population, reflecting this region’s elevated incidence rate. Males exhibited a higher ASPR than females across all 21 GBD regions (Figure 4B). In contrast, Western Sub-Saharan Africa recorded the lowest ASPR for males at 0.68 per 100,000 population, and Eastern Sub-Saharan Africa had the lowest ASPR for females at 0.26 per 100,000 population. The gender disparity remains evident in East Asia and High-income North America, with male to female ratios of 19.00 to 1.94 in East Asia and 1.30 to 0.19 in High-income North America.

In comparison to ASIR and ASPR, the ASDR and age-standardized DALY rates exhibited more pronounced gender differences. Globally, the ASDR and age-standardized DALY rates for pneumoconiosis were significantly higher in males than in females. The Caribbean recorded the lowest ASDR for females (0.0069 per 100,000 population), while Oceania had the lowest ASDR for males (0.0056 per 100,000 population). Central Sub-Saharan Africa reported the highest ASDR for females (0.22 per 100,000 population), and Southern Sub-Saharan Africa had the highest ASDR for males (0.95 per 100,000 population) (Figure 4C). Notably, in Oceania and Southeast Asia, males with pneumoconiosis demonstrated lower ASDR compared to females, a trend that markedly deviates from the global pattern of higher male ASDR across all regions. Additionally, Central Sub-Saharan Africa had the highest age-standardized DALY rate for females (4.15 per 100,000 population), while East Asia had the highest age-standardized DALY rate for males (21.49 per 100,000 population) (Figure 4D). Consistent with the ASDR pattern, males with pneumoconiosis in Oceania exhibited a lower age-standardized DALY rate compared to females, further diverging from the global trend of higher male age-standardized DALY rates across all regions.

### The correlation analysis between SDI value and the pneumoconiosis burden

**Figure 5:**
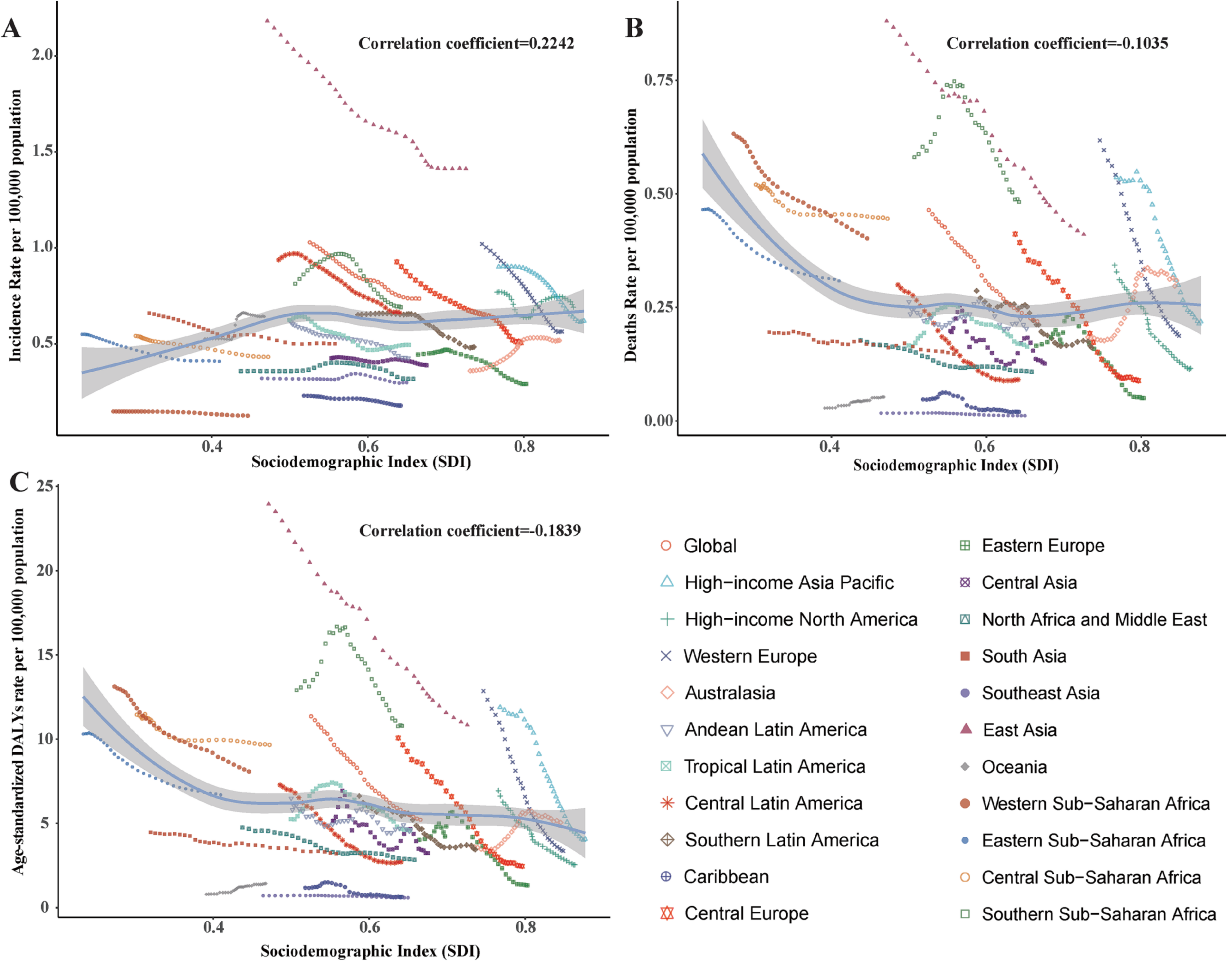
Correlation between socio-demographic index (SDI) and pneumoconiosis Burden in 2021. **(A)** The incidence rate per 100,000 population; **(B)** the mortality rate per 100,000 population; **(C)** the DALYs rate per 100,000 population. Accurate SDI from 1990 to 2021 was contained in Supplementary Table S5.

To investigate potential associations between sociodemographic development levels and the burden of pneumoconiosis, Spearman rank-order correlation analyses were conducted to assess the relationship between SDI values and age-standardized rates (ASRs) across 21 geographic regions from 1990 to 2021 (Figure 5). The analysis found no statistically significant correlation between ASRs and SDI values, with the following coefficients: ASIR-SDI coefficient of 0.2242, ASDR-SDI coefficient of −0.1035, and age-standardized DALY rate-SDI coefficient of −0.1839; all *p* < 0.01.

### Cross country inequality analysis

**Figure 6:**
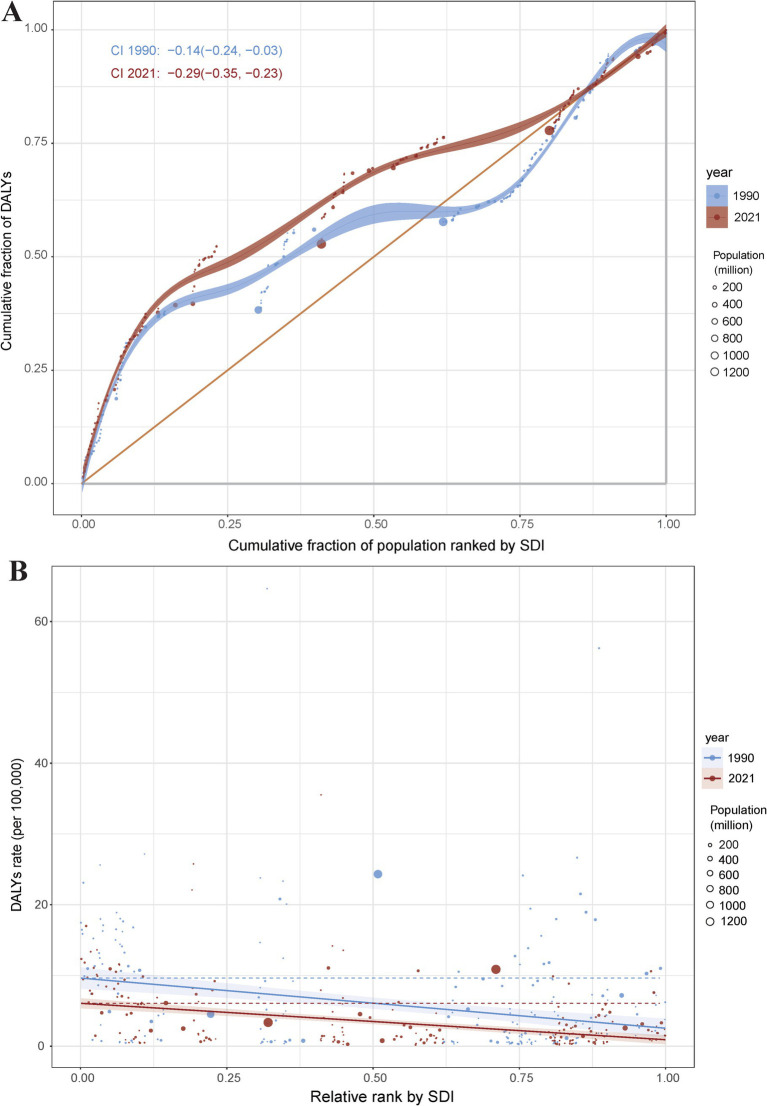
Health inequality concentration curves **(A)** and regression curves **(B)** for the DALYs of pneumoconiosis. Accurate data from 1990 and 2021 was contained in Supplementary Table S6.

In terms of the burden of pneumoconiosis, we observed significant absolute and relative inequalities associated with the SDI. In general, with countries and territories having lower SDI disproportionately bearing a higher burden. The concentration index was −0.14 (95% CI: −0.24 to −0.03) in 1990, but decreased to −0.29 (95% CI: −0.35 to −0.23) in 2021 (Figure 6A). In 1990, the slope index of inequality was −7.09 (95% CI: −9.61 to −4.58), by contrast, the slope index of inequality was −5.15 (95% CI: −6.44 to −3.86) in 2021 (Figure 6B). The findings demonstrate a decline in the concentration index (CI) coupled with an increase in the slope index of inequality (SII) for pneumoconiosis burden, indicating that absolute inequality across SDI countries decreased from 1990 to 2021, while relative inequality exhibited an upward trend over the same period.

### Decomposition analysis

**Figure 7:**
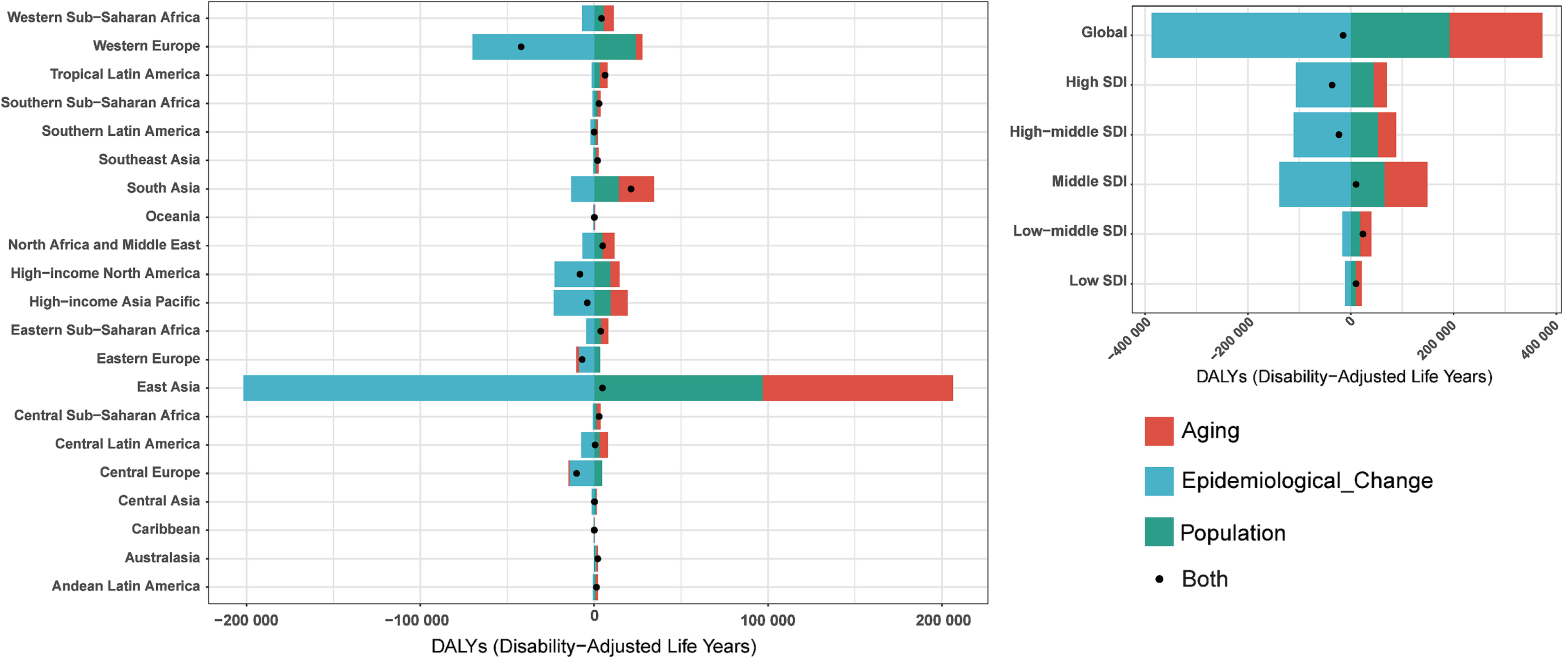
Changes in DALYs of pneumoconiosis according to population-level determinants including aging, population growth and epidemiological change from 1990 to 2019 at the global level and by SDI quintiles and regions stratified by sexes. Accurate data was contained in Supplementary Table S7.

To quantify the contributions of population aging, demographic growth, and epidemiological transition to pneumoconiosis epidemiology, we conducted a decomposition analysis of incident case counts, incorporating demographic changes, age-structural shifts, and age-standardized morbidity and mortality rates. Globally, the total disability-adjusted life years (DALYs) for pneumoconiosis decreased by 14,898.79, with aging contributing −1,211.15%, population growth contributing −1,287.18%, and epidemiological changes contributing 2,598.33% (Figure 7). When stratified by Sociodemographic Index (SDI) categories, low-middle SDI countries exhibited an overall increase of 23,757.15 DALYs, driven by aging (89.9%), population growth (76.87%), and a negative contribution from epidemiological changes (−66.76%). In contrast, high SDI countries demonstrated a substantial decline of 36,178.84 DALYs, with aging (−70.86%) and population growth (−122.32%) showing negative contributions, while epidemiological changes accounted for 293.19% of the reduction. From the perspective of 21 GBD regions, Western Europe demonstrated a marked decline, with pneumoconiosis-related DALYs decreasing by 42,048.82, where aging contributed −9.18%, population growth contributed −56.93%, and epidemiological changes accounted for 166.11%. Conversely, South Asia experienced a substantial increase, with DALYs rising by 21,184.59, driven by aging (95.86%), population growth (66.71%), and a negative contribution from epidemiological changes (−62.57%).

### Prediction of pneumoconiosis incidence and mortality rate to 2035

**Figure 8:**
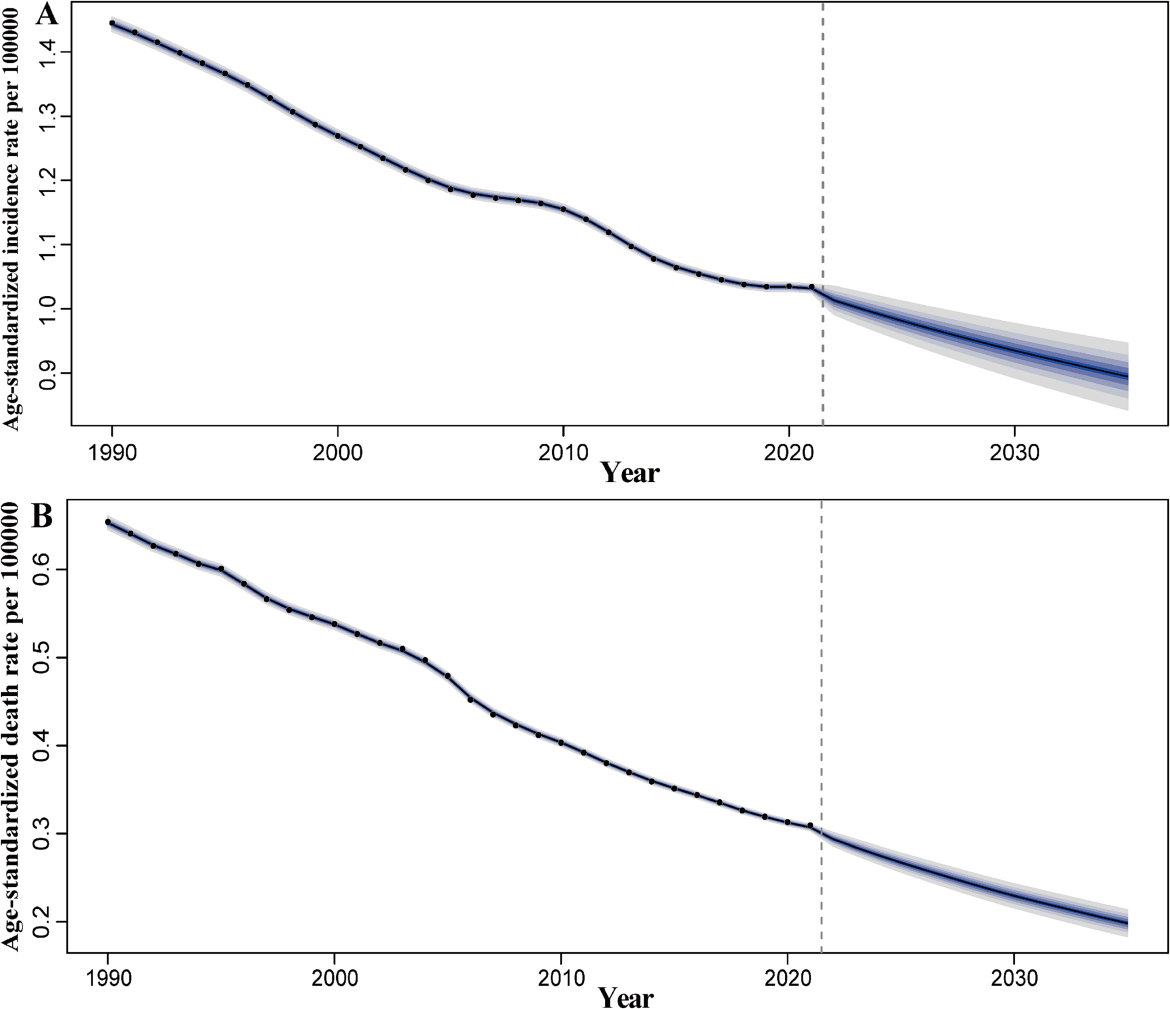
Predicted global trends of pneumoconiosis ASIR and ASDR from 2021 to 2035. **(A)** ASIR; **(B)** ASDR. Accurate data was contained in Supplementary Table S8.

Overall, from 1990 to 2021, the ASIR and ASDR of pneumoconiosis exhibited a declining trend annually. Notably, the decline in ASIR slowed between 2006 and 2010, followed by an accelerated reduction over the subsequent 4–5 years. In contrast, the ASDR demonstrated a relatively stable downward trajectory compared to ASIR. Projections indicate that both the ASIR and ASDR of pneumoconiosis are expected to continue decreasing (Figure 8). According to the prediction, ASIR will be 0.93 (95% CI: 0.89 to 0.98) in 2030 and 0.89 (95% CI: 0.83 to 0.96) in 2035. ASDR on the other hand, ASDR will drop to 0.23 (95% CI: 0.21 to 0.25) in 2030, and 0.20 (95% CI: 0.18 to 0.22) in 2035.

**Figure 9:**
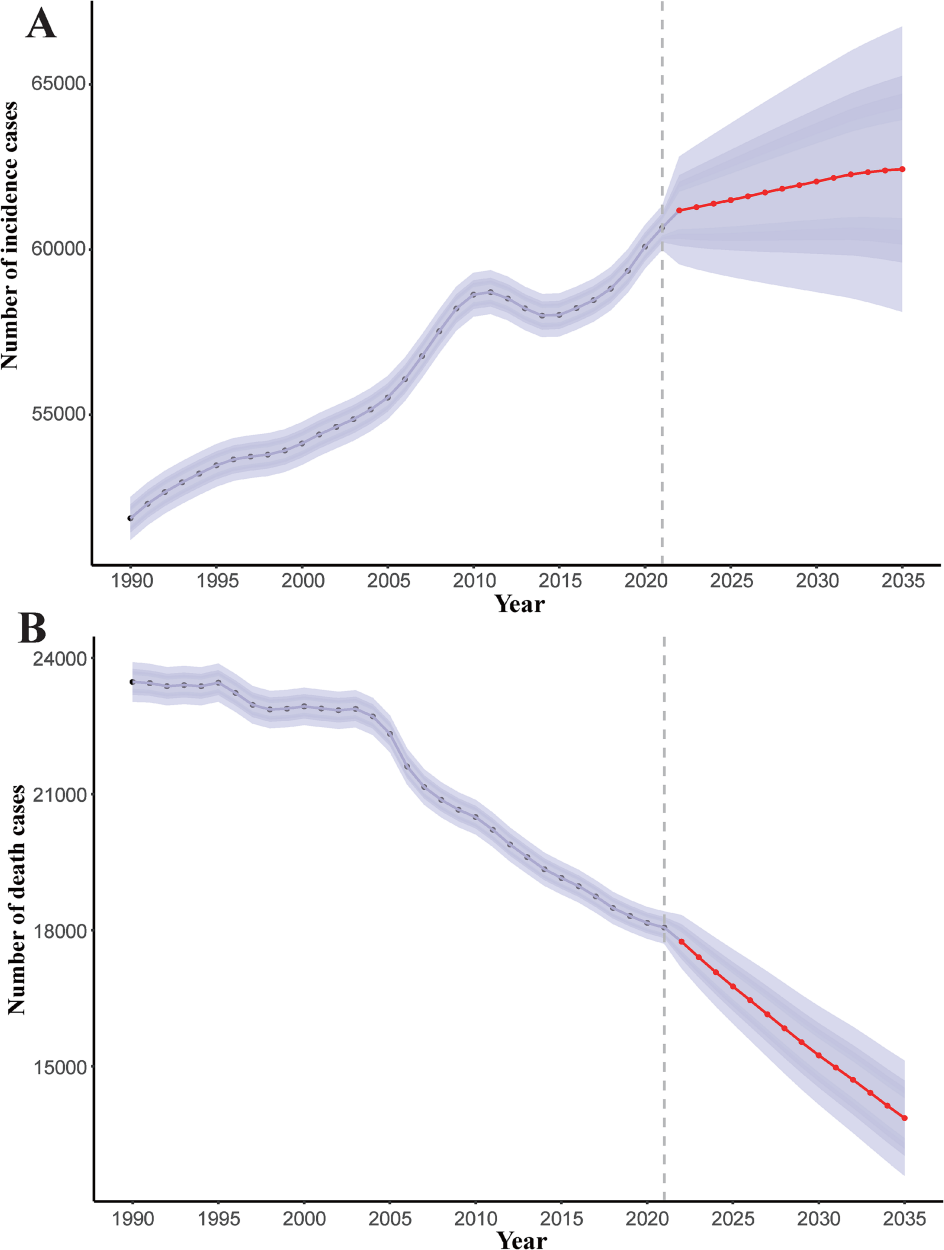
Predicted global trends of number of incidence cases and death cases of pneumoconiosis from 2021 to 2035. Accurate data was contained in Supplementary Table S9.

The contrasting trends in pneumoconiosis incidence (rising) and mortality (declining) observed from 1990 to 2021 reflect a complex interplay of occupational exposures, healthcare advancements, and evolving disease dynamics. From 1990 to 2021, the number of incidence cases and death cases of pneumoconiosis exhibited divergent trends (Figure 9). For incidence cases, an overall upward trajectory was observed. Specifically, the number of cases increased rapidly between 2006 and 2011, followed by a slight decline over the subsequent 4–5 years, after which it resumed an upward trend. Projection models indicate that the number of incidence cases will continue to rise over the next decade, albeit at a markedly reduced rate. It is predicted that the number of incidence cases will be 62060.86 (95% CI: 58718.87 to 65402.85) in 2030 and 62433.96 (95% CI: 58118.74 to 66749.18) in 2035. In contrast, the number of death cases demonstrated a downward trend. Between 1990 and 1995, mortality remained relatively stable with minimal fluctuations. A brief decline occurred between 1995 and 1997, followed by another period of stability over the next 6 years. Since 2004, mortality has again shown a sustained decline. Projection models suggest that the number of death cases will continue to decrease over the next decade, with the rate of decline accelerating significantly. The number of death cases will be 15245.78 (95% CI: 14165.40 to 16326.16) in 2030 and 13862.93 (95% CI: 12600.20 to 15125.66) in 2035.

## Discussion

### Overall global pneumoconiosis burden trends

The global burden of pneumoconiosis has exhibited complex trends over the past three decades. While high-income nations have reduced incidence through stringent occupational safety measures, the absolute number of incident cases increased from 42,187.99 in 1990 to 62,866.45 in 2021, driven by rapid industrialization in low-and middle-income countries (LMICs) and emerging industries such as artificial stone fabrication (2, 3). This observation aligns with the previous findings of a 66% global increase in pneumoconiosis cases from 1990 to 2017, despite a 0.6% annual decline in ASIR (2). Notably, silicosis and coal workers’ pneumoconiosis (CWP) decreased in middle and low SDI regions, while asbestosis surged in high SDI countries (12). Mortality trends diverged by gender: males experienced declining age-standardized death rates (ASDR: EAPC −1.31), whereas females showed a slight increase (EAPC 0.11), likely attributable to delayed diagnoses and limited healthcare access in informal sectors (6). These findings underscore the persistent role of occupational exposure as a primary driver of the pneumoconiosis burden (11), with previous evidence from the Global Burden of Disease (GBD) study indicates a global decline in silica-attributed mortality (ASMR EAPC: −1.22, 4); however, Vietnam faced rising trends due to unregulated industrial pollution (20). The observed trends underscore the dual role of industrialization and public health interventions in shaping the burden of pneumoconiosis. Sustained declines in mortality are achievable through continued vigilance in occupational safety and equitable healthcare access, but rising incidence demands urgent action to address systemic gaps in worker protection, particularly in LMICs and emerging industries. The divergence between rising pneumoconiosis incidence and declining mortality underscores the dual challenge of addressing persistent occupational hazards while leveraging medical advancements to improve outcomes. Eliminating pneumoconiosis as a public health threat demands a harmonized focus on prevention (reducing exposures) and care (extending survival), particularly in regions and industries where risks remain unmitigated.

### Differences between regions

Regional disparities in the burden of pneumoconiosis remain pronounced. East Asia accounted for nearly half of the global incident cases (30,658 in 2021), reflecting its prominence in mining and manufacturing (25). In contrast, Australasia experienced the most rapid increase in age-standardized incidence rates (ASIR) with an estimated annual percentage change (EAPC) of 2.99, attributed to unregulated artificial stone processing (4). High-income regions, such as Western Europe, demonstrated significant declines in age-standardized death rates (ASDR) with an EAPC of −2.38, underscoring the effectiveness of occupational regulations (14). Conversely, low-and middle-income countries (LMICs) in Sub-Saharan Africa and South Asia are confronting rising burdens due to inadequate dust control measures and underreporting (26). For instance, Southern Sub-Saharan Africa recorded the highest ASDR (0.48 per 100,000), while countries with low socio-demographic indices (SDI) exhibited elevated mortality rates despite lower incidence figures, indicating systemic deficiencies in healthcare (16). Chile and South Africa have been consistently recognized as high-burden nations for pneumoconiosis-related mortality in mining populations, based on recent mortality trend analyses (4), while Turkey and Vietnam are experiencing increasing burdens of silica-associated lung cancer. These disparities highlight the complex interplay between industrialization, regulatory frameworks, and socioeconomic development (22).

The lack of a significant correlation between the burden of pneumoconiosis and the SDI may be attributed to the complex interplay of occupational exposure, regulatory frameworks, and healthcare access, which are not fully captured by the SDI. Specifically, the burden of pneumoconiosis is more directly influenced by occupational safety measures, industrial practices, and dust exposure levels, rather than broader socioeconomic development indicators such as income, education, and fertility, which are encapsulated in the SDI.

### Pneumoconiosis control

Current control strategies remain inadequate, particularly in low-and middle-income countries (LMICs). Although high-resolution computed tomography (CT) and antifibrotic therapies such as pirfenidone provide diagnostic and palliative benefits (15), primary prevention through dust suppression and occupational health surveillance is essential. Developed nations have successfully reduced incidence rates through the enforcement of safety standards; however, the resurgence of silicosis associated with the artificial stone industry underscores existing regulatory gaps (4, 7). In LMICs, informal labor sectors frequently lack access to protective equipment and compensation systems, thereby perpetuating health inequities (27). Delayed diagnoses—often occurring decades after exposure—limit the efficacy of therapeutic interventions, as patients diagnosed at advanced stages typically face median survival times of only 6 to 9 years. A 18.6% comorbidity rate of chronic obstructive pulmonary disease (COPD) has been documented among pneumoconiosis patients, with disease severity escalating in advanced cases (28), which emphasizes the necessity for comprehensive screening initiatives.

### Suggestions for strategies

A multi-pronged approach is essential to mitigate the global burden of pneumoconiosis. First, low-and middle-income countries (LMICs) must prioritize occupational safety legislation and enforce compliance in high-risk industries (26). Second, integrating pneumoconiosis screening into primary healthcare systems could facilitate early detection, particularly for aging workers with prolonged exposure to dust (21). Third, public-private partnerships should incentivize innovations in dust-control technologies, such as wet drilling and ventilation systems, especially in emerging industries (4). Additionally, global initiatives must address socioeconomic determinants, including the improvement of education and healthcare access for vulnerable populations (17). Recent analyses reveal an urgent requirement for enhanced coal dust regulation in LMICs, as coal worker’s pneumoconiosis (CWP) incidence remains disproportionately high in these regions (25), necessitating strengthened regulatory measures and comprehensive worker compensation systems.

### Healthcare task-shifting

Task-shifting—delegating screening and education to community health workers—has the potential to enhance resource efficiency in low-and middle-income countries (LMICs). Training non-specialists to administer spirometry or symptom questionnaires may improve early diagnosis rates in underserved regions (5). Mobile health units that deliver occupational safety training to remote mining communities could help reduce exposure risks (29). However, these strategies necessitate robust funding and integration with national health systems to ensure sustainability. Registry data from China’s National Occupational Disease Monitoring System indicate a progressive annual decline in silicosis incidence (AAPC −4.7, 95%CI −5.2 to −4.1) since 2010, proving the scalability of integrated surveillance models for resource-limited settings (2).

### Limitations of the study

This study has several limitations. First, reliance on Global Burden of Disease (GBD) data may underestimate the true prevalence of pneumoconiosis due to underreporting in low-and middle-income countries (LMICs) and informal sectors. Second, age-specific analyses excluded individuals under 15 years, potentially omitting cases of juvenile labor in regions with lax child labor laws (30). Third, the ecological nature of the correlations with the Socio-Demographic Index (SDI) limits causal inferences regarding socioeconomic determinants (31). Finally, emerging risk factors, such as mixed-dust exposures in non-traditional industries, were not fully captured in the GBD 2021 framework (25). The lack of a significant correlation between pneumoconiosis burden and SDI underscores the need for a more nuanced understanding of the disease’s determinants. While SDI provides a useful framework for assessing broader socioeconomic development, it may not fully capture the specific occupational and regulatory factors that drive pneumoconiosis. Future research should focus on occupational exposure levels, regulatory enforcement, and access to occupational health services to better understand and mitigate the global burden of pneumoconiosis.

### COVID-19 and pneumoconiosis: synergistic impacts and epidemiological shifts

The COVID-19 pandemic has significantly influenced the global burden of pneumoconiosis through both direct and indirect pathways, exacerbating existing challenges in occupational health systems and patient outcomes. Epidemiological implications include:

Accelerated fibrosis: COVID-19-induced alveolar injury and cytokine storms (e.g., IL-6, TNF-*α*) may synergize with silica/coal dust to accelerate pulmonary fibrosis. Rapid radiographic progression has been reported in cases of pneumoconiosis complicated by COVID-19, suggesting potential exacerbation of fibrotic pathways by viral infection (32).

Diagnostic and surveillance gaps: pandemic-related disruptions delayed routine pneumoconiosis screenings in LMICs. Surveillance data from Hubei Province revealed a 37% decrease in occupational health monitoring during the pandemic years 2020–2021 (33), creating diagnostic gaps for incipient pneumoconiosis and impairing coordinated COVID-19 prevention efforts.

Compounded health inequities: informal sector workers in LMICs faced dual barriers: inadequate PPE against dust and limited access to COVID-19 vaccines. A predominance of male miners (90.5%) with cumulative dust exposure exceeding permissible limits was identified in pneumoconiosis-COVID-19 cohorts, demonstrating occupational health system failures in high-risk sectors (32).

## Conclusion

The ongoing challenge posed by pneumoconiosis highlights the critical necessity for global collaboration to tackle occupational health disparities. By emphasizing prevention, enhancing healthcare systems, and adapting policies to the changing industrial landscape, the international community can make significant strides toward the 2030 objective of eradicating pneumoconiosis.

## Data Availability

The original contributions presented in the study are included in the article/Supplementary material, further inquiries can be directed to the corresponding author.
