# Supplementary material for: Global burden of pneumoconiosis from 1990 to 2021: a comprehensive analysis of incidence, mortality, and socio-demographic inequalities in 204 countries and territories
Source: Front Public Health. 2025 Apr 23;13:1579851. doi: 10.3389/fpubh.2025.1579851 (PMC12055836; doi:10.3389/fpubh.2025.1579851)
Supplement: Supplementary file 1 [file Table_1.docx]

**Additional file 1**

**Global Burden of Pneumoconiosis from 1990 to 2021: A Comprehensive Analysis of Incidence, Mortality, and Socio-Demographic Inequalities in 204 Countries and Territories**

Author: Shenyu Zhang^a,1^ Jun Xiong^a,2*^ Xinyi Ruan^a,3^ Chongyan Ji^a,4^ Hanxin Lu^a,5^

^a^ School of Public Health, Hangzhou Normal University, Hangzhou, 311121, Zhejiang, China

^*^Corresponding Author: Jun Xiong, [20040044@hznu.edu.cn](mailto:20040044@hznu.edu.cn)

**Table S1** The ASIR, ASDR, and age-standardized DALYs rate of Pneumoconiosis in 204 countries or territories in 2021 (Related to Figure 1).

|  | **ASIR / 100,000**  **(95%UI)** | **ASDR / 100,000**  **(95%UI)** | **AS-DALYs Rate / 100,000**  **(95%UI)** |
| --- | --- | --- | --- |
| Afghanistan | 0.31(0.26,0.39) | 0.06(0.03,0.12) | 1.48(0.85,2.70) |
| Albania | 0.59(0.52,0.68) | 0.21(0.11,0.39) | 4.67(2.66,7.98) |
| Algeria | 0.25(0.20,0.31) | 0.04(0.03,0.06) | 0.97(0.71,1.44) |
| American Samoa | 0.39(0.30,0.49) | 0.03(0.01,0.05) | 0.77(0.48,1.15) |
| Andorra | 0.65(0.57,0.74) | 0.16(0.09,0.26) | 3.01(1.78,4.63) |
| Angola | 0.35(0.31,0.40) | 0.27(0.15,0.46) | 5.94(3.32,9.73) |
| Antigua and Barbuda | 0.19(0.15,0.23) | 0.00(0.00,0.00) | 0.17(0.11,0.25) |
| Argentina | 0.25(0.20,0.30) | 0.05(0.04,0.06) | 1.24(1.06,1.42) |
| Armenia | 0.29(0.23,0.35) | 0.03(0.03,0.04) | 1.09(0.92,1.31) |
| Australia | 0.52(0.48,0.58) | 0.32(0.27,0.37) | 5.20(4.49,5.98) |
| Austria | 0.89(0.75,1.02) | 0.07(0.06,0.08) | 2.29(1.88,2.76) |
| Azerbaijan | 0.27(0.20,0.33) | 0.03(0.02,0.06) | 0.99(0.62,1.67) |
| Bahrain | 0.36(0.30,0.43) | 0.15(0.12,0.19) | 3.33(2.65,4.10) |
| Bangladesh | 0.41(0.35,0.49) | 0.10(0.05,0.16) | 2.20(1.29,3.49) |
| Barbados | 0.16(0.12,0.21) | 0.01(0.00,0.01) | 0.26(0.20,0.34) |
| Belarus | 0.34(0.25,0.45) | 0.02(0.02,0.03) | 0.79(0.61,1.03) |
| Belgium | 0.70(0.62,0.77) | 0.33(0.27,0.38) | 5.88(5.03,6.73) |
| Belize | 0.30(0.26,0.35) | 0.12(0.10,0.15) | 3.02(2.55,3.68) |
| Benin | 0.13(0.10,0.17) | 0.41(0.18,0.70) | 8.44(3.89,14.42) |
| Bermuda | 0.16(0.12,0.20) | 0.00(0.00,0.00) | 0.27(0.20,0.36) |
| Bhutan | 0.47(0.40,0.55) | 0.11(0.06,0.18) | 2.21(1.36,3.51) |
| Bolivarian Republic of Venezuela | 0.58(0.46,0.74) | 0.04(0.03,0.05) | 1.44(1.10,1.84) |
| Bosnia and Herzegovina | 0.45(0.36,0.55) | 0.08(0.05,0.12) | 2.03(1.43,2.92) |
| Botswana | 0.60(0.54,0.67) | 0.63(0.42,0.97) | 13.56(9.08,20.78) |
| Brazil | 0.49(0.43,0.56) | 0.16(0.15,0.18) | 4.53(4.21,4.91) |
| Brunei Darussalam | 0.53(0.40,0.70) | 0.01(0.01,0.02) | 0.64(0.46,0.89) |
| Bulgaria | 0.52(0.45,0.60) | 0.17(0.11,0.24) | 4.02(2.86,5.52) |
| Burkina Faso | 0.13(0.10,0.17) | 0.30(0.14,0.59) | 6.19(2.88,11.94) |
| Burundi | 0.51(0.45,0.58) | 0.37(0.15,0.69) | 7.85(3.39,14.03) |
| Cambodia | 0.22(0.17,0.29) | 0.01(0.01,0.02) | 0.46(0.32,0.67) |
| Cameroon | 0.13(0.10,0.17) | 0.46(0.24,0.74) | 9.85(5.32,15.78) |
| Canada | 0.60(0.51,0.72) | 0.11(0.09,0.13) | 2.28(1.93,2.68) |
| Central African Republic | 0.62(0.55,0.70) | 0.57(0.21,1.23) | 13.35(5.29,26.11) |
| Chad | 0.15(0.12,0.20) | 0.57(0.28,1.03) | 11.82(5.88,21.35) |
| Chile | 1.03(0.96,1.11) | 0.44(0.37,0.51) | 8.84(7.66,10.15) |
| China | 1.42(1.21,1.63) | 0.41(0.32,0.53) | 10.86(8.49,13.87) |
| Colombia | 0.65(0.55,0.76) | 0.16(0.13,0.19) | 4.05(3.40,4.77) |
| Commonwealth of the Bahamas | 0.20(0.16,0.24) | 0.03(0.02,0.04) | 0.89(0.69,1.09) |
| Comoros | 0.42(0.37,0.47) | 0.30(0.14,0.53) | 6.18(3.14,11.15) |
| Congo | 0.38(0.33,0.43) | 0.37(0.17,0.59) | 7.92(3.73,12.30) |
| Cook Islands | 0.36(0.30,0.43) | 0.02(0.01,0.04) | 0.61(0.39,0.92) |
| Costa Rica | 0.59(0.45,0.75) | 0.03(0.03,0.04) | 1.48(1.18,1.88) |
| Croatia | 0.50(0.44,0.59) | 0.06(0.05,0.08) | 1.76(1.38,2.18) |
| Cuba | 0.15(0.11,0.20) | 0.00(0.00,0.00) | 0.19(0.13,0.27) |
| Cyprus | 0.40(0.33,0.50) | 0.09(0.06,0.13) | 1.84(1.36,2.44) |
| Czech Republic | 0.58(0.50,0.67) | 0.17(0.13,0.22) | 4.10(3.13,5.26) |
| Democratic People's Republic of Korea | 1.34(1.18,1.51) | 0.30(0.17,0.48) | 9.20(5.45,14.30) |
| Democratic Republic of the Congo | 0.45(0.40,0.52) | 0.51(0.17,1.27) | 10.93(4.06,24.06) |
| Denmark | 0.28(0.24,0.32) | 0.10(0.08,0.11) | 1.84(1.61,2.08) |
| Djibouti | 0.44(0.38,0.50) | 0.27(0.15,0.46) | 5.90(3.32,10.28) |
| Dominica | 0.17(0.13,0.22) | 0.01(0.01,0.01) | 0.34(0.27,0.43) |
| Dominican Republic | 0.17(0.13,0.22) | 0.02(0.01,0.03) | 0.65(0.49,0.89) |
| Ecuador | 0.34(0.29,0.41) | 0.05(0.04,0.07) | 1.43(1.15,1.75) |
| Egypt | 0.27(0.22,0.34) | 0.48(0.34,0.67) | 11.06(8.05,15.07) |
| El Salvador | 0.63(0.49,0.80) | 0.00(0.00,0.01) | 0.84(0.59,1.19) |
| Equatorial Guinea | 0.37(0.32,0.42) | 0.24(0.13,0.43) | 5.14(2.90,9.19) |
| Eritrea | 0.40(0.35,0.46) | 0.31(0.16,0.60) | 6.96(3.71,13.37) |
| Estonia | 0.33(0.24,0.43) | 0.01(0.01,0.02) | 0.62(0.48,0.81) |
| Ethiopia | 0.39(0.33,0.44) | 0.23(0.10,0.42) | 4.73(2.27,8.77) |
| Federated States of Micronesia | 0.58(0.49,0.68) | 0.04(0.02,0.09) | 1.09(0.67,1.76) |
| Fiji | 0.39(0.30,0.50) | 0.00(0.00,0.01) | 0.37(0.24,0.52) |
| Finland | 0.70(0.59,0.81) | 0.16(0.14,0.19) | 3.47(3.00,3.93) |
| France | 0.69(0.62,0.76) | 0.25(0.21,0.29) | 4.36(3.78,5.08) |
| Gabon | 0.35(0.30,0.40) | 0.27(0.15,0.47) | 5.97(3.53,10.28) |
| Georgia | 0.38(0.32,0.45) | 0.06(0.04,0.07) | 1.65(1.28,2.10) |
| Germany | 0.49(0.44,0.56) | 0.18(0.15,0.21) | 3.31(2.85,3.77) |
| Ghana | 0.12(0.09,0.16) | 0.39(0.22,0.59) | 7.93(4.76,12.45) |
| Greece | 0.30(0.21,0.39) | 0.00(0.00,0.00) | 0.32(0.22,0.42) |
| Greenland | 0.62(0.50,0.76) | 0.03(0.02,0.04) | 1.03(0.75,1.38) |
| Grenada | 0.23(0.19,0.28) | 0.07(0.06,0.08) | 1.83(1.52,2.16) |
| Guam | 0.34(0.26,0.43) | 0.00(0.00,0.01) | 0.41(0.27,0.58) |
| Guatemala | 0.73(0.55,0.92) | 0.07(0.05,0.08) | 2.17(1.80,2.64) |
| Guinea | 0.14(0.10,0.18) | 0.53(0.27,0.86) | 11.07(5.87,18.09) |
| Guinea-Bissau | 0.16(0.13,0.21) | 0.62(0.29,1.09) | 13.62(6.48,24.33) |
| Guyana | 0.21(0.16,0.27) | 0.02(0.02,0.03) | 0.67(0.52,0.85) |
| Haiti | 0.28(0.23,0.33) | 0.04(0.02,0.08) | 1.01(0.50,1.81) |
| Honduras | 0.67(0.55,0.80) | 0.14(0.09,0.20) | 3.64(2.67,4.78) |
| Hungary | 0.46(0.40,0.54) | 0.10(0.07,0.13) | 2.53(1.98,3.26) |
| Iceland | 0.39(0.30,0.49) | 0.02(0.02,0.02) | 0.76(0.60,0.95) |
| India | 0.50(0.44,0.58) | 0.16(0.10,0.22) | 3.36(2.30,4.57) |
| Indonesia | 0.44(0.35,0.55) | 0.01(0.01,0.02) | 0.79(0.59,1.07) |
| Iraq | 0.34(0.29,0.41) | 0.12(0.08,0.17) | 2.97(2.00,4.27) |
| Ireland | 0.37(0.30,0.44) | 0.03(0.03,0.04) | 0.92(0.76,1.11) |
| Islamic Republic of Iran | 0.33(0.28,0.38) | 0.06(0.05,0.07) | 1.55(1.30,1.78) |
| Israel | 0.43(0.32,0.54) | 0.02(0.02,0.02) | 0.70(0.55,0.90) |
| Italy | 0.50(0.43,0.56) | 0.17(0.15,0.19) | 2.85(2.54,3.13) |
| Jamaica | 0.16(0.12,0.21) | 0.10(0.08,0.13) | 2.31(1.74,3.03) |
| Japan | 0.56(0.49,0.65) | 0.17(0.15,0.19) | 3.14(2.82,3.42) |
| Jordan | 0.13(0.09,0.16) | 0.03(0.02,0.06) | 0.79(0.55,1.31) |
| Kazakhstan | 0.71(0.63,0.78) | 0.41(0.26,0.62) | 9.87(6.63,14.39) |
| Kenya | 0.40(0.34,0.45) | 0.35(0.14,0.78) | 7.35(3.20,14.34) |
| Kingdom of Eswatini | 0.78(0.70,0.86) | 1.52(0.76,2.53) | 35.53(17.63,57.85) |
| Kiribati | 1.42(1.12,1.76) | 0.05(0.02,0.13) | 1.60(0.99,2.73) |
| Kuwait | 0.21(0.17,0.28) | 0.01(0.01,0.01) | 0.40(0.30,0.55) |
| Kyrgyzstan | 0.29(0.22,0.37) | 0.05(0.03,0.10) | 1.45(1.00,2.51) |
| Lao People's Democratic Republic | 0.24(0.18,0.30) | 0.01(0.01,0.03) | 0.50(0.33,0.76) |
| Latvia | 0.35(0.26,0.45) | 0.02(0.02,0.03) | 0.86(0.67,1.11) |
| Lebanon | 0.22(0.17,0.28) | 0.14(0.10,0.19) | 3.19(2.43,4.01) |
| Lesotho | 1.20(1.08,1.34) | 1.08(0.40,1.92) | 25.76(10.10,46.86) |
| Liberia | 0.13(0.10,0.17) | 0.50(0.20,0.98) | 10.12(4.07,19.76) |
| Libya | 0.23(0.19,0.29) | 0.04(0.02,0.09) | 1.21(0.76,2.21) |
| Lithuania | 0.30(0.23,0.39) | 0.02(0.01,0.02) | 0.62(0.49,0.78) |
| Luxembourg | 0.63(0.51,0.78) | 0.08(0.06,0.09) | 2.09(1.77,2.51) |
| Madagascar | 0.50(0.44,0.57) | 0.52(0.22,1.07) | 10.52(4.53,21.14) |
| Malawi | 0.45(0.39,0.51) | 0.37(0.16,0.67) | 8.11(3.81,14.78) |
| Malaysia | 0.20(0.15,0.26) | 0.00(0.00,0.01) | 0.32(0.23,0.42) |
| Maldives | 0.21(0.17,0.26) | 0.07(0.05,0.10) | 1.58(1.13,2.23) |
| Mali | 0.14(0.10,0.18) | 0.81(0.42,1.40) | 16.99(9.01,29.51) |
| Malta | 0.73(0.55,0.95) | 0.06(0.05,0.07) | 1.81(1.47,2.25) |
| Marshall Islands | 0.62(0.51,0.72) | 0.03(0.01,0.06) | 1.03(0.65,1.51) |
| Mauritania | 0.11(0.08,0.15) | 0.35(0.20,0.58) | 6.87(3.92,11.13) |
| Mauritius | 0.21(0.16,0.27) | 0.01(0.01,0.01) | 0.46(0.38,0.57) |
| Mexico | 0.69(0.58,0.80) | 0.08(0.07,0.10) | 2.67(2.27,3.16) |
| Mongolia | 0.41(0.34,0.48) | 0.19(0.12,0.29) | 4.86(3.25,6.93) |
| Montenegro | 0.48(0.40,0.57) | 0.12(0.07,0.21) | 2.69(1.78,4.17) |
| Morocco | 0.25(0.20,0.31) | 0.04(0.02,0.07) | 1.02(0.72,1.65) |
| Mozambique | 0.39(0.34,0.44) | 0.32(0.14,0.65) | 7.41(3.58,15.37) |
| Myanmar | 0.24(0.19,0.31) | 0.02(0.01,0.03) | 0.64(0.42,0.93) |
| Namibia | 0.63(0.56,0.70) | 0.65(0.40,0.94) | 14.18(8.97,20.23) |
| Nepal | 0.95(0.85,1.07) | 0.19(0.11,0.30) | 4.46(2.71,6.60) |
| Netherlands | 0.37(0.31,0.45) | 0.09(0.08,0.11) | 1.87(1.62,2.16) |
| New Zealand | 0.47(0.39,0.54) | 0.25(0.21,0.30) | 4.22(3.64,4.95) |
| Nicaragua | 0.55(0.43,0.70) | 0.05(0.04,0.06) | 1.76(1.42,2.15) |
| Niger | 0.13(0.10,0.17) | 0.49(0.20,0.87) | 9.47(3.99,17.97) |
| Nigeria | 0.12(0.09,0.15) | 0.32(0.18,0.51) | 6.10(3.38,9.73) |
| Northern Mariana Islands | 0.58(0.48,0.68) | 0.09(0.02,0.16) | 1.66(0.74,2.67) |
| Norway | 0.35(0.29,0.42) | 0.07(0.06,0.08) | 1.52(1.35,1.70) |
| Oman | 0.28(0.21,0.36) | 0.00(0.00,0.01) | 0.36(0.24,0.54) |
| Pakistan | 0.48(0.41,0.55) | 0.11(0.07,0.17) | 2.49(1.68,3.56) |
| Palestine | 0.23(0.17,0.30) | 0.13(0.07,0.19) | 2.63(1.69,3.76) |
| Panama | 0.62(0.48,0.78) | 0.02(0.01,0.02) | 1.20(0.87,1.63) |
| Papua New Guinea | 0.73(0.62,0.85) | 0.08(0.02,0.18) | 1.85(0.97,3.75) |
| Paraguay | 0.63(0.55,0.72) | 0.24(0.17,0.32) | 6.82(5.04,8.90) |
| Peru | 0.44(0.39,0.50) | 0.28(0.19,0.41) | 6.22(4.37,8.84) |
| Philippines | 0.10(0.07,0.13) | 0.01(0.01,0.01) | 0.27(0.22,0.35) |
| Plurinational State of Bolivia | 0.51(0.45,0.57) | 0.18(0.12,0.25) | 4.00(2.91,5.44) |
| Poland | 0.62(0.52,0.73) | 0.06(0.05,0.07) | 2.10(1.76,2.50) |
| Portugal | 0.61(0.55,0.67) | 0.28(0.24,0.32) | 5.57(4.89,6.33) |
| Principality of Monaco | 0.84(0.74,0.93) | 0.10(0.07,0.15) | 2.36(1.78,3.09) |
| Puerto Rico | 0.16(0.12,0.21) | 0.01(0.01,0.01) | 0.43(0.35,0.53) |
| Qatar | 0.27(0.21,0.35) | 0.06(0.03,0.10) | 1.41(0.83,2.21) |
| Republic of Cabo Verde | 0.11(0.09,0.15) | 0.30(0.19,0.48) | 6.31(4.11,9.88) |
| Republic of Côte d'Ivoire | 0.13(0.10,0.17) | 0.44(0.21,0.73) | 9.40(4.59,15.60) |
| Republic of Korea | 0.80(0.74,0.87) | 0.41(0.31,0.53) | 7.58(5.81,9.65) |
| Republic of Moldova | 0.36(0.26,0.48) | 0.00(0.00,0.00) | 0.42(0.30,0.59) |
| Republic of Nauru | 0.55(0.46,0.63) | 0.04(0.01,0.08) | 0.98(0.60,1.69) |
| Republic of Niue | 0.43(0.35,0.50) | 0.03(0.01,0.06) | 0.77(0.50,1.18) |
| Republic of Palau | 0.51(0.42,0.59) | 0.02(0.01,0.04) | 0.81(0.54,1.15) |
| Republic of San Marino | 0.45(0.38,0.52) | 0.14(0.09,0.22) | 2.82(1.85,4.28) |
| Republic of the Gambia | 0.14(0.11,0.18) | 0.54(0.25,0.93) | 11.49(5.61,19.43) |
| Romania | 0.39(0.31,0.47) | 0.09(0.07,0.11) | 2.33(1.90,2.85) |
| Russian Federation | 0.28(0.23,0.33) | 0.06(0.05,0.06) | 1.42(1.28,1.57) |
| Rwanda | 0.37(0.32,0.42) | 0.32(0.15,0.54) | 6.85(3.32,11.31) |
| Saint Kitts and Nevis | 0.18(0.14,0.23) | 0.03(0.02,0.04) | 0.77(0.61,1.02) |
| Saint Lucia | 0.19(0.15,0.23) | 0.01(0.01,0.01) | 0.39(0.32,0.50) |
| Saint Vincent and the Grenadines | 0.23(0.19,0.28) | 0.00(0.00,0.00) | 0.25(0.18,0.34) |
| Samoa | 0.49(0.41,0.57) | 0.02(0.01,0.05) | 0.79(0.49,1.27) |
| Sao Tome and Principe | 0.17(0.14,0.21) | 1.09(0.64,1.91) | 22.09(13.02,39.12) |
| Saudi Arabia | 0.26(0.20,0.34) | 0.01(0.01,0.01) | 0.52(0.40,0.68) |
| Senegal | 0.12(0.09,0.16) | 0.44(0.23,0.74) | 8.84(4.92,15.05) |
| Serbia | 0.21(0.16,0.26) | 0.03(0.02,0.04) | 0.84(0.64,1.08) |
| Seychelles | 0.20(0.16,0.26) | 0.01(0.00,0.01) | 0.33(0.23,0.49) |
| Sierra Leone | 0.14(0.10,0.18) | 0.48(0.20,0.86) | 9.91(4.44,18.09) |
| Singapore | 0.54(0.40,0.68) | 0.01(0.01,0.01) | 0.61(0.44,0.82) |
| Slovakia | 0.61(0.49,0.80) | 0.08(0.06,0.12) | 2.50(1.91,3.28) |
| Slovenia | 0.44(0.38,0.50) | 0.07(0.06,0.09) | 1.82(1.48,2.23) |
| Socialist Republic of Viet Nam | 0.31(0.24,0.38) | 0.01(0.01,0.02) | 0.62(0.44,0.88) |
| Solomon Islands | 0.60(0.51,0.68) | 0.03(0.01,0.07) | 0.91(0.57,1.54) |
| Somalia | 0.83(0.71,0.96) | 0.53(0.17,1.23) | 12.33(4.24,28.41) |
| South Africa | 0.71(0.62,0.81) | 0.48(0.41,0.58) | 10.66(9.19,12.81) |
| South Sudan | 0.50(0.44,0.56) | 0.45(0.19,0.84) | 9.72(4.28,17.60) |
| Spain | 0.43(0.39,0.48) | 0.17(0.15,0.20) | 3.02(2.65,3.40) |
| Sri Lanka | 0.19(0.14,0.25) | 0.01(0.01,0.02) | 0.50(0.36,0.69) |
| Sudan | 0.27(0.21,0.33) | 0.04(0.02,0.07) | 1.20(0.72,1.90) |
| Suriname | 0.31(0.27,0.36) | 0.07(0.05,0.11) | 2.01(1.46,2.71) |
| Sweden | 0.24(0.20,0.28) | 0.05(0.04,0.06) | 0.92(0.77,1.09) |
| Switzerland | 0.31(0.25,0.38) | 0.07(0.06,0.08) | 1.50(1.29,1.70) |
| Syrian Arab Republic | 0.23(0.18,0.29) | 0.05(0.03,0.08) | 1.31(0.88,2.02) |
| Taiwan (Province of China) | 1.21(1.07,1.35) | 0.44(0.38,0.51) | 10.59(9.38,11.84) |
| Tajikistan | 0.28(0.20,0.36) | 0.03(0.01,0.05) | 0.79(0.48,1.30) |
| Thailand | 0.19(0.15,0.25) | 0.01(0.01,0.01) | 0.47(0.36,0.60) |
| The former Yugoslav Republic of Macedonia | 0.41(0.32,0.52) | 0.09(0.06,0.12) | 2.19(1.59,2.95) |
| Timor-Leste | 0.22(0.17,0.28) | 0.01(0.01,0.03) | 0.51(0.35,0.76) |
| Togo | 0.14(0.10,0.18) | 0.53(0.23,0.97) | 11.59(4.91,20.66) |
| Tokelau | 0.42(0.35,0.50) | 0.02(0.01,0.05) | 0.75(0.49,1.14) |
| Tonga | 0.47(0.39,0.55) | 0.03(0.01,0.06) | 0.82(0.55,1.29) |
| Trinidad and Tobago | 0.17(0.13,0.22) | 0.01(0.01,0.01) | 0.41(0.33,0.51) |
| Tunisia | 0.22(0.18,0.27) | 0.04(0.02,0.06) | 0.98(0.67,1.53) |
| Turkey | 0.52(0.41,0.65) | 0.07(0.05,0.10) | 2.29(1.81,2.86) |
| Turkmenistan | 0.26(0.20,0.34) | 0.02(0.01,0.04) | 0.82(0.57,1.23) |
| Tuvalu | 0.52(0.43,0.61) | 0.03(0.01,0.07) | 0.90(0.56,1.47) |
| Uganda | 0.40(0.35,0.45) | 0.33(0.13,0.56) | 7.06(3.05,11.98) |
| Ukraine | 0.32(0.26,0.38) | 0.04(0.03,0.06) | 1.28(0.92,1.71) |
| United Arab Emirates | 0.30(0.25,0.37) | 0.05(0.03,0.09) | 1.39(0.98,1.94) |
| United Kingdom of Great Britain and Northern Ireland | 0.85(0.74,0.98) | 0.27(0.25,0.29) | 4.80(4.43,5.20) |
| United Republic of Tanzania | 0.31(0.27,0.35) | 0.22(0.10,0.37) | 4.66(2.25,7.61) |
| United States of America | 0.70(0.60,0.81) | 0.12(0.10,0.13) | 2.57(2.30,2.90) |
| United States Virgin Islands | 0.21(0.17,0.25) | 0.02(0.02,0.03) | 0.69(0.52,0.90) |
| Uruguay | 0.38(0.29,0.48) | 0.03(0.03,0.04) | 0.99(0.82,1.20) |
| Uzbekistan | 0.29(0.24,0.35) | 0.05(0.03,0.06) | 1.33(1.02,1.70) |
| Vanuatu | 0.56(0.47,0.65) | 0.01(0.00,0.02) | 0.58(0.37,0.91) |
| Yemen | 0.29(0.23,0.35) | 0.05(0.03,0.08) | 1.27(0.81,1.93) |
| Zambia | 0.39(0.34,0.44) | 0.37(0.19,0.58) | 8.23(4.44,12.82) |
| Zimbabwe | 0.41(0.33,0.51) | 0.26(0.18,0.36) | 5.96(4.17,8.13) |

Notes: ASIR/100,000: age-standardized incidence rate (per 100,000 population); ASDR/100,000: age-standardized death rate (per 100,000 population); AS-DALYs rate/100,000: age-standardized Disability-Adjusted Life Years rate (per 100,000 population).

**Table S2.** The EAPCs of incidence rate, mortality rate, and DALYs rate caused by Pneumoconiosis in 204 countries or territories from 1990 to 2021 (Related to **Figure 2**).

|  | **EAPCs of incidence rate(95%CI)** | **EAPCs of mortality rate(95%CI)** | **EAPCs of DALYs rate(95%CI)** |
| --- | --- | --- | --- |
| Afghanistan | -0.24(-0.29,-0.19) | 2.47(2.20,2.74) | 1.97(1.63,2.31) |
| Albania | -2.74(-3.16,-2.31) | -2.87(-3.14,-2.60) | -3.20(-3.57,-2.83) |
| Algeria | -0.13(-0.19,-0.08) | 2.44(2.05,2.82) | 1.92(1.71,2.13) |
| American Samoa | 0.01(-0.25,0.26) | 2.08(1.85,2.30) | 1.24(1.04,1.45) |
| Andorra | -0.80(-0.91,-0.70) | -2.04(-2.30,-1.78) | -2.59(-2.92,-2.26) |
| Angola | -1.68(-1.75,-1.62) | -1.64(-1.78,-1.50) | -2.20(-2.32,-2.08) |
| Antigua and Barbuda | -1.53(-1.60,-1.47) | -4.04(-4.99,-3.08) | -1.53(-1.64,-1.42) |
| Argentina | -0.01(-0.41,0.39) | -0.83(-1.15,-0.52) | -1.59(-1.86,-1.32) |
| Armenia | 0.20(-0.01,0.41) | -3.54(-3.86,-3.23) | -3.88(-4.17,-3.59) |
| Australia | 1.61(1.38,1.84) | 1.56(1.21,1.91) | 1.53(1.11,1.95) |
| Austria | 0.03(-0.72,0.79) | -3.23(-3.43,-3.02) | -1.78(-1.98,-1.57) |
| Azerbaijan | -0.40(-0.58,-0.21) | 1.67(1.21,2.12) | -0.05(-0.49,0.38) |
| Bahrain | 0.68(0.59,0.78) | 2.00(1.43,2.56) | 2.23(1.49,2.99) |
| Bangladesh | -1.57(-1.62,-1.51) | -1.96(-2.22,-1.69) | -2.65(-2.88,-2.43) |
| Barbados | -0.50(-0.63,-0.36) | -1.73(-2.13,-1.33) | -1.46(-1.69,-1.23) |
| Belarus | -0.39(-0.52,-0.26) | -6.92(-7.76,-6.07) | -6.72(-7.29,-6.14) |
| Belgium | -4.41(-4.57,-4.24) | -6.71(-6.91,-6.50) | -7.69(-7.88,-7.50) |
| Belize | -0.54(-0.67,-0.40) | -1.59(-2.24,-0.94) | -2.48(-3.37,-1.59) |
| Benin | -0.39(-0.42,-0.35) | -2.32(-2.45,-2.19) | -2.23(-2.46,-1.99) |
| Bermuda | -1.95(-2.01,-1.90) | -8.41(-9.58,-7.22) | -8.27(-8.94,-7.59) |
| Bhutan | -0.37(-0.40,-0.33) | -0.71(-0.75,-0.67) | -1.02(-1.08,-0.96) |
| Bolivarian Republic of Venezuela | -0.64(-0.79,-0.49) | -2.15(-2.46,-1.85) | -1.53(-1.78,-1.29) |
| Bosnia and Herzegovina | -0.38(-0.56,-0.19) | -2.31(-2.43,-2.20) | -2.01(-2.15,-1.88) |
| Botswana | -1.75(-1.94,-1.56) | -1.47(-1.66,-1.28) | -1.91(-2.27,-1.55) |
| Brazil | -1.14(-1.33,-0.95) | 0.35(-0.05,0.74) | -0.88(-1.41,-0.34) |
| Brunei Darussalam | -0.72(-1.04,-0.40) | 0.72(0.22,1.23) | -0.63(-0.84,-0.42) |
| Bulgaria | -3.23(-3.32,-3.14) | -4.51(-4.86,-4.17) | -5.11(-5.47,-4.74) |
| Burkina Faso | -0.44(-0.53,-0.35) | -1.47(-1.54,-1.39) | -1.58(-1.73,-1.43) |
| Burundi | -0.77(-0.80,-0.74) | -1.72(-1.88,-1.56) | -2.05(-2.33,-1.78) |
| Cambodia | -0.46(-0.60,-0.33) | 0.39(0.27,0.51) | -0.19(-0.30,-0.08) |
| Cameroon | -0.35(-0.49,-0.20) | -1.75(-1.82,-1.68) | -1.78(-1.87,-1.69) |
| Canada | 0.20(0.13,0.27) | -2.05(-2.12,-1.99) | -1.96(-2.03,-1.89) |
| Central African Republic | -0.59(-0.63,-0.56) | -0.47(-0.55,-0.39) | -0.75(-0.83,-0.67) |
| Chad | -0.09(-0.13,-0.04) | -1.12(-1.17,-1.07) | -1.02(-1.07,-0.97) |
| Chile | -2.48(-2.65,-2.31) | -2.59(-2.80,-2.39) | -3.26(-3.46,-3.06) |
| China | -1.52(-1.60,-1.44) | -2.22(-2.33,-2.10) | -2.62(-2.69,-2.55) |
| Colombia | -0.95(-1.05,-0.85) | -3.22(-3.64,-2.80) | -3.00(-3.50,-2.50) |
| Commonwealth of the Bahamas | -0.62(-0.67,-0.58) | -1.81(-2.26,-1.35) | -2.30(-2.89,-1.70) |
| Comoros | -0.75(-0.93,-0.57) | -1.87(-2.02,-1.72) | -2.06(-2.35,-1.77) |
| Congo | -1.40(-1.45,-1.34) | -1.34(-1.45,-1.24) | -1.78(-1.94,-1.61) |
| Cook Islands | -0.28(-0.48,-0.08) | 1.18(0.82,1.54) | 0.52(0.23,0.81) |
| Costa Rica | -0.90(-1.01,-0.79) | -1.41(-1.81,-1.01) | -1.20(-1.51,-0.89) |
| Croatia | -0.69(-0.99,-0.38) | -3.47(-3.73,-3.22) | -3.42(-3.70,-3.15) |
| Cuba | -0.61(-0.72,-0.50) | -6.22(-7.39,-5.03) | -4.65(-5.16,-4.14) |
| Cyprus | -1.51(-1.90,-1.12) | -3.58(-4.11,-3.05) | -4.39(-4.62,-4.17) |
| Czech Republic | -3.08(-3.16,-2.99) | -5.98(-6.16,-5.79) | -6.04(-6.25,-5.84) |
| Democratic People's Republic of Korea | -0.60(-0.69,-0.51) | -0.24(-0.35,-0.13) | -0.49(-0.58,-0.40) |
| Democratic Republic of the Congo | -0.52(-0.59,-0.45) | -0.35(-0.43,-0.27) | -0.14(-0.22,-0.06) |
| Denmark | -1.56(-1.70,-1.41) | -2.77(-3.12,-2.43) | -4.01(-4.15,-3.87) |
| Djibouti | -0.27(-0.36,-0.17) | -0.81(-0.94,-0.67) | -1.23(-1.30,-1.17) |
| Dominica | -0.48(-0.59,-0.36) | 0.73(0.52,0.93) | -0.10(-0.19,-0.02) |
| Dominican Republic | -0.69(-0.81,-0.57) | -0.49(-0.99,0.01) | -0.72(-1.26,-0.16) |
| Ecuador | 0.08(-0.18,0.35) | -1.46(-1.87,-1.04) | -0.72(-1.24,-0.18) |
| Egypt | -0.19(-0.28,-0.10) | -1.78(-2.00,-1.56) | -2.35(-2.64,-2.05) |
| El Salvador | -0.84(-1.01,-0.68) | -1.99(-2.19,-1.79) | -0.57(-0.73,-0.41) |
| Equatorial Guinea | -1.85(-2.21,-1.49) | -3.19(-3.53,-2.84) | -3.98(-4.57,-3.39) |
| Eritrea | -1.30(-1.33,-1.26) | -1.30(-1.40,-1.19) | -1.83(-1.95,-1.71) |
| Estonia | 0.36(0.19,0.53) | 2.36(1.11,3.62) | 0.49(-0.75,1.76) |
| Ethiopia | -2.02(-2.12,-1.92) | -2.15(-2.32,-1.97) | -3.12(-3.28,-2.97) |
| Federated States of Micronesia | 0.43(0.33,0.52) | 2.04(1.82,2.25) | 1.50(1.34,1.66) |
| Fiji | -0.15(-0.37,0.07) | -0.17(-0.84,0.50) | 0.43(0.16,0.71) |
| Finland | -1.07(-1.68,-0.45) | -2.47(-2.65,-2.29) | -2.72(-3.03,-2.40) |
| France | -2.38(-2.47,-2.30) | -4.08(-4.20,-3.97) | -4.76(-4.91,-4.61) |
| Gabon | -1.04(-1.08,-1.00) | -1.18(-1.27,-1.10) | -1.48(-1.54,-1.42) |
| Georgia | 0.67(0.46,0.88) | 5.16(2.48,7.92) | 5.89(4.55,7.25) |
| Germany | -1.57(-1.77,-1.37) | -3.34(-3.46,-3.22) | -3.93(-4.10,-3.75) |
| Ghana | -0.68(-0.81,-0.54) | -0.10(-0.26,0.06) | -0.13(-0.44,0.19) |
| Greece | -0.12(-0.43,0.20) | -3.61(-3.97,-3.25) | -0.91(-1.11,-0.71) |
| Greenland | -0.35(-0.50,-0.20) | -3.39(-4.27,-2.50) | -2.16(-2.88,-1.43) |
| Grenada | -2.24(-2.57,-1.90) | -2.30(-3.34,-1.26) | -3.98(-5.12,-2.82) |
| Guam | -0.29(-0.53,-0.05) | -1.05(-1.44,-0.66) | 0.47(0.14,0.81) |
| Guatemala | -0.10(-0.27,0.07) | -3.15(-3.63,-2.66) | -3.16(-3.59,-2.74) |
| Guinea | -0.37(-0.46,-0.27) | -1.28(-1.45,-1.12) | -0.71(-0.88,-0.53) |
| Guinea-Bissau | -0.34(-0.37,-0.32) | -2.24(-2.43,-2.04) | -1.79(-2.06,-1.52) |
| Guyana | -0.63(-0.74,-0.52) | 2.18(1.08,3.29) | 0.36(-0.82,1.55) |
| Haiti | -0.75(-0.80,-0.70) | -0.84(-0.90,-0.78) | -1.15(-1.20,-1.09) |
| Honduras | -0.22(-0.34,-0.10) | -0.06(-0.22,0.09) | -0.63(-0.74,-0.52) |
| Hungary | -1.43(-1.52,-1.33) | -4.14(-4.30,-3.99) | -4.53(-4.69,-4.37) |
| Iceland | -0.19(-0.58,0.19) | -3.15(-3.33,-2.97) | -2.36(-2.46,-2.27) |
| India | -0.98(-1.08,-0.88) | -0.71(-0.79,-0.63) | -0.92(-1.02,-0.82) |
| Indonesia | 0.04(-0.15,0.22) | 0.34(0.13,0.56) | 0.13(0.00,0.25) |
| Iraq | 0.02(-0.06,0.10) | -0.25(-0.44,-0.05) | -1.06(-1.19,-0.93) |
| Ireland | -1.22(-1.54,-0.91) | -4.01(-4.12,-3.91) | -3.39(-3.50,-3.29) |
| Islamic Republic of Iran | -0.02(-0.25,0.22) | 3.35(2.75,3.95) | 2.96(2.46,3.45) |
| Israel | -0.58(-0.88,-0.28) | -4.17(-4.31,-4.03) | -2.71(-2.82,-2.60) |
| Italy | -3.69(-3.89,-3.50) | -5.32(-5.55,-5.10) | -6.58(-6.85,-6.31) |
| Jamaica | -2.47(-2.74,-2.20) | -3.37(-4.14,-2.60) | -4.19(-5.36,-3.01) |
| Japan | -1.32(-1.45,-1.18) | -2.59(-3.02,-2.17) | -4.47(-4.73,-4.22) |
| Jordan | 0.64(0.37,0.91) | 1.93(1.66,2.19) | 1.69(1.32,2.05) |
| Kazakhstan | -0.19(-0.32,-0.06) | -0.87(-1.23,-0.51) | -1.58(-2.13,-1.02) |
| Kenya | -0.31(-0.49,-0.13) | 0.41(0.29,0.53) | 0.23(-0.02,0.48) |
| Kingdom of Eswatini | -1.55(-1.74,-1.35) | -1.68(-1.87,-1.49) | -1.77(-2.13,-1.42) |
| Kiribati | 1.95(1.73,2.17) | 3.24(2.92,3.55) | 2.50(2.35,2.65) |
| Kuwait | -0.36(-0.52,-0.20) | -1.64(-2.55,-0.71) | -0.55(-1.05,-0.05) |
| Kyrgyzstan | -0.29(-0.45,-0.13) | 0.20(-0.24,0.64) | 0.84(0.48,1.20) |
| Lao People's Democratic Republic | -0.46(-0.55,-0.36) | -0.80(-0.96,-0.64) | -0.94(-1.06,-0.82) |
| Latvia | -0.44(-0.91,0.03) | -0.02(-0.92,0.88) | -0.65(-1.86,0.58) |
| Lebanon | -0.21(-0.27,-0.14) | -0.57(-0.69,-0.45) | -0.95(-1.11,-0.78) |
| Lesotho | 0.50(0.40,0.59) | 0.23(0.11,0.35) | 0.54(0.27,0.81) |
| Liberia | -0.58(-0.63,-0.52) | -1.54(-1.67,-1.41) | -1.46(-1.70,-1.22) |
| Libya | -0.31(-0.37,-0.24) | 3.69(3.33,4.06) | 2.98(2.77,3.19) |
| Lithuania | -0.81(-1.12,-0.50) | 1.37(0.29,2.46) | -0.23(-1.43,1.00) |
| Luxembourg | -0.88(-1.37,-0.40) | -3.96(-4.04,-3.88) | -3.13(-3.20,-3.06) |
| Madagascar | -0.61(-0.72,-0.51) | -0.48(-0.60,-0.36) | -0.90(-0.97,-0.82) |
| Malawi | -0.38(-0.48,-0.28) | 0.00(-0.22,0.22) | -0.56(-0.84,-0.28) |
| Malaysia | -0.37(-0.51,-0.22) | 1.45(0.96,1.95) | 0.46(0.16,0.76) |
| Maldives | -1.41(-1.51,-1.30) | -3.25(-3.46,-3.05) | -4.15(-4.29,-4.00) |
| Mali | -0.41(-0.47,-0.34) | -1.02(-1.12,-0.91) | -0.82(-0.97,-0.67) |
| Malta | -0.27(-0.81,0.28) | -4.27(-4.38,-4.17) | -3.08(-3.21,-2.96) |
| Marshall Islands | 0.76(0.62,0.90) | 1.94(1.62,2.26) | 1.63(1.41,1.85) |
| Mauritania | -0.49(-0.58,-0.39) | -2.53(-2.79,-2.26) | -2.50(-2.93,-2.07) |
| Mauritius | -0.33(-0.47,-0.20) | 1.99(0.99,3.00) | 1.37(0.47,2.29) |
| Mexico | -1.88(-2.06,-1.70) | -4.72(-5.00,-4.45) | -4.61(-4.80,-4.43) |
| Mongolia | -1.02(-1.13,-0.90) | 0.19(0.03,0.36) | 0.03(-0.16,0.21) |
| Montenegro | -0.25(-0.41,-0.09) | 0.68(0.04,1.33) | -1.23(-1.90,-0.56) |
| Morocco | -0.23(-0.29,-0.16) | 2.75(2.41,3.09) | 2.19(1.98,2.40) |
| Mozambique | -0.81(-0.95,-0.67) | 0.37(0.23,0.50) | 0.36(0.13,0.59) |
| Myanmar | -0.59(-0.68,-0.51) | -0.83(-0.98,-0.68) | -1.12(-1.25,-1.00) |
| Namibia | -1.63(-1.80,-1.45) | -0.92(-1.13,-0.71) | -1.46(-1.77,-1.15) |
| Nepal | 0.58(0.38,0.77) | -1.26(-1.45,-1.08) | -0.75(-1.00,-0.50) |
| Netherlands | -2.66(-2.76,-2.57) | -4.72(-4.96,-4.48) | -4.97(-5.24,-4.70) |
| New Zealand | 0.81(0.67,0.96) | 5.00(4.47,5.54) | 3.43(2.91,3.96) |
| Nicaragua | -0.89(-1.10,-0.67) | -1.76(-1.95,-1.57) | -1.22(-1.47,-0.96) |
| Niger | -0.55(-0.62,-0.47) | -1.74(-1.92,-1.57) | -1.52(-1.77,-1.27) |
| Nigeria | -0.68(-0.82,-0.55) | -1.01(-1.25,-0.77) | -2.09(-2.25,-1.92) |
| Northern Mariana Islands | 0.46(0.30,0.62) | -0.08(-0.54,0.39) | -0.38(-0.87,0.11) |
| Norway | -0.77(-1.11,-0.43) | -1.04(-1.84,-0.22) | -2.68(-3.62,-1.72) |
| Oman | -0.31(-0.41,-0.21) | 0.42(0.25,0.59) | -0.04(-0.15,0.06) |
| Pakistan | -0.91(-1.03,-0.78) | -0.61(-0.87,-0.35) | -1.42(-1.68,-1.16) |
| Palestine | -0.17(-0.28,-0.06) | 0.09(-0.13,0.30) | -0.37(-0.67,-0.06) |
| Panama | -0.67(-0.79,-0.55) | -1.25(-1.56,-0.94) | -0.77(-0.94,-0.61) |
| Papua New Guinea | 1.24(1.01,1.48) | 2.93(2.72,3.14) | 2.38(2.17,2.59) |
| Paraguay | -1.02(-1.19,-0.86) | 0.50(0.33,0.67) | 0.13(-0.16,0.43) |
| Peru | -1.54(-1.63,-1.45) | -0.24(-0.42,-0.05) | -0.64(-0.93,-0.35) |
| Philippines | -0.06(-0.28,0.16) | -3.20(-3.62,-2.78) | -2.42(-2.76,-2.08) |
| Plurinational State of Bolivia | -0.64(-0.71,-0.57) | -1.16(-1.27,-1.05) | -1.15(-1.24,-1.07) |
| Poland | -1.31(-1.52,-1.09) | -5.64(-6.36,-4.92) | -5.93(-6.52,-5.33) |
| Portugal | -2.78(-2.91,-2.65) | -3.89(-3.98,-3.80) | -3.94(-4.12,-3.77) |
| Principality of Monaco | -0.26(-0.32,-0.19) | -1.53(-1.65,-1.41) | -1.64(-1.78,-1.51) |
| Puerto Rico | -0.75(-0.85,-0.65) | -3.84(-4.71,-2.97) | -4.04(-4.85,-3.22) |
| Qatar | -0.04(-0.17,0.09) | 0.24(-0.11,0.59) | -0.07(-0.46,0.32) |
| Republic of Cabo Verde | -0.26(-0.38,-0.14) | -4.02(-4.59,-3.45) | -3.20(-4.05,-2.35) |
| Republic of C么te d'Ivoire | -0.49(-0.59,-0.39) | -1.66(-1.84,-1.49) | -2.23(-2.35,-2.12) |
| Republic of Korea | -2.21(-2.30,-2.13) | -2.77(-2.86,-2.69) | -3.46(-3.60,-3.33) |
| Republic of Moldova | -0.03(-0.19,0.12) | 1.19(0.10,2.28) | -0.05(-0.72,0.62) |
| Republic of Nauru | 0.05(-0.06,0.16) | 3.47(3.21,3.74) | 2.08(1.73,2.44) |
| Republic of Niue | 0.15(0.00,0.29) | 1.79(1.53,2.05) | 1.10(0.90,1.31) |
| Republic of Palau | 0.28(0.17,0.38) | 1.74(1.53,1.95) | 1.58(1.33,1.84) |
| Republic of San Marino | -0.38(-0.55,-0.21) | -2.02(-2.29,-1.75) | -2.49(-2.78,-2.19) |
| Republic of the Gambia | -0.42(-0.46,-0.38) | -1.62(-1.79,-1.45) | -1.67(-1.99,-1.36) |
| Romania | -2.89(-3.06,-2.73) | -4.96(-5.27,-4.65) | -4.88(-5.30,-4.46) |
| Russian Federation | -0.46(-0.89,-0.03) | 0.65(-0.92,2.24) | 0.63(-1.16,2.46) |
| Rwanda | -2.22(-2.37,-2.07) | -1.97(-2.38,-1.57) | -3.45(-3.88,-3.03) |
| Saint Kitts and Nevis | -0.68(-0.77,-0.59) | -1.92(-2.49,-1.34) | -2.30(-3.04,-1.56) |
| Saint Lucia | -1.69(-1.80,-1.57) | -7.28(-8.34,-6.21) | -7.77(-8.58,-6.95) |
| Saint Vincent and the Grenadines | -0.83(-0.94,-0.71) | 0.84(-1.49,3.23) | -2.36(-4.18,-0.50) |
| Samoa | 0.33(0.23,0.43) | 2.19(1.86,2.51) | 1.51(1.29,1.74) |
| Sao Tome and Principe | 0.41(0.31,0.51) | -0.52(-0.61,-0.43) | -0.80(-0.95,-0.64) |
| Saudi Arabia | -0.29(-0.39,-0.20) | 2.66(2.17,3.16) | 1.28(0.92,1.64) |
| Senegal | -0.57(-0.65,-0.49) | -2.10(-2.35,-1.85) | -1.89(-2.30,-1.48) |
| Serbia | -0.29(-0.74,0.16) | -2.63(-2.98,-2.27) | -2.70(-3.04,-2.36) |
| Seychelles | -0.34(-0.45,-0.23) | -0.62(-0.97,-0.26) | -0.94(-1.05,-0.83) |
| Sierra Leone | -0.43(-0.52,-0.34) | -2.16(-2.29,-2.03) | -1.76(-1.92,-1.59) |
| Singapore | 0.18(-0.05,0.41) | -3.34(-3.70,-2.99) | -2.26(-2.34,-2.17) |
| Slovakia | -0.86(-1.90,0.20) | -2.78(-3.26,-2.30) | -3.50(-3.89,-3.11) |
| Slovenia | -0.32(-0.41,-0.22) | -4.56(-4.82,-4.29) | -4.97(-5.21,-4.73) |
| Socialist Republic of Viet Nam | 0.32(0.14,0.50) | -0.28(-0.43,-0.14) | 0.10(-0.01,0.20) |
| Solomon Islands | 0.13(-0.02,0.29) | 2.84(2.67,3.00) | 2.21(1.96,2.47) |
| Somalia | -0.10(-0.28,0.08) | -1.16(-1.22,-1.11) | -1.22(-1.29,-1.15) |
| South Africa | -0.99(-1.36,-0.62) | -0.49(-0.81,-0.17) | -0.99(-1.48,-0.50) |
| South Sudan | -0.42(-0.58,-0.26) | -0.82(-1.02,-0.62) | -1.20(-1.53,-0.87) |
| Spain | -2.27(-2.35,-2.20) | -3.50(-3.61,-3.38) | -4.55(-4.71,-4.40) |
| Sri Lanka | -0.51(-0.65,-0.38) | -3.01(-3.42,-2.59) | -3.16(-3.58,-2.73) |
| Sudan | -0.32(-0.42,-0.22) | 2.20(1.99,2.40) | 1.72(1.50,1.93) |
| Suriname | -2.25(-2.41,-2.09) | -2.17(-2.60,-1.73) | -3.06(-3.45,-2.67) |
| Sweden | -1.87(-2.19,-1.55) | -3.27(-3.48,-3.05) | -3.09(-3.35,-2.82) |
| Switzerland | -2.04(-2.38,-1.71) | -4.10(-4.32,-3.89) | -4.54(-4.63,-4.44) |
| Syrian Arab Republic | -0.55(-0.73,-0.37) | 3.75(3.59,3.91) | 2.43(2.25,2.61) |
| Taiwan (Province of China) | -1.42(-1.67,-1.17) | 0.66(-0.35,1.69) | -2.27(-2.89,-1.64) |
| Tajikistan | -0.65(-0.82,-0.47) | 2.07(1.68,2.47) | 0.63(0.18,1.07) |
| Thailand | -0.36(-0.51,-0.22) | -2.61(-2.80,-2.42) | -1.78(-1.94,-1.62) |
| The former Yugoslav Republic of Macedonia | -0.95(-1.12,-0.78) | -1.95(-2.52,-1.38) | -3.29(-3.64,-2.95) |
| Timor-Leste | -0.32(-0.41,-0.23) | 0.10(0.03,0.17) | -0.01(-0.07,0.05) |
| Togo | -0.13(-0.21,-0.06) | -1.49(-1.57,-1.41) | -1.38(-1.50,-1.27) |
| Tokelau | -0.02(-0.17,0.13) | 1.72(1.47,1.97) | 1.32(1.01,1.62) |
| Tonga | 0.55(0.40,0.70) | 2.35(2.20,2.50) | 1.77(1.55,2.00) |
| Trinidad and Tobago | -0.49(-0.62,-0.35) | -3.45(-3.89,-3.02) | -2.41(-2.87,-1.95) |
| Tunisia | -0.29(-0.34,-0.24) | 2.44(2.10,2.77) | 1.91(1.71,2.12) |
| Turkey | 0.15(-0.47,0.77) | -1.58(-1.76,-1.39) | -1.56(-1.87,-1.24) |
| Turkmenistan | -0.33(-0.54,-0.11) | -3.71(-4.09,-3.33) | -2.83(-3.23,-2.44) |
| Tuvalu | 0.48(0.34,0.61) | 1.57(1.40,1.75) | 1.36(1.16,1.57) |
| Uganda | -1.50(-1.57,-1.42) | -1.35(-1.59,-1.10) | -2.18(-2.43,-1.93) |
| Ukraine | -3.77(-4.08,-3.44) | -6.22(-7.29,-5.13) | -8.60(-9.65,-7.55) |
| United Arab Emirates | -0.09(-0.22,0.05) | 3.71(3.24,4.17) | 2.29(1.79,2.80) |
| United Kingdom of Great Britain and Northern Ireland | -0.38(-0.49,-0.28) | -0.90(-1.02,-0.79) | -1.09(-1.22,-0.97) |
| United Republic of Tanzania | -0.87(-0.93,-0.80) | -1.22(-1.43,-1.02) | -1.96(-2.12,-1.79) |
| United States of America | -0.03(-0.31,0.25) | -3.71(-3.82,-3.60) | -3.35(-3.41,-3.28) |
| United States Virgin Islands | -1.17(-1.23,-1.10) | -2.49(-2.99,-1.99) | -3.11(-3.60,-2.62) |
| Uruguay | -0.36(-0.57,-0.15) | -1.51(-1.89,-1.13) | -1.91(-2.09,-1.74) |
| Uzbekistan | -0.04(-0.16,0.08) | -3.07(-3.74,-2.40) | -3.65(-4.49,-2.81) |
| Vanuatu | 0.31(0.20,0.42) | 0.40(0.35,0.45) | 1.49(1.22,1.77) |
| Yemen | -0.07(-0.11,-0.04) | 2.33(2.06,2.61) | 2.14(1.92,2.35) |
| Zambia | -1.27(-1.46,-1.07) | -0.21(-0.57,0.15) | -1.33(-1.67,-0.99) |
| Zimbabwe | -1.25(-1.51,-0.99) | 0.56(0.39,0.73) | 0.46(0.13,0.80) |

Note: EAPCs: Estimate the annual percentage change

**Table S3** Age-specific numbers and rates of incident and prevalent cases for Pneumoconiosis by gender in 2021 (Related to Figure 3).

| Age group | Incidence | | | | Prevalence | | | |
| --- | --- | --- | --- | --- | --- | --- | --- | --- |
|  | Number | | Rate/100, 000 | | Number | | Rate/100,000 | |
|  | Female | Male | Female | Male | Female | Male | Female | Male |
| <5 years | 0.00(0.00,0.00) | 0.00(0.00,0.00) | 0.00(0.00,0.00) | 0.00(0.00,0.00) | 0.00(0.00,0.00) | 0.00(0.00,0.0) | 0.00(0.00,0.00) | 0.00(0.00,0.00) |
| 5-9 years | 0.00(0.00,0.00) | 0.00(0.00,0.00) | 0.00(0.00,0.00) | 0.00(0.00,0.00) | 0.00(0.00,0.00) | 0.00(0.00,0.00) | 0.00(0.00,0.00) | 0.00(0.00,0.00) |
| 10-14 years | 0.00(0.00,0.00) | 0.00(0.00,0.00) | 0.00(0.00,0.00) | 0.00(0.00,0.00) | 0.00(0.00,0.00) | 0.00(0.00,0.00) | 0.00(0.00,0.00) | 0.00(0.00,0.00) |
| 15-19 years | 165.83(74.55,277.97) | 516.45(249.28,853.33) | 0.05(0.02,0.09) | 0.16(0.08,0.27) | 342.81(154.07,574.56) | 1067.57(515.28,1764.73) | 0.11(0.05,0.19) | 0.33(0.16,0.55) |
| 20-24 years | 266.94(150.94,414.44) | 803.43(497.42,1192.15) | 0.09(0.05,0.14) | 0.26(0.16,0.39) | 1486.02(733.53,2408.65) | 4479.48(2440.69,7162.83) | 0.51(0.25,0.82) | 1.48(0.80,2.36) |
| 25-29 years | 244.60(106.85,494.20) | 691.95(324.96,1274.03) | 0.08(0.04,0.17) | 0.23(0.11,0.43) | 2583.29(1503.07,4027.19) | 7097.86(4443.74,10669.66) | 0.89(0.52,1.38) | 2.39(1.49,3.59) |
| 30-34 years | 293.51(139.35,550.05) | 965.67(558.03,1541.23) | 0.10(0.05,0.18) | 0.32(0.18,0.50) | 3349.75(2047.42,5482.28) | 8599.80(5460.33,12662.39) | 1.12(0.68,1.83) | 2.81(1.79,4.14) |
| 35-39 years | 381.96(153.36,752.25) | 1442.80(705.29,2595.40) | 0.14(0.06,0.27) | 0.51(0.25,0.92) | 3348.13(2206.31,4955.99) | 9377.38(6496.27,13178.23) | 1.21(0.79,1.78) | 3.31(2.29,4.66) |
| 40-44 years | 487.80(268.85,813.92) | 1949.85(1269.32,2868.47) | 0.20(0.11,0.33) | 0.77(0.50,1.14) | 3235.42(2021.07,4964.59) | 10063.23(6395.87,14954.56) | 1.30(0.81,2.00) | 3.99(2.54,5.93) |
| 45-49 years | 645.24(281.17,1048.60) | 3001.05(1884.81,4320.55) | 0.27(0.12,0.44) | 1.26(0.79,1.82) | 3717.47(2370.23,5198.01) | 14389.47(10131.90,19437.73) | 1.58(1.01,2.21) | 6.05(4.26,8.17) |
| 50-54 years | 819.84(485.28,1229.13) | 4398.95(3229.46,5797.07) | 0.37(0.22,0.55) | 1.98(1.45,2.61) | 4535.92(2713.85,6718.26) | 22414.83(15210.48,30810.82) | 2.03(1.22,3.01) | 10.10(6.85,13.88) |
| 55-59 years | 969.72(488.33,1678.24) | 5423.74(3366.52,7915.31) | 0.48(0.24,0.83) | 2.79(1.73,4.06) | 5623.15(3872.82,7804.32) | 31668.94(23240.31,42332.91) | 2.80(1.93,3.88) | 16.26(11.93,21.74) |
| 60-64 years | 974.17(558.70,1477.80) | 5558.32(3927.35,7405.47) | 0.59(0.34,0.90) | 3.57(2.53,4.76) | 6165.66(3864.96,9031.05) | 35174.64(24973.06,48469.89) | 3.75(2.35,5.49) | 22.61(16.06,31.16) |
| 65-69 years | 1043.53(576.88,1657.28) | 7192.31(4608.37,10259.36) | 0.72(0.40,1.15) | 5.46(3.50,7.78) | 7289.76(5084.89,9909.51) | 50869.85(36958.39,66849.60) | 5.06(3.53,6.88) | 38.59(28.03,50.71) |
| 70-74 years | 919.29(568.92,1330.54) | 7023.43(5189.95,9211.39) | 0.84(0.52,1.22) | 7.29(5.38,9.56) | 6977.34(4686.15,9740.03) | 51260.88(35911.87,68056.98) | 6.38(4.28,8.90) | 53.18(37.26,70.60) |
| 75-79 years | 692.48(397.68,1035.68) | 5789.92(3884.83,7945.38) | 0.96(0.55,1.44) | 9.68(6.50,13.29) | 5398.70(3788.84,7477.06) | 41087.54(30188.65,54832.51) | 7.49(5.26,10.37) | 68.72(50.49,91.71) |
| 80+ years | 1381.80(1027.19,1772.43) | 8821.86(6898.42,10836.96) | 1.45(1.08,1.86) | 14.40(11.26,17.69) | 8514.27(6169.09,11492.09) | 46488.92(34003.71,61657.17) | 8.92(6.47,12.05) | 75.91(55.52,100.67) |

| Age group | DALYs | | | | Deaths | | | |
| --- | --- | --- | --- | --- | --- | --- | --- | --- |
|  | Number | | Rate/100,000 | | Number | | Rate/100,000 | |
|  | female | male | female | male | female | male | female | male |
| <5 years | 0.00(0.00,0.00) | 0.00(0.00,0.00) | 0.00(0.00,0.00) | 0.00(0.00,0.00) | — | — | — | — |
| 5-9 years | 0.00(0.00,0.00) | 0.00(0.00,0.00) | 0.00(0.00,0.00) | 0.00(0.00,0.00) | — | — | — | — |
| 10-14 years | 0.00(0.00,0.00) | 0.00(0.00,0.00) | 0.00(0.00,0.00) | 0.00(0.00,0.00) | — | — | — | — |
| 15-19 years | 455.02(241.78,719.46) | 665.81(519.03,862.14) | 0.15(0.08,0.24) | 0.21(0.16,0.27) | 5.50(2.58,9.38) | 6.82(5.55,8.52) | 0.00(0.00,0.00) | 0.00(0.00,0.00) |
| 20-24 years | 726.82(428.10,1103.35) | 1713.51(1302.88,2348.51) | 0.25(0.15,0.38) | 0.56(0.43,0.77) | 7.23(3.01,12.44) | 14.84(12.41,18.02) | 0.00(0.00,0.00) | 0.00(0.00,0.01) |
| 25-29 years | 993.68(605.34,1498.61) | 3280.22(2668.02,4133.10) | 0.34(0.21,0.52) | 1.10(0.90,1.39) | 9.31(4.54,15.28) | 34.42(28.54,39.71) | 0.00(0.00,0.01) | 0.01(0.01,0.01) |
| 30-34 years | 1575.85(901.69,2326.29) | 6294.32(5157.61,7576.22) | 0.53(0.30,0.78) | 2.06(1.69,2.48) | 18.11(7.87,29.20) | 85.58(68.65,105.16) | 0.01(0.00,0.01) | 0.03(0.02,0.03) |
| 35-39 years | 1753.22(1047.24,2530.20) | 11063.39(9121.81,13340.76) | 0.63(0.38,0.91) | 3.91(3.22,4.71) | 23.18(11.13,35.30) | 181.27(146.96,221.03) | 0.01(0.00,0.01) | 0.06(0.05,0.08) |
| 40-44 years | 1913.52(1213.63,2733.56) | 16324.55(13234.37,20212.40) | 0.77(0.49,1.10) | 6.47(5.25,8.02) | 29.34(14.75,45.94) | 308.13(246.70,390.40) | 0.01(0.01,0.02) | 0.12(0.10,0.15) |
| 45-49 years | 2315.80(1333.74,3250.40) | 24447.17(19247.38,31041.90) | 0.98(0.57,1.38) | 10.28(8.09,13.05) | 40.28(18.77,61.64) | 516.98(402.48,668.62) | 0.02(0.01,0.03) | 0.22(0.17,0.28) |
| 50-54 years | 3072.50(1902.48,4264.33) | 36601.86(29528.69,45419.69) | 1.38(0.85,1.91) | 16.49(13.30,20.46) | 61.75(30.71,93.35) | 866.28(672.94,1098.34) | 0.03(0.01,0.04) | 0.39(0.30,0.49) |
| 55-59 years | 4179.58(2438.12,5996.33) | 41026.70(34085.94,50026.43) | 2.08(1.21,2.98) | 21.07(17.50,25.69) | 98.23(46.31,148.48) | 1076.80(890.58,1333.71) | 0.05(0.02,0.07) | 0.55(0.46,0.68) |
| 60-64 years | 4474.62(2652.93,6710.77) | 41299.45(34256.95,48149.26) | 2.72(1.61,4.08) | 26.55(22.02,30.96) | 121.32(60.62,199.86) | 1247.62(1005.34,1475.17) | 0.07(0.04,0.12) | 0.80(0.65,0.95) |
| 65-69 years | 5639.10(3423.94,8605.87) | 49351.03(41963.56,58398.00) | 3.92(2.38,5.98) | 37.43(31.83,44.30) | 184.74(89.58,300.66) | 1719.98(1442.01,2060.94) | 0.13(0.06,0.21) | 1.30(1.09,1.56) |
| 70-74 years | 5895.30(3743.00,8468.01) | 54488.49(47299.25,62170.42) | 5.39(3.42,7.74) | 56.53(49.07,64.50) | 239.48(131.89,366.09) | 2350.65(2014.72,2745.33) | 0.22(0.12,0.33) | 2.44(2.09,2.85) |
| 75-79 years | 4978.88(3038.02,7314.94) | 47052.48(41118.98,53116.38) | 6.91(4.21,10.15) | 78.70(68.78,88.84) | 258.76(136.47,404.82) | 2580.10(2243.13,2945.01) | 0.36(0.19,0.56) | 4.32(3.75,4.93) |
| 80+ years | 7847.88(5234.09,10984.55) | 67633.78(59369.57,75036.27) | 8.23(5.49,11.51) | 110.43(96.94,122.52) | 603.99(369.27,861.90) | 5631.84(4925.58,6218.37) | 0.63(0.39,0.90) | 9.20(8.04,10.15) |

**Table S4**. Pneumoconiosis age-specific DALYs, Deaths numbers and rates by gender in 2021 (Related to Figure 4).

|  | **ASIR / 100,000 (95% CI)** | | **ASPR / 100,000 (95% CI)** | | **ASDR / 100,000 (95% CI)** | | **AS-DALYs rate / 100,000 (95% CI)** | |
| --- | --- | --- | --- | --- | --- | --- | --- | --- |
|  | **Male** | **Female** | **Male** | **Female** | **Male** | **Female** | **Male** | **Female** |
| Andean Latin America | 0.58(0.51,0.64) | 0.29(0.25,0.34) | 2.29(1.86,2.78) | 1.42(1.15,1.71) | 0.32(0.22,0.46) | 0.10(0.05,0.16) | 7.24(5.14,10.17) | 4.15(0.28,21.31) |
| Australasia | 1.07(0.98,1.18) | 0.06(0.04,0.07) | 3.77(3.05,4.73) | 0.30(0.23,0.38) | 0.68(0.58,0.79) | 0.02(0.01,0.02) | 10.71(9.29,12.24) | 3.98(2.32,6.27) |
| Caribbean | 0.23(0.18,0.28) | 0.13(0.10,0.18) | 1.25(0.97,1.59) | 0.84(0.64,1.08) | 0.04(0.03,0.04) | 0.01(0.01,0.01) | 1.03(0.82,1.31) | 1.48(1.31,1.70) |
| Central Asia | 0.48(0.41,0.54) | 0.33(0.27,0.39) | 2.51(2.02,3.12) | 2.23(1.80,2.72) | 0.20(0.15,0.26) | 0.08(0.05,0.12) | 4.77(3.75,6.17) | 0.39(0.31,0.51) |
| Central Europe | 0.88(0.76,1.03) | 0.18(0.15,0.22) | 7.33(6.12,8.86) | 1.49(1.20,1.84) | 0.18(0.16,0.21) | 0.03(0.02,0.03) | 4.71(4.19,5.44) | 1.14(0.94,1.40) |
| Central Latin America | 0.99(0.83,1.15) | 0.38(0.31,0.47) | 7.79(6.35,9.29) | 3.02(2.43,3.69) | 0.16(0.14,0.18) | 0.04(0.03,0.04) | 4.57(4.01,5.20) | 0.71(0.62,0.82) |
| Central Sub-Saharan Africa | 0.71(0.63,0.80) | 0.22(0.18,0.26) | 1.60(1.29,1.94) | 0.48(0.37,0.61) | 0.75(0.39,1.25) | 0.22(0.01,1.18) | 16.52(8.52,27.78) | 2.06(1.45,2.89) |
| East Asia | 2.73(2.36,3.14) | 0.25(0.21,0.29) | 19.00(15.31,23.38) | 1.94(1.58,2.35) | 0.87(0.67,1.12) | 0.04(0.02,0.07) | 21.49(16.72,27.20) | 0.29(0.23,0.36) |
| Eastern Europe | 0.44(0.37,0.52) | 0.18(0.15,0.23) | 2.46(1.97,3.02) | 1.12(0.88,1.42) | 0.09(0.08,0.10) | 0.03(0.02,0.03) | 2.30(1.98,2.61) | 0.29(0.22,0.38) |
| Eastern Sub-Saharan Africa | 0.74(0.65,0.84) | 0.12(0.09,0.14) | 2.11(1.74,2.50) | 0.26(0.20,0.35) | 0.55(0.30,0.84) | 0.11(0.01,0.41) | 11.92(6.68,18.20) | 2.31(1.37,3.71) |
| Global | 1.36(1.19,1.53) | 0.21(0.17,0.25) | 8.36(6.96,10.03) | 1.40(1.15,1.70) | 0.46(0.40,0.52) | 0.04(0.02,0.05) | 10.15(8.79,11.63) | 0.56(0.39,0.79) |
| High-income Asia Pacific | 1.19(1.06,1.34) | 0.18(0.14,0.22) | 4.72(4.04,5.54) | 1.46(1.14,1.84) | 0.51(0.44,0.57) | 0.01(0.01,0.02) | 8.68(7.72,9.76) | 1.84(0.63,4.39) |
| High-income North America | 1.29(1.11,1.49) | 0.19(0.15,0.24) | 9.24(7.55,11.10) | 1.50(1.20,1.87) | 0.27(0.24,0.29) | 0.01(0.01,0.01) | 5.30(4.75,5.93) | 2.58(0.22,6.34) |
| North Africa and Middle East | 0.45(0.38,0.54) | 0.18(0.14,0.23) | 3.18(2.52,4.00) | 1.02(0.79,1.31) | 0.13(0.11,0.16) | 0.09(0.05,0.14) | 3.39(2.84,4.05) | 2.07(0.17,7.63) |
| Oceania | 1.02(0.85,1.22) | 0.24(0.20,0.29) | 6.14(4.86,7.58) | 1.13(0.89,1.40) | 0.01(0.00,0.01) | 0.10(0.03,0.23) | 1.01(0.69,1.46) | 0.49(0.40,0.60) |
| South Asia | 0.84(0.74,0.98) | 0.18(0.14,0.22) | 3.25(2.63,4.02) | 0.67(0.51,0.87) | 0.27(0.17,0.37) | 0.04(0.02,0.08) | 5.71(3.79,7.76) | 1.01(0.67,1.43) |
| Southeast Asia | 0.35(0.28,0.43) | 0.25(0.20,0.31) | 2.40(1.88,2.96) | 2.00(1.61,2.46) | 0.01(0.01,0.01) | 0.01(0.01,0.02) | 0.62(0.48,0.82) | 2.07(1.11,3.25) |
| Southern Latin America | 0.90(0.83,1.00) | 0.16(0.13,0.21) | 5.33(4.51,6.20) | 1.14(0.92,1.41) | 0.37(0.32,0.43) | 0.02(0.01,0.02) | 7.38(6.38,8.40) | 0.42(0.34,0.52) |
| Southern Sub-Saharan Africa | 1.23(1.08,1.40) | 0.35(0.30,0.41) | 2.42(2.00,2.97) | 1.29(1.02,1.64) | 0.95(0.84,1.05) | 0.19(0.11,0.32) | 20.56(18.08,23.10) | 0.71(0.59,0.87) |
| Tropical Latin America | 0.74(0.65,0.85) | 0.29(0.24,0.34) | 4.66(3.93,5.45) | 1.90(1.58,2.29) | 0.29(0.27,0.32) | 0.06(0.05,0.07) | 8.20(7.63,8.82) | 0.47(0.36,0.61) |
| Western Europe | 1.07(0.97,1.18) | 0.18(0.14,0.22) | 4.58(3.82,5.39) | 1.31(1.05,1.61) | 0.44(0.39,0.48) | 0.01(0.01,0.01) | 7.23(6.58,7.84) | 1.22(0.78,1.87) |
| Western Sub-Saharan Africa | 0.17(0.13,0.22) | 0.08(0.06,0.11) | 0.68(0.51,0.87) | 0.31(0.22,0.41) | 0.71(0.46,1.03) | 0.13(0.01,0.34) | 14.26(9.06,21.30) | 0.83(0.48,1.53) |

**Table S5** Socio-demographic Index values for all estimated GBD 2021 locations

| **Socio-demographic Index values for all estimated GBD 2021 locations, 1990-2005** | | | | | | | | | | | | | | | | |
| --- | --- | --- | --- | --- | --- | --- | --- | --- | --- | --- | --- | --- | --- | --- | --- | --- |
| **Location** | **1990** | **1991** | **1992** | **1993** | **1994** | **1995** | **1996** | **1997** | **1998** | **1999** | **2000** | **2001** | **2002** | **2003** | **2004** | **2005** |
| Central Asia | 0.5534 | 0.5551 | 0.5573 | 0.5601 | 0.5627 | 0.5656 | 0.5688 | 0.5718 | 0.5747 | 0.5781 | 0.5822 | 0.5866 | 0.5915 | 0.5965 | 0.6017 | 0.6073 |
| Central Europe | 0.6373 | 0.6431 | 0.6488 | 0.6545 | 0.6612 | 0.6680 | 0.6741 | 0.6800 | 0.6860 | 0.6921 | 0.6988 | 0.7057 | 0.7120 | 0.7178 | 0.7235 | 0.7287 |
| Eastern Europe | 0.6643 | 0.6715 | 0.6788 | 0.6834 | 0.6860 | 0.6895 | 0.6928 | 0.6952 | 0.6969 | 0.6988 | 0.7010 | 0.7035 | 0.7067 | 0.7120 | 0.7192 | 0.7263 |
| Australasia | 0.7312 | 0.7349 | 0.7390 | 0.7434 | 0.7477 | 0.7520 | 0.7564 | 0.7610 | 0.7653 | 0.7695 | 0.7738 | 0.7781 | 0.7825 | 0.7863 | 0.7894 | 0.7917 |
| High-income Asia Pacific | 0.7678 | 0.7733 | 0.7788 | 0.7840 | 0.7890 | 0.7941 | 0.7989 | 0.8031 | 0.8068 | 0.8105 | 0.8141 | 0.8175 | 0.8211 | 0.8246 | 0.8281 | 0.8311 |
| High-income North America | 0.7657 | 0.7680 | 0.7715 | 0.7750 | 0.7782 | 0.7813 | 0.7841 | 0.7863 | 0.7886 | 0.7912 | 0.7950 | 0.7993 | 0.8030 | 0.8058 | 0.8080 | 0.8089 |
| Southern Latin America | 0.5873 | 0.5921 | 0.5981 | 0.6036 | 0.6094 | 0.6144 | 0.6193 | 0.6248 | 0.6303 | 0.6357 | 0.6412 | 0.6460 | 0.6498 | 0.6526 | 0.6557 | 0.6605 |
| Western Europe | 0.7464 | 0.7517 | 0.7570 | 0.7621 | 0.7667 | 0.7706 | 0.7741 | 0.7776 | 0.7809 | 0.7840 | 0.7874 | 0.7909 | 0.7942 | 0.7970 | 0.7997 | 0.8024 |
| Caribbean | 0.5181 | 0.5228 | 0.5270 | 0.5305 | 0.5336 | 0.5367 | 0.5398 | 0.5433 | 0.5474 | 0.5521 | 0.5574 | 0.5631 | 0.5691 | 0.5749 | 0.5805 | 0.5858 |
| Andean Latin America | 0.5000 | 0.5017 | 0.5037 | 0.5064 | 0.5103 | 0.5149 | 0.5194 | 0.5240 | 0.5284 | 0.5327 | 0.5376 | 0.5426 | 0.5479 | 0.5531 | 0.5582 | 0.5631 |
| Central Latin America | 0.4858 | 0.4896 | 0.4941 | 0.4994 | 0.5051 | 0.5099 | 0.5147 | 0.5202 | 0.5261 | 0.5318 | 0.5373 | 0.5424 | 0.5471 | 0.5514 | 0.5560 | 0.5607 |
| Tropical Latin America | 0.4996 | 0.5043 | 0.5081 | 0.5116 | 0.5153 | 0.5193 | 0.5235 | 0.5278 | 0.5322 | 0.5367 | 0.5417 | 0.5469 | 0.5524 | 0.5577 | 0.5633 | 0.5689 |
| North Africa and Middle East | 0.4374 | 0.4459 | 0.4540 | 0.4621 | 0.4704 | 0.4786 | 0.4865 | 0.4940 | 0.5014 | 0.5090 | 0.5168 | 0.5241 | 0.5311 | 0.5383 | 0.5457 | 0.5530 |
| South Asia | 0.3198 | 0.3259 | 0.3319 | 0.3380 | 0.3443 | 0.3507 | 0.3573 | 0.3637 | 0.3703 | 0.3770 | 0.3834 | 0.3895 | 0.3952 | 0.4010 | 0.4072 | 0.4140 |
| East Asia | 0.4712 | 0.4799 | 0.4884 | 0.4969 | 0.5049 | 0.5135 | 0.5238 | 0.5338 | 0.5427 | 0.5514 | 0.5588 | 0.5642 | 0.5708 | 0.5789 | 0.5877 | 0.5971 |
| Oceania | 0.3912 | 0.3944 | 0.3976 | 0.4011 | 0.4049 | 0.4085 | 0.4124 | 0.4159 | 0.4192 | 0.4225 | 0.4251 | 0.4270 | 0.4285 | 0.4301 | 0.4316 | 0.4332 |
| Southeast Asia | 0.4641 | 0.4717 | 0.4795 | 0.4873 | 0.4953 | 0.5034 | 0.5112 | 0.5187 | 0.5244 | 0.5297 | 0.5346 | 0.5390 | 0.5435 | 0.5481 | 0.5527 | 0.5574 |
| Central sub-Saharan Africa | 0.3024 | 0.3052 | 0.3078 | 0.3091 | 0.3099 | 0.3111 | 0.3127 | 0.3145 | 0.3165 | 0.3186 | 0.3208 | 0.3237 | 0.3276 | 0.3319 | 0.3374 | 0.3441 |
| Eastern sub-Saharan Africa | 0.2336 | 0.2363 | 0.2386 | 0.2408 | 0.2429 | 0.2454 | 0.2486 | 0.2521 | 0.2559 | 0.2600 | 0.2642 | 0.2688 | 0.2735 | 0.2784 | 0.2839 | 0.2900 |
| Southern sub-Saharan Africa | 0.5069 | 0.5124 | 0.5176 | 0.5228 | 0.5281 | 0.5334 | 0.5388 | 0.5441 | 0.5491 | 0.5539 | 0.5585 | 0.5625 | 0.5660 | 0.5692 | 0.5724 | 0.5761 |
| Western sub-Saharan Africa | 0.2737 | 0.2774 | 0.2810 | 0.2844 | 0.2877 | 0.2910 | 0.2946 | 0.2983 | 0.3020 | 0.3058 | 0.3098 | 0.3140 | 0.3189 | 0.3242 | 0.3302 | 0.3368 |
| **Socio-demographic Index values for all estimated GBD 2021 locations, 2006-2021** | | | | | | | | | | | | | | | | |
| **Location** | **2006** | **2007** | **2008** | **2009** | **2010** | **2011** | **2012** | **2013** | **2014** | **2015** | **2016** | **2017** | **2018** | **2019** | **2020** | **2021** |
| Central Asia | 0.6131 | 0.6191 | 0.6249 | 0.6301 | 0.6353 | 0.6403 | 0.6447 | 0.6489 | 0.6530 | 0.6568 | 0.6603 | 0.6636 | 0.6667 | 0.6698 | 0.6725 | 0.6752 |
| Central Europe | 0.7335 | 0.7381 | 0.7431 | 0.7488 | 0.7549 | 0.7603 | 0.7648 | 0.7686 | 0.7718 | 0.7748 | 0.7778 | 0.7814 | 0.7855 | 0.7897 | 0.7931 | 0.7962 |
| Eastern Europe | 0.7326 | 0.7388 | 0.7455 | 0.7511 | 0.7566 | 0.7610 | 0.7654 | 0.7703 | 0.7751 | 0.7797 | 0.7848 | 0.7900 | 0.7945 | 0.7981 | 0.8005 | 0.8029 |
| Australasia | 0.7929 | 0.7939 | 0.7964 | 0.7999 | 0.8041 | 0.8082 | 0.8125 | 0.8173 | 0.8216 | 0.8256 | 0.8295 | 0.8329 | 0.8366 | 0.8406 | 0.8433 | 0.8455 |
| High-income Asia Pacific | 0.8338 | 0.8365 | 0.8393 | 0.8418 | 0.8447 | 0.8477 | 0.8507 | 0.8537 | 0.8566 | 0.8596 | 0.8627 | 0.8659 | 0.8691 | 0.8722 | 0.8744 | 0.8768 |
| High-income North America | 0.8097 | 0.8125 | 0.8171 | 0.8225 | 0.8278 | 0.8321 | 0.8357 | 0.8391 | 0.8423 | 0.8459 | 0.8494 | 0.8530 | 0.8563 | 0.8597 | 0.8619 | 0.8635 |
| Southern Latin America | 0.6644 | 0.6674 | 0.6707 | 0.6742 | 0.6788 | 0.6841 | 0.6884 | 0.6917 | 0.6960 | 0.7033 | 0.7108 | 0.7175 | 0.7250 | 0.7311 | 0.7340 | 0.7360 |
| Western Europe | 0.8051 | 0.8077 | 0.8105 | 0.8131 | 0.8161 | 0.8194 | 0.8226 | 0.8258 | 0.8286 | 0.8313 | 0.8342 | 0.8375 | 0.8408 | 0.8442 | 0.8466 | 0.8487 |
| Caribbean | 0.5906 | 0.5947 | 0.5983 | 0.6017 | 0.6055 | 0.6095 | 0.6133 | 0.6169 | 0.6204 | 0.6241 | 0.6276 | 0.6308 | 0.6340 | 0.6373 | 0.6397 | 0.6420 |
| Andean Latin America | 0.5680 | 0.5730 | 0.5786 | 0.5840 | 0.5902 | 0.5969 | 0.6038 | 0.6107 | 0.6171 | 0.6228 | 0.6282 | 0.6335 | 0.6389 | 0.6439 | 0.6478 | 0.6516 |
| Central Latin America | 0.5656 | 0.5707 | 0.5758 | 0.5799 | 0.5843 | 0.5893 | 0.5948 | 0.6007 | 0.6065 | 0.6118 | 0.6171 | 0.6224 | 0.6278 | 0.6328 | 0.6370 | 0.6407 |
| Tropical Latin America | 0.5747 | 0.5809 | 0.5874 | 0.5933 | 0.5996 | 0.6060 | 0.6118 | 0.6174 | 0.6226 | 0.6274 | 0.6315 | 0.6358 | 0.6402 | 0.6447 | 0.6486 | 0.6524 |
| North Africa and Middle East | 0.5601 | 0.5665 | 0.5723 | 0.5771 | 0.5818 | 0.5876 | 0.5941 | 0.6011 | 0.6084 | 0.6158 | 0.6231 | 0.6306 | 0.6379 | 0.6451 | 0.6517 | 0.6582 |
| South Asia | 0.4214 | 0.4293 | 0.4370 | 0.4452 | 0.4541 | 0.4633 | 0.4731 | 0.4834 | 0.4941 | 0.5049 | 0.5153 | 0.5251 | 0.5345 | 0.5432 | 0.5506 | 0.5579 |
| East Asia | 0.6073 | 0.6175 | 0.6270 | 0.6363 | 0.6480 | 0.6575 | 0.6629 | 0.6687 | 0.6741 | 0.6773 | 0.6815 | 0.6897 | 0.6986 | 0.7083 | 0.7177 | 0.7257 |
| Oceania | 0.4346 | 0.4361 | 0.4374 | 0.4388 | 0.4408 | 0.4426 | 0.4445 | 0.4466 | 0.4494 | 0.4524 | 0.4555 | 0.4584 | 0.4608 | 0.4633 | 0.4655 | 0.4674 |
| Southeast Asia | 0.5623 | 0.5677 | 0.5732 | 0.5785 | 0.5843 | 0.5904 | 0.5968 | 0.6031 | 0.6094 | 0.6157 | 0.6218 | 0.6280 | 0.6341 | 0.6401 | 0.6451 | 0.6498 |
| Central sub-Saharan Africa | 0.3515 | 0.3596 | 0.3685 | 0.3767 | 0.3854 | 0.3947 | 0.4042 | 0.4135 | 0.4226 | 0.4310 | 0.4387 | 0.4462 | 0.4532 | 0.4602 | 0.4665 | 0.4723 |
| Eastern sub-Saharan Africa | 0.2965 | 0.3036 | 0.3109 | 0.3185 | 0.3263 | 0.3343 | 0.3416 | 0.3492 | 0.3570 | 0.3649 | 0.3728 | 0.3805 | 0.3884 | 0.3963 | 0.4035 | 0.4097 |
| Southern sub-Saharan Africa | 0.5807 | 0.5857 | 0.5904 | 0.5946 | 0.5988 | 0.6033 | 0.6080 | 0.6128 | 0.6175 | 0.6218 | 0.6259 | 0.6298 | 0.6334 | 0.6369 | 0.6399 | 0.6422 |
| Western sub-Saharan Africa | 0.3432 | 0.3500 | 0.3567 | 0.3635 | 0.3706 | 0.3776 | 0.3848 | 0.3919 | 0.3992 | 0.4062 | 0.4129 | 0.4196 | 0.4264 | 0.4334 | 0.4398 | 0.4460 |

**Table S6** Concentration curves(**A**) and regression curves(**B**) of Pneumoconiosis

**A**

| **Year** | **CI_Mean** | **CI_Lower** | **CI_Upper** | **num** |
| --- | --- | --- | --- | --- |
| 1990 | -0.144271379 | -0.238568053 | -0.031601328 | -0.14(-0.24, -0.03) |
| 2021 | -0.291541341 | -0.346848604 | -0.227325411 | -0.29(-0.35, -0.23) |

B

| **Year** | **Coefficient** | **Lower_CI** | **Upper_CI** | **Type** | **num** |
| --- | --- | --- | --- | --- | --- |
| 1990 | -7.091693353 | -9.606589003 | -4.576797703 | Slope | -7.09(-9.61, -4.58) |
| 2021 | -5.148045382 | -6.439143701 | -3.856947064 | Slope | -5.15(-6.44, -3.86) |

**Table S7** Decomposition analysis

|  | Overall Difference | Aging | Population | Epidemiological Change | Aging  Percentage | Population  Percentage | Epidemiological Change  Percentage |
| --- | --- | --- | --- | --- | --- | --- | --- |
| Global | -14898.8 | 180446.5 | 191774.1 | -387119 | -1211.15 | -1287.18 | 2598.33 |
| Middle SDI | 10422.04 | 83427.95 | 65818.16 | -138824 | 800.5 | 631.53 | -1332.02 |
| Low-middle SDI | 23757.15 | 21356.62 | 18261.6 | -15861.1 | 89.9 | 76.87 | -66.76 |
| High SDI | -36178.8 | 25636.99 | 44255.55 | -106071 | -70.86 | -122.32 | 293.19 |
| High-middle SDI | -22947 | 35410.65 | 52572.83 | -110931 | -154.31 | -229.11 | 483.42 |
| Low SDI | 10232.61 | 10951.55 | 9946.41 | -10665.3 | 107.03 | 97.2 | -104.23 |
| Andean Latin America | 1293.81 | 1297.69 | 805.75 | -809.63 | 100.3 | 62.28 | -62.58 |
| Australasia | 2053.24 | 800.39 | 663.98 | 588.87 | 38.98 | 32.34 | 28.68 |
| Caribbean | 14.84 | 87.74 | 136.14 | -209.04 | 591.37 | 917.62 | -1408.99 |
| Central Asia | 138.86 | 442.18 | 1058.7 | -1362.02 | 318.43 | 762.41 | -980.84 |
| Central Europe | -10140.2 | -519.37 | 4504.41 | -14125.3 | 5.12 | -44.42 | 139.3 |
| Central Latin America | 484.69 | 4933.04 | 2936.17 | -7384.53 | 1017.77 | 605.78 | -1523.55 |
| Central Sub-Saharan Africa | 2799.27 | 1995.42 | 1509.5 | -705.64 | 71.28 | 53.92 | -25.21 |
| East Asia | 4747.11 | 109561.4 | 96806.66 | -201621 | 2307.96 | 2039.28 | -4247.24 |
| Eastern Europe | -6924.25 | -1576.42 | 3351.11 | -8698.95 | 22.77 | -48.4 | 125.63 |
| Eastern Sub-Saharan Africa | 3721.64 | 4298.18 | 3834.71 | -4411.24 | 115.49 | 103.04 | -118.53 |
| High-income Asia Pacific | -4014.24 | 9624.47 | 9499.34 | -23138.1 | -239.76 | -236.64 | 576.4 |
| High-income North America | -8230.18 | 5224.47 | 9245.56 | -22700.2 | -63.48 | -112.34 | 275.82 |
| North Africa and Middle East | 4874.54 | 6822.81 | 4822.76 | -6771.03 | 139.97 | 98.94 | -138.91 |
| Oceania | 62.09 | 413.2 | 94.25 | -445.35 | 665.44 | 151.78 | -717.22 |
| South Asia | 21184.59 | 20307.53 | 14133.3 | -13256.2 | 95.86 | 66.71 | -62.57 |
| Southeast Asia | 1922.52 | 1333.89 | 1161.87 | -573.24 | 69.38 | 60.43 | -29.82 |
| Southern Latin America | -48.05 | 781.64 | 1254.31 | -2084.01 | -1626.57 | -2610.19 | 4336.76 |
| Southern Sub-Saharan Africa | 2737.61 | 1791.62 | 1919.44 | -973.45 | 65.44 | 70.11 | -35.56 |
| Tropical Latin America | 6266.47 | 4302.43 | 3295.8 | -1331.76 | 68.66 | 52.59 | -21.25 |
| Western Europe | -42048.8 | 3859.58 | 23938.77 | -69847.2 | -9.18 | -56.93 | 166.11 |
| Western Sub-Saharan Africa | 4205.68 | 5837.07 | 5408.38 | -7039.77 | 138.79 | 128.6 | -167.39 |

**Table S8** Forecast of ASIR, ASDR of Pneumoconiosis

| ASIR | Year | low_50 | up_50 | low_60 | up_60 | low_70 | up_70 | low_80 | up_80 | low_95 | up_95 |
| --- | --- | --- | --- | --- | --- | --- | --- | --- | --- | --- | --- |
| 1.44 | 1990 | 1.44 | 1.45 | 1.44 | 1.45 | 1.44 | 1.45 | 1.43 | 1.45 | 1.43 | 1.46 |
| 1.43 | 1991 | 1.42 | 1.43 | 1.42 | 1.43 | 1.42 | 1.44 | 1.42 | 1.44 | 1.42 | 1.44 |
| 1.41 | 1992 | 1.41 | 1.42 | 1.41 | 1.42 | 1.41 | 1.42 | 1.41 | 1.42 | 1.40 | 1.43 |
| 1.40 | 1993 | 1.39 | 1.40 | 1.39 | 1.40 | 1.39 | 1.40 | 1.39 | 1.41 | 1.39 | 1.41 |
| 1.38 | 1994 | 1.38 | 1.39 | 1.38 | 1.39 | 1.38 | 1.39 | 1.37 | 1.39 | 1.37 | 1.39 |
| 1.37 | 1995 | 1.36 | 1.37 | 1.36 | 1.37 | 1.36 | 1.37 | 1.36 | 1.37 | 1.35 | 1.38 |
| 1.35 | 1996 | 1.34 | 1.35 | 1.34 | 1.35 | 1.34 | 1.35 | 1.34 | 1.36 | 1.34 | 1.36 |
| 1.33 | 1997 | 1.32 | 1.33 | 1.32 | 1.33 | 1.32 | 1.33 | 1.32 | 1.34 | 1.32 | 1.34 |
| 1.31 | 1998 | 1.30 | 1.31 | 1.30 | 1.31 | 1.30 | 1.31 | 1.30 | 1.31 | 1.30 | 1.32 |
| 1.29 | 1999 | 1.28 | 1.29 | 1.28 | 1.29 | 1.28 | 1.29 | 1.28 | 1.29 | 1.28 | 1.30 |
| 1.27 | 2000 | 1.27 | 1.27 | 1.26 | 1.27 | 1.26 | 1.27 | 1.26 | 1.28 | 1.26 | 1.28 |
| 1.25 | 2001 | 1.25 | 1.26 | 1.25 | 1.26 | 1.25 | 1.26 | 1.25 | 1.26 | 1.24 | 1.26 |
| 1.23 | 2002 | 1.23 | 1.24 | 1.23 | 1.24 | 1.23 | 1.24 | 1.23 | 1.24 | 1.22 | 1.25 |
| 1.22 | 2003 | 1.21 | 1.22 | 1.21 | 1.22 | 1.21 | 1.22 | 1.21 | 1.22 | 1.21 | 1.23 |
| 1.20 | 2004 | 1.20 | 1.21 | 1.20 | 1.21 | 1.20 | 1.21 | 1.20 | 1.21 | 1.19 | 1.21 |
| 1.19 | 2005 | 1.18 | 1.19 | 1.18 | 1.19 | 1.18 | 1.19 | 1.18 | 1.19 | 1.18 | 1.20 |
| 1.18 | 2006 | 1.18 | 1.18 | 1.18 | 1.18 | 1.17 | 1.18 | 1.17 | 1.19 | 1.17 | 1.19 |
| 1.17 | 2007 | 1.17 | 1.18 | 1.17 | 1.18 | 1.17 | 1.18 | 1.17 | 1.18 | 1.16 | 1.18 |
| 1.17 | 2008 | 1.17 | 1.17 | 1.17 | 1.17 | 1.16 | 1.17 | 1.16 | 1.18 | 1.16 | 1.18 |
| 1.16 | 2009 | 1.16 | 1.17 | 1.16 | 1.17 | 1.16 | 1.17 | 1.16 | 1.17 | 1.16 | 1.17 |
| 1.15 | 2010 | 1.15 | 1.16 | 1.15 | 1.16 | 1.15 | 1.16 | 1.15 | 1.16 | 1.15 | 1.16 |
| 1.14 | 2011 | 1.14 | 1.14 | 1.14 | 1.14 | 1.13 | 1.14 | 1.13 | 1.15 | 1.13 | 1.15 |
| 1.12 | 2012 | 1.12 | 1.12 | 1.12 | 1.12 | 1.11 | 1.12 | 1.11 | 1.13 | 1.11 | 1.13 |
| 1.10 | 2013 | 1.10 | 1.10 | 1.09 | 1.10 | 1.09 | 1.10 | 1.09 | 1.10 | 1.09 | 1.11 |
| 1.08 | 2014 | 1.08 | 1.08 | 1.08 | 1.08 | 1.07 | 1.08 | 1.07 | 1.08 | 1.07 | 1.09 |
| 1.07 | 2015 | 1.06 | 1.07 | 1.06 | 1.07 | 1.06 | 1.07 | 1.06 | 1.07 | 1.06 | 1.07 |
| 1.05 | 2016 | 1.05 | 1.06 | 1.05 | 1.06 | 1.05 | 1.06 | 1.05 | 1.06 | 1.05 | 1.06 |
| 1.05 | 2017 | 1.04 | 1.05 | 1.04 | 1.05 | 1.04 | 1.05 | 1.04 | 1.05 | 1.04 | 1.05 |
| 1.04 | 2018 | 1.04 | 1.04 | 1.03 | 1.04 | 1.03 | 1.04 | 1.03 | 1.04 | 1.03 | 1.05 |
| 1.03 | 2019 | 1.03 | 1.04 | 1.03 | 1.04 | 1.03 | 1.04 | 1.03 | 1.04 | 1.03 | 1.04 |
| 1.03 | 2020 | 1.03 | 1.04 | 1.03 | 1.04 | 1.03 | 1.04 | 1.03 | 1.04 | 1.03 | 1.04 |
| 1.03 | 2021 | 1.03 | 1.03 | 1.03 | 1.04 | 1.03 | 1.04 | 1.03 | 1.04 | 1.02 | 1.04 |
| 1.01 | 2022 | 1.00 | 1.02 | 1.00 | 1.02 | 1.00 | 1.03 | 1.00 | 1.03 | 0.99 | 1.04 |
| 1.00 | 2023 | 0.99 | 1.01 | 0.99 | 1.01 | 0.99 | 1.02 | 0.98 | 1.02 | 0.97 | 1.03 |
| 0.99 | 2024 | 0.98 | 1.00 | 0.98 | 1.01 | 0.97 | 1.01 | 0.97 | 1.01 | 0.96 | 1.02 |
| 0.98 | 2025 | 0.97 | 0.99 | 0.97 | 1.00 | 0.96 | 1.00 | 0.96 | 1.00 | 0.95 | 1.02 |
| 0.97 | 2026 | 0.96 | 0.98 | 0.95 | 0.99 | 0.95 | 0.99 | 0.95 | 1.00 | 0.93 | 1.01 |
| 0.96 | 2027 | 0.95 | 0.98 | 0.94 | 0.98 | 0.94 | 0.98 | 0.93 | 0.99 | 0.92 | 1.00 |
| 0.95 | 2028 | 0.94 | 0.97 | 0.93 | 0.97 | 0.93 | 0.98 | 0.92 | 0.98 | 0.91 | 1.00 |
| 0.94 | 2029 | 0.93 | 0.96 | 0.92 | 0.96 | 0.92 | 0.97 | 0.91 | 0.97 | 0.90 | 0.99 |
| 0.93 | 2030 | 0.92 | 0.95 | 0.91 | 0.96 | 0.91 | 0.96 | 0.90 | 0.97 | 0.89 | 0.98 |
| 0.93 | 2031 | 0.91 | 0.94 | 0.90 | 0.95 | 0.90 | 0.95 | 0.89 | 0.96 | 0.87 | 0.98 |
| 0.92 | 2032 | 0.90 | 0.94 | 0.89 | 0.94 | 0.89 | 0.95 | 0.88 | 0.95 | 0.86 | 0.97 |
| 0.91 | 2033 | 0.89 | 0.93 | 0.89 | 0.93 | 0.88 | 0.94 | 0.87 | 0.95 | 0.85 | 0.97 |
| 0.90 | 2034 | 0.88 | 0.92 | 0.88 | 0.93 | 0.87 | 0.93 | 0.86 | 0.94 | 0.84 | 0.96 |
| 0.89 | 2035 | 0.87 | 0.92 | 0.87 | 0.92 | 0.86 | 0.93 | 0.85 | 0.93 | 0.83 | 0.96 |

| ASDR | Year | low_50 | up_50 | low_60 | up_60 | low_70 | up_70 | low_80 | up_80 | low_95 | up_95 |
| --- | --- | --- | --- | --- | --- | --- | --- | --- | --- | --- | --- |
| 0.65 | 1990 | 0.65 | 0.66 | 0.65 | 0.66 | 0.65 | 0.66 | 0.65 | 0.66 | 0.64 | 0.66 |
| 0.64 | 1991 | 0.64 | 0.64 | 0.64 | 0.64 | 0.64 | 0.64 | 0.64 | 0.65 | 0.63 | 0.65 |
| 0.63 | 1992 | 0.62 | 0.63 | 0.62 | 0.63 | 0.62 | 0.63 | 0.62 | 0.63 | 0.62 | 0.64 |
| 0.62 | 1993 | 0.62 | 0.62 | 0.61 | 0.62 | 0.61 | 0.62 | 0.61 | 0.62 | 0.61 | 0.63 |
| 0.61 | 1994 | 0.60 | 0.61 | 0.60 | 0.61 | 0.60 | 0.61 | 0.60 | 0.61 | 0.60 | 0.61 |
| 0.60 | 1995 | 0.60 | 0.60 | 0.60 | 0.60 | 0.60 | 0.60 | 0.59 | 0.60 | 0.59 | 0.61 |
| 0.58 | 1996 | 0.58 | 0.59 | 0.58 | 0.59 | 0.58 | 0.59 | 0.58 | 0.59 | 0.58 | 0.59 |
| 0.57 | 1997 | 0.56 | 0.57 | 0.56 | 0.57 | 0.56 | 0.57 | 0.56 | 0.57 | 0.56 | 0.57 |
| 0.56 | 1998 | 0.55 | 0.56 | 0.55 | 0.56 | 0.55 | 0.56 | 0.55 | 0.56 | 0.55 | 0.56 |
| 0.55 | 1999 | 0.54 | 0.55 | 0.54 | 0.55 | 0.54 | 0.55 | 0.54 | 0.55 | 0.54 | 0.55 |
| 0.54 | 2000 | 0.54 | 0.54 | 0.53 | 0.54 | 0.53 | 0.54 | 0.53 | 0.54 | 0.53 | 0.54 |
| 0.53 | 2001 | 0.52 | 0.53 | 0.52 | 0.53 | 0.52 | 0.53 | 0.52 | 0.53 | 0.52 | 0.53 |
| 0.52 | 2002 | 0.51 | 0.52 | 0.51 | 0.52 | 0.51 | 0.52 | 0.51 | 0.52 | 0.51 | 0.52 |
| 0.51 | 2003 | 0.51 | 0.51 | 0.50 | 0.51 | 0.50 | 0.51 | 0.50 | 0.51 | 0.50 | 0.51 |
| 0.49 | 2004 | 0.49 | 0.50 | 0.49 | 0.50 | 0.49 | 0.50 | 0.49 | 0.50 | 0.49 | 0.50 |
| 0.48 | 2005 | 0.48 | 0.48 | 0.48 | 0.48 | 0.47 | 0.48 | 0.47 | 0.48 | 0.47 | 0.48 |
| 0.45 | 2006 | 0.45 | 0.46 | 0.45 | 0.46 | 0.45 | 0.46 | 0.45 | 0.46 | 0.45 | 0.46 |
| 0.44 | 2007 | 0.44 | 0.44 | 0.44 | 0.44 | 0.43 | 0.44 | 0.43 | 0.44 | 0.43 | 0.44 |
| 0.42 | 2008 | 0.42 | 0.43 | 0.42 | 0.43 | 0.42 | 0.43 | 0.42 | 0.43 | 0.42 | 0.43 |
| 0.41 | 2009 | 0.41 | 0.41 | 0.41 | 0.42 | 0.41 | 0.42 | 0.41 | 0.42 | 0.41 | 0.42 |
| 0.40 | 2010 | 0.40 | 0.41 | 0.40 | 0.41 | 0.40 | 0.41 | 0.40 | 0.41 | 0.40 | 0.41 |
| 0.39 | 2011 | 0.39 | 0.39 | 0.39 | 0.39 | 0.39 | 0.39 | 0.39 | 0.40 | 0.39 | 0.40 |
| 0.38 | 2012 | 0.38 | 0.38 | 0.38 | 0.38 | 0.38 | 0.38 | 0.38 | 0.38 | 0.38 | 0.39 |
| 0.37 | 2013 | 0.37 | 0.37 | 0.37 | 0.37 | 0.37 | 0.37 | 0.37 | 0.37 | 0.37 | 0.37 |
| 0.36 | 2014 | 0.36 | 0.36 | 0.36 | 0.36 | 0.36 | 0.36 | 0.36 | 0.36 | 0.36 | 0.36 |
| 0.35 | 2015 | 0.35 | 0.35 | 0.35 | 0.35 | 0.35 | 0.35 | 0.35 | 0.35 | 0.35 | 0.36 |
| 0.34 | 2016 | 0.34 | 0.35 | 0.34 | 0.35 | 0.34 | 0.35 | 0.34 | 0.35 | 0.34 | 0.35 |
| 0.34 | 2017 | 0.33 | 0.34 | 0.33 | 0.34 | 0.33 | 0.34 | 0.33 | 0.34 | 0.33 | 0.34 |
| 0.33 | 2018 | 0.32 | 0.33 | 0.32 | 0.33 | 0.32 | 0.33 | 0.32 | 0.33 | 0.32 | 0.33 |
| 0.32 | 2019 | 0.32 | 0.32 | 0.32 | 0.32 | 0.32 | 0.32 | 0.32 | 0.32 | 0.32 | 0.32 |
| 0.31 | 2020 | 0.31 | 0.31 | 0.31 | 0.31 | 0.31 | 0.31 | 0.31 | 0.32 | 0.31 | 0.32 |
| 0.31 | 2021 | 0.31 | 0.31 | 0.31 | 0.31 | 0.31 | 0.31 | 0.30 | 0.31 | 0.30 | 0.31 |
| 0.29 | 2022 | 0.29 | 0.30 | 0.29 | 0.30 | 0.29 | 0.30 | 0.29 | 0.30 | 0.29 | 0.30 |
| 0.28 | 2023 | 0.28 | 0.29 | 0.28 | 0.29 | 0.28 | 0.29 | 0.28 | 0.29 | 0.27 | 0.29 |
| 0.28 | 2024 | 0.27 | 0.28 | 0.27 | 0.28 | 0.27 | 0.28 | 0.27 | 0.28 | 0.26 | 0.29 |
| 0.27 | 2025 | 0.26 | 0.27 | 0.26 | 0.27 | 0.26 | 0.27 | 0.26 | 0.28 | 0.26 | 0.28 |
| 0.26 | 2026 | 0.25 | 0.26 | 0.25 | 0.27 | 0.25 | 0.27 | 0.25 | 0.27 | 0.25 | 0.27 |
| 0.25 | 2027 | 0.25 | 0.26 | 0.25 | 0.26 | 0.24 | 0.26 | 0.24 | 0.26 | 0.24 | 0.27 |
| 0.24 | 2028 | 0.24 | 0.25 | 0.24 | 0.25 | 0.24 | 0.25 | 0.23 | 0.25 | 0.23 | 0.26 |
| 0.24 | 2029 | 0.23 | 0.24 | 0.23 | 0.24 | 0.23 | 0.24 | 0.23 | 0.25 | 0.22 | 0.25 |
| 0.23 | 2030 | 0.22 | 0.24 | 0.22 | 0.24 | 0.22 | 0.24 | 0.22 | 0.24 | 0.21 | 0.25 |
| 0.22 | 2031 | 0.22 | 0.23 | 0.22 | 0.23 | 0.21 | 0.23 | 0.21 | 0.23 | 0.21 | 0.24 |
| 0.22 | 2032 | 0.21 | 0.22 | 0.21 | 0.22 | 0.21 | 0.23 | 0.21 | 0.23 | 0.20 | 0.23 |
| 0.21 | 2033 | 0.20 | 0.22 | 0.20 | 0.22 | 0.20 | 0.22 | 0.20 | 0.22 | 0.19 | 0.23 |
| 0.20 | 2034 | 0.20 | 0.21 | 0.20 | 0.21 | 0.20 | 0.21 | 0.19 | 0.22 | 0.19 | 0.22 |
| 0.20 | 2035 | 0.19 | 0.20 | 0.19 | 0.21 | 0.19 | 0.21 | 0.19 | 0.21 | 0.18 | 0.22 |

**Table S9** Number of incidence cases and number of death cases of Pneumoconiosis

| Incidence Number | Year | group | low_50 | up_50 | low_60 | up_60 | low_70 | up_70 | low_80 | up_80 | low_95 | up_95 |
| --- | --- | --- | --- | --- | --- | --- | --- | --- | --- | --- | --- | --- |
| 51868.63 | 1990 | Number | 51646.22 | 52091.04 | 51591.12 | 52146.15 | 51526.77 | 52210.50 | 51445.59 | 52291.67 | 51221.87 | 52515.40 |
| 52307.77 | 1991 | Number | 52087.41 | 52528.13 | 52032.81 | 52582.73 | 51969.06 | 52646.48 | 51888.63 | 52726.91 | 51666.97 | 52948.58 |
| 52661.26 | 1992 | Number | 52441.05 | 52881.47 | 52386.49 | 52936.03 | 52322.78 | 52999.74 | 52242.41 | 53080.11 | 52020.90 | 53301.62 |
| 52953.22 | 1993 | Number | 52732.80 | 53173.65 | 52678.18 | 53228.26 | 52614.41 | 53292.03 | 52533.96 | 53372.49 | 52312.23 | 53594.22 |
| 53220.63 | 1994 | Number | 52999.92 | 53441.35 | 52945.23 | 53496.04 | 52881.37 | 53559.89 | 52800.81 | 53640.45 | 52578.79 | 53862.48 |
| 53468.82 | 1995 | Number | 53247.79 | 53689.86 | 53193.02 | 53744.62 | 53129.07 | 53808.57 | 53048.40 | 53889.24 | 52826.06 | 54111.59 |
| 53648.37 | 1996 | Number | 53427.18 | 53869.57 | 53372.37 | 53924.37 | 53308.37 | 53988.37 | 53227.64 | 54069.10 | 53005.13 | 54291.61 |
| 53734.45 | 1997 | Number | 53513.32 | 53955.58 | 53458.53 | 54010.37 | 53394.55 | 54074.35 | 53313.84 | 54155.06 | 53091.39 | 54377.51 |
| 53794.42 | 1998 | Number | 53573.38 | 54015.46 | 53518.61 | 54070.23 | 53454.66 | 54134.18 | 53373.98 | 54214.85 | 53151.63 | 54437.20 |
| 53917.89 | 1999 | Number | 53696.81 | 54138.98 | 53642.03 | 54193.76 | 53578.07 | 54257.72 | 53497.38 | 54338.41 | 53274.98 | 54560.81 |
| 54133.14 | 2000 | Number | 53911.79 | 54354.48 | 53856.95 | 54409.33 | 53792.91 | 54473.37 | 53712.12 | 54554.16 | 53489.46 | 54776.82 |
| 54404.37 | 2001 | Number | 54182.68 | 54626.06 | 54127.75 | 54680.99 | 54063.61 | 54745.13 | 53982.69 | 54826.05 | 53759.69 | 55049.06 |
| 54636.29 | 2002 | Number | 54414.37 | 54858.21 | 54359.39 | 54913.19 | 54295.18 | 54977.39 | 54214.19 | 55058.39 | 53990.95 | 55281.62 |
| 54866.28 | 2003 | Number | 54644.14 | 55088.42 | 54589.09 | 55143.46 | 54524.82 | 55207.73 | 54443.75 | 55288.81 | 54220.28 | 55512.27 |
| 55157.36 | 2004 | Number | 54934.89 | 55379.84 | 54879.76 | 55434.97 | 54815.39 | 55499.33 | 54734.19 | 55580.54 | 54510.39 | 55804.34 |
| 55523.79 | 2005 | Number | 55300.83 | 55746.75 | 55245.58 | 55802.00 | 55181.07 | 55866.51 | 55099.69 | 55947.88 | 54875.41 | 56172.17 |
| 56076.39 | 2006 | Number | 55852.66 | 56300.12 | 55797.22 | 56355.55 | 55732.49 | 56420.28 | 55650.83 | 56501.94 | 55425.77 | 56727.00 |
| 56776.41 | 2007 | Number | 56551.72 | 57001.11 | 56496.04 | 57056.79 | 56431.03 | 57121.80 | 56349.02 | 57203.81 | 56122.99 | 57429.84 |
| 57526.18 | 2008 | Number | 57300.40 | 57751.96 | 57244.46 | 57807.91 | 57179.14 | 57873.23 | 57096.73 | 57955.64 | 56869.61 | 58182.76 |
| 58216.15 | 2009 | Number | 57989.42 | 58442.88 | 57933.24 | 58499.06 | 57867.65 | 58564.65 | 57784.89 | 58647.40 | 57556.82 | 58875.48 |
| 58637.54 | 2010 | Number | 58410.43 | 58864.64 | 58354.16 | 58920.91 | 58288.46 | 58986.62 | 58205.57 | 59069.51 | 57977.12 | 59297.96 |
| 58712.75 | 2011 | Number | 58486.09 | 58939.42 | 58429.93 | 58995.58 | 58364.35 | 59061.16 | 58281.62 | 59143.89 | 58053.61 | 59371.89 |
| 58521.13 | 2012 | Number | 58295.53 | 58746.73 | 58239.63 | 58802.63 | 58174.36 | 58867.90 | 58092.02 | 58950.24 | 57865.08 | 59177.18 |
| 58219.55 | 2013 | Number | 57995.21 | 58443.89 | 57939.63 | 58499.47 | 57874.72 | 58564.38 | 57792.84 | 58646.26 | 57567.17 | 58871.93 |
| 58002.24 | 2014 | Number | 57778.98 | 58225.50 | 57723.67 | 58280.82 | 57659.07 | 58345.41 | 57577.59 | 58426.90 | 57353.00 | 58651.49 |
| 58023.32 | 2015 | Number | 57800.59 | 58246.05 | 57745.40 | 58301.24 | 57680.96 | 58365.68 | 57599.67 | 58446.97 | 57375.61 | 58671.02 |
| 58227.75 | 2016 | Number | 58005.19 | 58450.30 | 57950.05 | 58505.45 | 57885.66 | 58569.84 | 57804.43 | 58651.07 | 57580.55 | 58874.95 |
| 58469.59 | 2017 | Number | 58247.13 | 58692.06 | 58192.01 | 58747.18 | 58127.64 | 58811.55 | 58046.45 | 58892.74 | 57822.66 | 59116.53 |
| 58816.63 | 2018 | Number | 58593.99 | 59039.28 | 58538.83 | 59094.44 | 58474.41 | 59158.86 | 58393.15 | 59240.12 | 58169.19 | 59464.08 |
| 59356.19 | 2019 | Number | 59132.91 | 59579.47 | 59077.58 | 59634.80 | 59012.98 | 59699.39 | 58931.49 | 59780.89 | 58706.88 | 60005.50 |
| 60087.32 | 2020 | Number | 59862.62 | 60312.02 | 59806.94 | 60367.70 | 59741.93 | 60432.71 | 59659.92 | 60514.72 | 59433.89 | 60740.75 |
| 60661.43 | 2021 | Number | 60433.91 | 60888.95 | 60377.54 | 60945.32 | 60311.71 | 61011.15 | 60228.67 | 61094.19 | 59999.80 | 61323.06 |
| 61186.2 | 2022 | Number | 60626.37 | 61746.04 | 60487.65 | 61884.75 | 60325.68 | 62046.72 | 60121.35 | 62251.05 | 59558.20 | 62814.21 |
| 61284.04 | 2023 | Number | 60640.24 | 61927.84 | 60480.72 | 62087.36 | 60294.45 | 62273.62 | 60059.48 | 62508.60 | 59411.85 | 63156.22 |
| 61388.27 | 2024 | Number | 60665.94 | 62110.60 | 60486.97 | 62289.57 | 60277.99 | 62498.55 | 60014.35 | 62762.19 | 59287.74 | 63488.80 |
| 61498.02 | 2025 | Number | 60700.46 | 62295.59 | 60502.84 | 62493.20 | 60272.09 | 62723.95 | 59981.00 | 63015.05 | 59178.70 | 63817.35 |
| 61613.04 | 2026 | Number | 60742.25 | 62483.83 | 60526.49 | 62699.59 | 60274.55 | 62951.52 | 59956.72 | 63269.35 | 59080.77 | 64145.31 |
| 61727.75 | 2027 | Number | 60785.22 | 62670.28 | 60551.68 | 62903.82 | 60278.99 | 63176.51 | 59934.98 | 63520.52 | 58986.86 | 64468.65 |
| 61840.02 | 2028 | Number | 60827.56 | 62852.48 | 60576.69 | 63103.34 | 60283.77 | 63396.27 | 59914.24 | 63765.80 | 58895.76 | 64784.27 |
| 61951.52 | 2029 | Number | 60870.39 | 63032.64 | 60602.51 | 63300.52 | 60289.72 | 63613.31 | 59895.13 | 64007.91 | 58807.58 | 65095.45 |
| 62060.86 | 2030 | Number | 60911.62 | 63210.10 | 60626.87 | 63494.85 | 60294.38 | 63827.34 | 59874.92 | 64246.79 | 58718.87 | 65402.85 |
| 62168.44 | 2031 | Number | 60951.26 | 63385.63 | 60649.67 | 63687.22 | 60297.52 | 64039.37 | 59853.26 | 64483.63 | 58628.85 | 65708.04 |
| 62274.53 | 2032 | Number | 60989.42 | 63559.63 | 60671.01 | 63878.05 | 60299.20 | 64249.85 | 59830.16 | 64718.89 | 58537.43 | 66011.63 |
| 62343.63 | 2033 | Number | 60991.84 | 63695.42 | 60656.90 | 64030.36 | 60265.81 | 64421.45 | 59772.43 | 64914.84 | 58412.62 | 66274.65 |
| 62395.62 | 2034 | Number | 60977.75 | 63813.49 | 60626.44 | 64164.80 | 60216.22 | 64575.02 | 59698.72 | 65092.52 | 58272.44 | 66518.81 |
| 62433.96 | 2035 | Number | 60950.05 | 63917.87 | 60582.38 | 64285.54 | 60153.06 | 64714.86 | 59611.46 | 65256.47 | 58118.74 | 66749.18 |

| Death Number | Year | low_50 | up_50 | low_60 | up_60 | low_70 | up_70 | low_80 | up_80 | low_95 | up_95 |
| --- | --- | --- | --- | --- | --- | --- | --- | --- | --- | --- | --- |
| 23468.31 | 1990 | 23318.53 | 23618.09 | 23281.42 | 23655.20 | 23238.08 | 23698.54 | 23183.42 | 23753.21 | 23032.75 | 23903.87 |
| 23443.62 | 1991 | 23298.50 | 23588.74 | 23262.54 | 23624.70 | 23220.56 | 23666.69 | 23167.59 | 23719.65 | 23021.61 | 23865.64 |
| 23374.37 | 1992 | 23230.53 | 23518.22 | 23194.89 | 23553.86 | 23153.27 | 23595.47 | 23100.77 | 23647.97 | 22956.07 | 23792.67 |
| 23400.18 | 1993 | 23256.60 | 23543.76 | 23221.03 | 23579.34 | 23179.49 | 23620.87 | 23127.09 | 23673.28 | 22982.66 | 23817.71 |
| 23373.78 | 1994 | 23230.53 | 23517.04 | 23195.03 | 23552.53 | 23153.59 | 23593.98 | 23101.30 | 23646.26 | 22957.20 | 23790.37 |
| 23454.52 | 1995 | 23311.14 | 23597.90 | 23275.61 | 23633.43 | 23234.13 | 23674.91 | 23181.80 | 23727.24 | 23037.56 | 23871.47 |
| 23226.83 | 1996 | 23084.40 | 23369.26 | 23049.11 | 23404.55 | 23007.91 | 23445.76 | 22955.92 | 23497.74 | 22812.65 | 23641.01 |
| 22963.69 | 1997 | 22822.29 | 23105.08 | 22787.26 | 23140.12 | 22746.35 | 23181.02 | 22694.74 | 23232.63 | 22552.50 | 23374.87 |
| 22861.12 | 1998 | 22720.21 | 23002.03 | 22685.29 | 23036.95 | 22644.52 | 23077.72 | 22593.09 | 23129.15 | 22451.34 | 23270.90 |
| 22881.11 | 1999 | 22740.28 | 23021.94 | 22705.38 | 23056.83 | 22664.64 | 23097.58 | 22613.24 | 23148.98 | 22471.57 | 23290.65 |
| 22931.8 | 2000 | 22790.94 | 23072.65 | 22756.04 | 23107.55 | 22715.29 | 23148.31 | 22663.88 | 23199.72 | 22522.19 | 23341.41 |
| 22885.1 | 2001 | 22744.56 | 23025.63 | 22709.74 | 23060.46 | 22669.08 | 23101.12 | 22617.78 | 23152.41 | 22476.41 | 23293.78 |
| 22845.7 | 2002 | 22705.42 | 22985.97 | 22670.67 | 23020.73 | 22630.08 | 23061.31 | 22578.89 | 23112.51 | 22437.78 | 23253.62 |
| 22876.65 | 2003 | 22736.34 | 23016.96 | 22701.58 | 23051.73 | 22660.98 | 23092.32 | 22609.77 | 23143.53 | 22468.63 | 23284.68 |
| 22710.04 | 2004 | 22570.48 | 22849.61 | 22535.90 | 22884.19 | 22495.52 | 22924.56 | 22444.58 | 22975.50 | 22304.19 | 23115.90 |
| 22326.44 | 2005 | 22188.51 | 22464.37 | 22154.33 | 22498.54 | 22114.42 | 22538.45 | 22064.08 | 22588.79 | 21925.33 | 22727.54 |
| 21607.47 | 2006 | 21472.30 | 21742.63 | 21438.81 | 21776.13 | 21399.70 | 21815.23 | 21350.37 | 21864.57 | 21214.40 | 22000.53 |
| 21154.61 | 2007 | 21021.28 | 21287.94 | 20988.25 | 21320.97 | 20949.67 | 21359.55 | 20901.01 | 21408.21 | 20766.89 | 21542.33 |
| 20870.49 | 2008 | 20738.48 | 21002.50 | 20705.77 | 21035.21 | 20667.58 | 21073.40 | 20619.40 | 21121.58 | 20486.60 | 21254.38 |
| 20654 | 2009 | 20523.07 | 20784.93 | 20490.63 | 20817.38 | 20452.74 | 20855.26 | 20404.96 | 20903.05 | 20273.24 | 21034.76 |
| 20491.12 | 2010 | 20361.07 | 20621.16 | 20328.85 | 20653.39 | 20291.23 | 20691.01 | 20243.76 | 20738.48 | 20112.94 | 20869.29 |
| 20212.53 | 2011 | 20083.80 | 20341.26 | 20051.91 | 20373.16 | 20014.66 | 20410.40 | 19967.68 | 20457.38 | 19838.19 | 20586.88 |
| 19887.84 | 2012 | 19760.58 | 20015.11 | 19729.05 | 20046.64 | 19692.23 | 20083.46 | 19645.78 | 20129.91 | 19517.76 | 20257.93 |
| 19612.54 | 2013 | 19486.59 | 19738.49 | 19455.38 | 19769.70 | 19418.94 | 19806.14 | 19372.97 | 19852.11 | 19246.27 | 19978.81 |
| 19344.5 | 2014 | 19219.82 | 19469.18 | 19188.93 | 19500.07 | 19152.86 | 19536.14 | 19107.35 | 19581.64 | 18981.94 | 19707.06 |
| 19151.28 | 2015 | 19027.59 | 19274.97 | 18996.94 | 19305.62 | 18961.16 | 19341.40 | 18916.01 | 19386.55 | 18791.59 | 19510.97 |
| 18971.69 | 2016 | 18848.95 | 19094.43 | 18818.54 | 19124.85 | 18783.03 | 19160.36 | 18738.23 | 19205.16 | 18614.76 | 19328.62 |
| 18744.07 | 2017 | 18622.42 | 18865.72 | 18592.27 | 18895.86 | 18557.08 | 18931.06 | 18512.68 | 18975.46 | 18390.30 | 19097.83 |
| 18490.95 | 2018 | 18370.52 | 18611.38 | 18340.68 | 18641.22 | 18305.84 | 18676.06 | 18261.88 | 18720.01 | 18140.74 | 18841.16 |
| 18313.1 | 2019 | 18193.52 | 18432.69 | 18163.89 | 18462.32 | 18129.29 | 18496.91 | 18085.65 | 18540.56 | 17965.35 | 18660.85 |
| 18161.6 | 2020 | 18042.26 | 18280.94 | 18012.69 | 18310.51 | 17978.16 | 18345.04 | 17934.60 | 18388.60 | 17814.55 | 18508.65 |
| 18058.14 | 2021 | 17936.42 | 18179.86 | 17906.26 | 18210.01 | 17871.05 | 18245.23 | 17826.62 | 18289.65 | 17704.19 | 18412.09 |
| 17750.01 | 2022 | 17549.42 | 17950.60 | 17499.72 | 18000.30 | 17441.69 | 18058.34 | 17368.48 | 18131.55 | 17166.70 | 18333.33 |
| 17405.38 | 2023 | 17173.77 | 17636.99 | 17116.39 | 17694.37 | 17049.38 | 17761.38 | 16964.85 | 17845.91 | 16731.86 | 18078.89 |
| 17075.21 | 2024 | 16817.66 | 17332.77 | 16753.84 | 17396.59 | 16679.32 | 17471.10 | 16585.32 | 17565.10 | 16326.24 | 17824.19 |
| 16760.83 | 2025 | 16480.29 | 17041.36 | 16410.78 | 17110.87 | 16329.62 | 17192.03 | 16227.23 | 17294.42 | 15945.03 | 17576.62 |
| 16457.35 | 2026 | 16155.33 | 16759.38 | 16080.50 | 16834.21 | 15993.12 | 16921.59 | 15882.89 | 17031.82 | 15579.07 | 17335.64 |
| 16150 | 2027 | 15827.42 | 16472.58 | 15747.50 | 16552.51 | 15654.17 | 16645.84 | 15536.43 | 16763.57 | 15211.94 | 17088.07 |
| 15839.75 | 2028 | 15499.08 | 16180.41 | 15414.67 | 16264.82 | 15316.11 | 16363.38 | 15191.77 | 16487.72 | 14849.08 | 16830.41 |
| 15534.19 | 2029 | 15177.59 | 15890.79 | 15089.23 | 15979.14 | 14986.06 | 16082.31 | 14855.91 | 16212.47 | 14497.19 | 16571.18 |
| 15245.78 | 2030 | 14874.26 | 15617.30 | 14782.21 | 15709.35 | 14674.72 | 15816.84 | 14539.12 | 15952.44 | 14165.40 | 16326.16 |
| 14974.01 | 2031 | 14587.83 | 15360.18 | 14492.15 | 15455.87 | 14380.42 | 15567.60 | 14239.47 | 15708.55 | 13851.00 | 16097.01 |
| 14705.07 | 2032 | 14304.45 | 15105.69 | 14205.19 | 15204.95 | 14089.28 | 15320.85 | 13943.06 | 15467.07 | 13540.07 | 15870.07 |
| 14420.91 | 2033 | 14007.86 | 14833.97 | 13905.52 | 14936.31 | 13786.02 | 15055.81 | 13635.26 | 15206.57 | 13219.75 | 15622.08 |
| 14134.31 | 2034 | 13710.44 | 14558.17 | 13605.42 | 14663.20 | 13482.78 | 14785.83 | 13328.08 | 14940.54 | 12901.70 | 15366.92 |
| 13862.93 | 2035 | 13428.71 | 14297.16 | 13321.12 | 14404.74 | 13195.49 | 14530.37 | 13037.00 | 14688.86 | 12600.20 | 15125.66 |
